# Coastal Transient Niches Shape the Microdiversity Pattern of a Bacterioplankton Population with Reduced Genomes

**DOI:** 10.1128/mbio.00571-22

**Published:** 2022-07-26

**Authors:** Xiao Chu, Xiaojun Wang, Lok Shan Cheung, Xiaoyuan Feng, Put Ang, Shing Yip Lee, Sean A. Crowe, Haiwei Luo

**Affiliations:** a Simon F. S. Li Marine Science Laboratory, School of Life Sciences, The Chinese University of Hong Kong, Shatin, Hong Kong SAR; b State Key Laboratory of Agrobiotechnology, The Chinese University of Hong Kong, Shatin, Hong Kong SAR; c Shenzhen Research Institute, The Chinese University of Hong Kong, Shenzhen, China; d Department of Earth Sciences, The University of Hong Kong, Pok Fu Lam, Hong Kong SAR; e Institute of Space and Earth Information Science, The Chinese University of Hong Kong, Shatin, Hong Kong SAR; f The Swire Institute of Marine Science, The University of Hong Kong, Cape d’Aguilar Road, Shek O, Hong Kong SAR; g Departments of Microbiology and Immunology, and Earth, Ocean, and Atmospheric Sciences, University of British Columbia, Vancouver, British Columbia, Canada; Oregon State University

**Keywords:** Roseobacter, CHUG, population genomics, microdiversity, streamlined genomes, *Sargassum*

## Abstract

Globally dominant marine bacterioplankton lineages are often limited in metabolic versatility, owing to their extensive genome reductions, and thus cannot take advantage of transient nutrient patches. It is therefore perplexing how the nutrient-poor bulk seawater sustains the pelagic streamlined lineages, each containing numerous populations. Here, we sequenced the genomes of 33 isolates of the recently discovered CHUG lineage (~2.6 Mbp), which have some of the smallest genomes in the globally abundant Roseobacter group (commonly over 4 Mbp). These genome-reduced bacteria were isolated from a transient habitat: seawater surrounding the brown alga, Sargassum hemiphyllum. Population genomic analyses showed that: (i) these isolates, despite sharing identical 16S rRNA genes, were differentiated into several genetically isolated populations through successive speciation events; (ii) only the first speciation event led to the genetic separation of both core and accessory genomes; and (iii) populations resulting from this event are differentiated at many loci involved in carbon utilization and oxygen respiration, corroborated by BiOLOG phenotype microarray assays and oxygen uptake kinetics experiments, respectively. These differentiated traits match well with the dynamic nature of the macroalgal seawater, in which the quantity and quality of carbon sources and the concentration of oxygen likely vary spatially and temporally, though other habitats, like fresh organic aggregates, cannot be ruled out. Our study implies that transient habitats in the overall nutrient-poor ocean can shape the microdiversity and population structure of genome-reduced bacterioplankton lineages.

## INTRODUCTION

Most microbial species show fine-scale genetic diversity, known as microdiversity. Increasing evidence supports the ideas that microdiversity shapes functional traits of microbial taxa (1), promotes the temporal and spatial prevalence of microbial taxa (2, 3), stabilizes microbial communities under changing environmental conditions (4, 5), and builds a connection between microbial diversity and ecosystem functioning (6). The 16S rRNA gene amplicon sequences are often used to characterize the microdiversity of microbial populations, and those varying at the single nucleotide level (known as amplicon sequence variants [ASVs]) are shown to be ecologically meaningful (3, 7). However, an appreciable amount of genomic microdiversity has been commonly found in microbial populations whose members share identical full-length 16S rRNA genes, some of which have diversified into genetically discrete and ecologically distinct subpopulations (8, 9). Hence, analysis of 16S rRNA gene amplicon sequences may miss fine-scale microdiversity and obscure the interpretation of ecologically relevant units. Moreover, 16S rRNA genes, or any other marker genes alone, cannot provide functional information, making them of limited utility in linking microdiversity to functional traits.

The culturing of many closely related microbes, followed by genome sequencing, is a standard approach by which to study fine-scale genomic microdiversity and to pair it with phenotypic variation. Further, the genome sequences of cultured members reveal evolutionary mechanisms that structure the microdiversity. For instance, the extent of microdiversity is impacted by how niche-specifying alleles spread through microbial populations (10), which depends on the balance between recombination and selection (11). A high genome-wide recombination rate together with small selective strengths of genetic variants may induce gene-specific selective sweeps, where niche-specifying alleles spread within a population by recombination, thereby leaving the diversity of the remaining genomic regions largely unaffected (11). In contrast, a low genome-wide recombination rate along with strong selective strengths may lead to genome-wide selective sweeps, where niche-specifying alleles spread over the population by clonal expansion, thereby purging the genetic diversity throughout the whole-genome (11).

For bacteria inhabiting marine environments, the use of this population genomics approach is in its infancy. It has been largely applied to lineages with large and variable genomes, such as the *Vibrio* (9, 12, 13) and *Ruegeria* lineages (14–16), that readily grow on solid media. In sunlit pelagic oceans, however, a typical bacterioplankton cell often carries a reduced genome (with an average genome size of 2.2 to 2.6 Mbp) (17). Because of the technical difficulty and complexity in culturing genome-reduced bacterioplankton members, population genomic analyses are rare. A few studies have instead turned to single-cell amplified genomes (SAGs) (18). Despite the appreciable insights achieved, SAGs are limited in population genomic applications, owing to their low completeness and high error rates (19), which disable the inference of gene gain and loss events and hinder the interpretation of nucleotide substitution patterns, respectively, when nearly identical genomic sequences are compared (20). Further, SAGs do not allow for the pairing of physiological assays and genetic manipulations with population genomic analyses.

Here, we isolated 33 strains affiliated with the recently discovered CHUG cluster, a genome-reduced lineage (~2.6 Mbp) (21) of the globally abundant marine Roseobacter group, which typically contains copiotrophic members with large and variable genomes (>4 Mbp on average) that are commonly associated with phytoplankton groups in the pelagic ocean (22, 23). While CHUG genomes are larger than those of the most abundant marine bacterioplankton lineages, such as the alphaproteobacterial SAR11 clade (1.3 to 1.4 Mbp) and the cyanobacterial *Prochlorococcus* (1.6 to 1.8 Mbp), they are, nevertheless, among the smallest of the Roseobacter genomes. The CHUG members differ by up to 1.8% in their 16S rRNA gene sequences, and they together differ from their closest sister group (3.9 Mbp on average) by 3.5% in the same gene (21). An important consequence of genome reduction is that CHUG members have lost the ability of de novo synthesis of vitamin B_12_ (21). This is unusual because the potential for vitamin B_12_ synthesis is widespread among Roseobacter members (21, 22) and sets the ground for mutualistic interactions between Roseobacter members and phytoplankton groups that are commonly auxotrophic for vitamin B_12_ (24–26), suggesting that phytoplankton may not be an important pelagic niche for CHUG (21). In the present study, we chose the ambient seawater of the brown algae *Sargassum hemiphyllum* as our study site because two of the eight published CHUG genomes (21) were isolated from the same site and because they show evidence of metabolic interaction with brown algae by utilizing L-fucose, the dominant structural monosaccharide composing the polysaccharide fucoidan present in the cell walls of brown algae, as their sole carbon source. The 33 CHUG isolates share (nearly) identical 16S rRNA genes, thereby providing a unique opportunity to explore mechanisms that shape the microdiversity of an important, genome-reduced, marine bacterioplankton lineage.

## RESULTS AND DISCUSSION

### Phylogenetic and genetic structures indicate sequential speciation events.

The 33 CHUG isolates (Data Set S1a) share identical 16S rRNA gene sequences, except that HKCCA1312 and HKCCA1076 show 1 to 2 nucleotide differences from the remaining isolates, and all of them show a whole-genome average nucleotide identity (ANI) above 95.8% (Fig. S1). Both statistics are well beyond the thresholds widely used to define bacterial species: 98.7% (alternatively, 99.0% and 99.5%) at the 16S rRNA gene level (30) and 95% at the ANI level (31), suggesting that the 33 isolates fall into a single “species”, according to the operational definition of the term.

10.1128/mbio.00571-22.3 FIG S1 The heat map of the pairwise identity of 16S rRNA genes and the whole-genome average nucleotide identity (ANI) of the 33 CHUG isolates. Genomes are arranged according to the phylogenomic tree shown on the left. Download FIG S1, PDF file, 1.2 MB. Copyright © 2022 Chu et al. 2022 Chu et al. https://creativecommons.org/licenses/by/4.0/ This content is distributed under the terms of the Creative Commons Attribution 4.0 International license.

10.1128/mbio.00571-22.9 Data Set S1 (a) Genome assembly summary of all isolates. The completeness, contamination, and heterogeneity were estimated with CheckM, and the remaining statistics were calculated with QUAST. Insertion sequences (IS), prophages, genomic islands (GIs), and pseudogenes were identified using the ISfinder web server, PHASTER web server, IslandViewer4 server, and a modified procedure of Psi-Phi, respectively. (b) Statistics of *d_S_* values between M1M2 versus M3M4M5 and those within either M1M2 or M3M4M5 for the two clusters across 1,783 shared, single-copy gene families identified with the k-means clustering algorithm. The optimal number (k = 2) of the clusters was determined with the R package, “NbClust”. (c) Summary of the 38 *d_S_* outlier gene families subjected to novel allelic replacements, including gene name, functional annotation, and the split event(s) to which they contributed. (d) Functional annotation of the 210 M1M2-specific gene families. For those potentially involved in carbon source utilization, their substrates are predicted. Genes are represented by loci from strain HKCCA1288. (e) Functional annotation of the 115 M3M4M5-specific gene families. For those potentially involved in carbon source utilization, their substrates are predicted. Genes are represented by loci from strain HKCCA1061. (f) Functional annotation of the 41 M3M4-specific gene families. For those potentially involved in carbohydrate utilization, their substrates are predicted. Genes are represented by loci from strain HKCCA1006. (g) Functional annotation of the 15 M5-specific gene families. For those potentially involved in carbohydrate utilization, their substrates are predicted. Genes are represented by loci from strain HKCCA1063. (h) Functional annotation of the 57 M5S1-specific gene families. For those potentially involved in carbohydrate utilization, their substrates are predicted. Genes are represented by loci from strain HKCCA1063. (i) Functional annotation of the 28 M5S2-specific gene families. For those potentially involved in carbohydrate utilization, their substrates are predicted. Genes are represented by loci from strain HKCCA1066. (j). Functional genes involved in carbohydrate utilization that are differentiated between M1M2 and M3M4M5. Genes are represented preferentially by the locus ID of the genome-closed HKCCA1288 and, alternatively, by that of HKCCA1061. The “*d_S_* outlier” gene families are shared by all members of M1M2 and M3M4M5, but they were subjected to novel allelic replacement. The “M1M2-specific” and “M3M4M5-specific” gene families are only present in M1M2 and M3M4M5, respectively. The gene families linked to “different Roary gene families with the same COG annotation” each consist of two families identified by Roary, but they are annotated with the same function. (k) Statistics of oxygen uptake rate measurements for four CHUG representative strains and Ruegeria pomeroyi DSS-3. Download Data Set S1, XLSX file, 0.2 MB. Copyright © 2022 Chu et al. 2022 Chu et al. https://creativecommons.org/licenses/by/4.0/ This content is distributed under the terms of the Creative Commons Attribution 4.0 International license.

**FIG 1:**
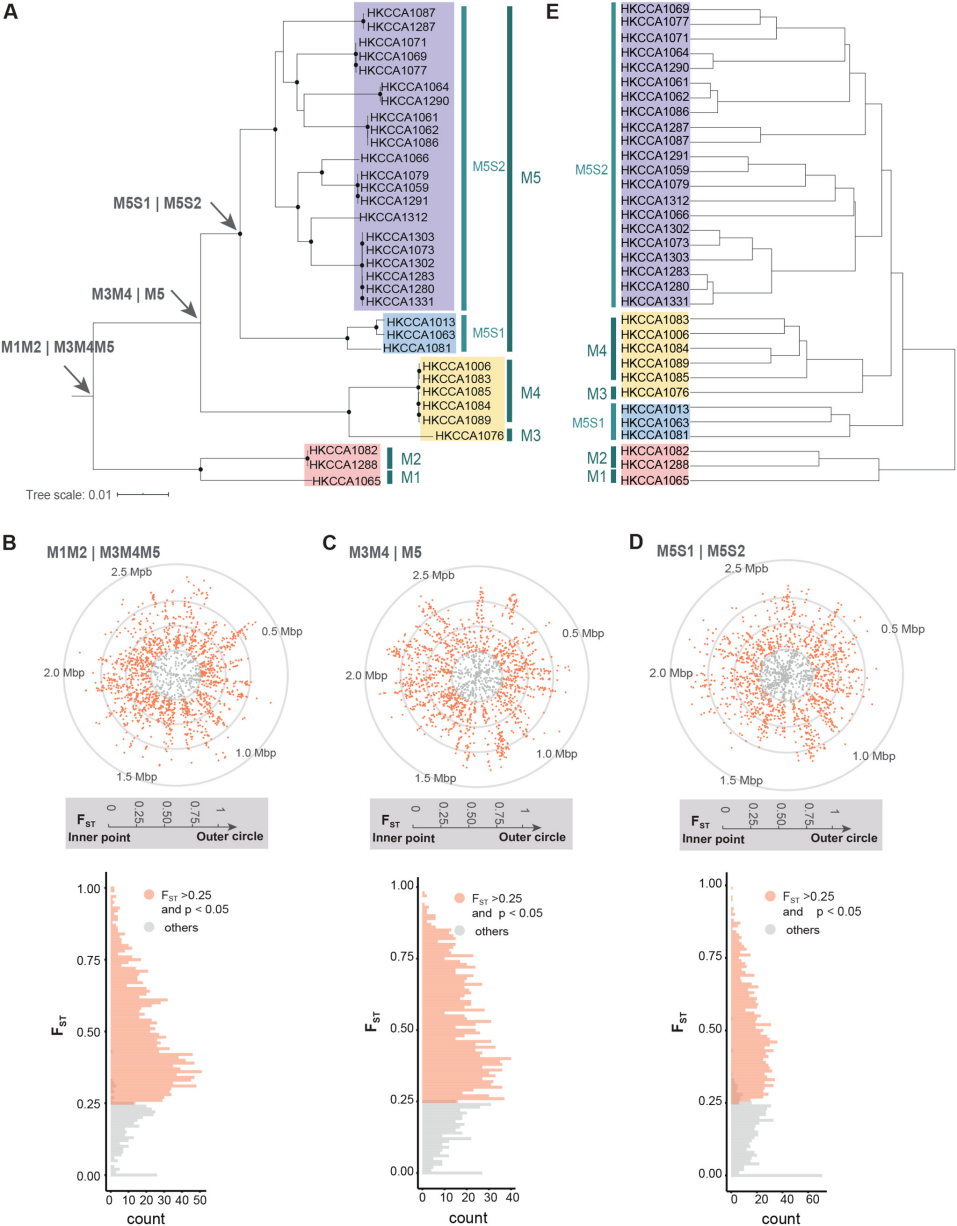
Population differentiation of the 33 CHUG isolates from a single 50 mL seawater sample surrounding a brown alga. (A) Genetically isolated populations from M1 to M5 as well as the two subpopulations, M5S1 and M5S2, within M5 that were defined by PopCOGenT are mapped to the maximum likelihood phylogenomic tree constructed by IQ-TREE, with the root position determined by the MAD algorithm. Solid circles in the phylogeny indicate nodes with bootstrap values of 100% in the 1,000 bootstrapped replicates. Three split events discussed in the main paper are marked with arrows. (B to D) The distribution of the F_ST_ values across 1,788 core genes between the lineages, resulting from each of the three split events. Gene families showing F_ST_ > 0.25 with statistical significance (*P* < 0.05) are marked in red, and others are marked in gray. For the dot plots (top), genes are ordered along the closed genome of HKCCA1288. For the histogram (bottom), the count represents the number of gene families. (E) The dendrogram is constructed based on gene presence and absence using the complete linkage method implemented in the R package, “*pheatmap*”.

The clonal evolutionary history, reconstructed from genomic regions free from recombination, showed that the 33 isolates have diverged into multiple phylogenetic clusters (Fig. 1A). PopCOGenT divided the 33 isolates into five genetically isolated populations (M1, M2, M3, M4, and M5) (Fig. 1A) and further divided M5 into two subpopulations (M5S1 and M5S2), between which genetic isolation has not been completed. Integrating the phylogenetic and genetic structures of these CHUG members, we inferred that the earliest speciation event occurred between M1M2 and M3M4M5, and follow-on events include the M3M4-M5 split and the M1-M2 split among others. Previous studies were focused on how a single speciation event shapes the pattern of genomic microdiversity (8, 9, 32, 33), yet the effect of multiple successive speciation events on genomic microdiversity has rarely been investigated. Therefore, the population-level data set of CHUG not only allows learning some new strategies that a representative, genome-reduced Roseobacter lineage uses for pelagic niche adaptation but also provides an opportunity to link complex, extant genomic microdiversity to a series of past speciation events and disentangle how each speciation event contributed to the collective genomic microdiversity. It is worth noting that “speciation” refers to a process of genetic separation, and this term is commonly used in studies of microbial population differentiation within an operationally defined species (32, 34, 35).

Genetic variations can be sourced from recombination and mutation, whose relative frequencies (ρ/θ) were estimated by ClonalFrameML (36). The entire CHUG population, comprised of 33 isolates, features a low recombination rate (ρ/θ = 0.079), indicating either a reduced gene flow between the genetically isolated populations defined by PopCOGenT or that these members are inherently clonal (i.e., recombination rarely occurs). To test these competing hypotheses, we turned to the subpopulation M5S2, which is comprised of 21 members, displays a large amount of genetic diversity, and is panmictic by definition. The ρ/θ ratio of M5S2 (0.067) is comparable to that of the entire CHUG population, suggesting that clonality, rather than population differentiation, is the primary explanation of the low ρ/θ ratio. A similar pattern was shown for the relative effect (r/m) of recombination and mutation (1.690 for the entire population and 1.784 for M5S2). Since ClonalFrameML identifies recombination events based on the clustered distribution of single nucleotide variants, which are primarily introduced by recombination with external lineages (i.e., those evolutionarily separated from the members under study) (36, 37), the appreciable difference between ρ/θ and r/m shown here suggests that such recombination plays an important role in the genetic differentiation of the sampled CHUG lineage.

At first glance, the results from ClonalFrameML and PopCOGenT appear to be contradictory. On one hand, both the recombination rate (ρ/θ) and relative effect (r/m), as measured by ClonalFrameML, are largely invariable between and within the populations defined by PopCOGenT. On the other hand, recombination must be more frequent within than between populations, as this is the criterion used by PopCOGenT to delineate population boundaries. To this end, it is worth emphasizing that PopCOGenT exclusively counts recombination events occurring within the studied lineage because it detects gene flow based on the enrichment of identical genomic regions, whereas ClonalFrameML mainly focuses on recombination with external lineages. Therefore, the analyses by ClonalFrameML and PopCOGenT are complementary.

### The earliest speciation event has the most profound impacts on genomic microdiversity.

We provide three lines of evidence to support the hypothesis that the first split, separating M1M2 from M3M4M5, left the strongest signatures in the genomes. F_ST_ is a proxy for the levels of allelic fixation and genetic differentiation in core genomes (38, 39), and the core DNA is considered to have reached a high level of differentiation if the F_ST_ value is statistically significant (*P* < 0.05) and is greater than 0.25 (40, 41). According to this criterion, 82.9% of the 1,788 single-copy core genes shared by M1M2 and M3M4M5 (Fig. 1B) reach a high level of differentiation, which is significantly greater (two-proportion *z*-test, *P* < 0.001) than that following the split of M3M4 from M5 (78.8%) (Fig. 1C) and that of the ongoing split of M5S1 and M5S2 (66.8%) (Fig. 1D). F_ST_ cannot be calculated for other splits, including the genetic separation of M1 from M2 or that of M3 from M4, because one of the populations under comparison (M1 in the former comparison and M3 in the latter) is comprised of only a single member.

An important evolutionary mechanism driving population differentiation in core genomic regions is novel allelic replacements via homologous recombination with distant relatives. Such replacements are expected to leave genetic signatures at synonymous sites in protein-coding genes, which manifest as unusually large synonymous substitution rates (*d_S_*) at the recombined loci compared to those of the remaining genes (42). The underlying principle is that selective forces on nucleotide substitutions at synonymous sites are weak. Thus, unusually high *d_S_*, resulting from replacements via homologous recombination with divergent lineages, in a given gene are unlikely to be overwritten by changes induced by other processes, such as mutation and selection. In the present study, this approach is used to compare the roles of sequential speciation events in shaping the genomic diversity of the 33 CHUG members. In total, we identified 38 core gene families showing unusually large *d_S_* values (see Text S1.6 for more details; Data set S1b and Data set S1c). By integrating gene tree topological structures and the pairwise *d_S_* values among gene members within a family, we were able to infer at which phylogenetic branches the divergent alleles were introduced to the population. For example, 25 of the 38 gene families each consistently show unusually large *d_S_* values in pairwise comparisons between M1M2 and M3M4M5 but generally small *d_S_* values when compared to members within either M1M2 or M3M4M5, indicating that allelic replacements occurred at the last common ancestor (LCA) of either M1M2 or M3M4M5 (Fig. S2). Likewise, we inferred that five gene families were replaced at the LCA of either M3M4 or M5, and seven gene families were replaced at the LCA of either M5S1 or M5S2 (Fig. S2). Note that 15 gene families were subjected to multiple allelic replacements at different evolutionary branches and may have contributed to several speciation events (Fig. S2). Therefore, this analysis provides new evidence in support of the idea that the earliest split event left the strongest imprints on the diversity of the core genome.

10.1128/mbio.00571-22.1 TEXT S1 Supplemental methods. Download Text S1, DOCX file, 0.1 MB. Copyright © 2022 Chu et al. 2022 Chu et al. https://creativecommons.org/licenses/by/4.0/ This content is distributed under the terms of the Creative Commons Attribution 4.0 International license.

10.1128/mbio.00571-22.4 FIG S2 Allelic replacements of 25 outlier gene families that support the divergence between M1M2 and M3M4M5. For each gene family, the phylogenomic tree is displayed on the left, where arrows represent novel allelic replacement. Dark arrows show the novel allelic replacements by recombination with external lineages that are phylogenetically separated from the 33 CHUG isolates. Dotted arrows denote the recombination between populations. On the right is a gene tree, where different populations are marked with different colors. These gene families are classified as (A) carbohydrate metabolism, (B) membrane transport, (C) respiration, (D) nucleosides and nucleotides metabolism, (E) amino acids and derivatives metabolism, (F) DNA metabolism, (G) RNA metabolism, (H) metabolism of aromatic compounds, (I) cell division and cell cycle, and (J) unclassified protein. Download FIG S2, PDF file, 0.2 MB. Copyright © 2022 Chu et al. 2022 Chu et al. https://creativecommons.org/licenses/by/4.0/ This content is distributed under the terms of the Creative Commons Attribution 4.0 International license.

**FIG 2:**
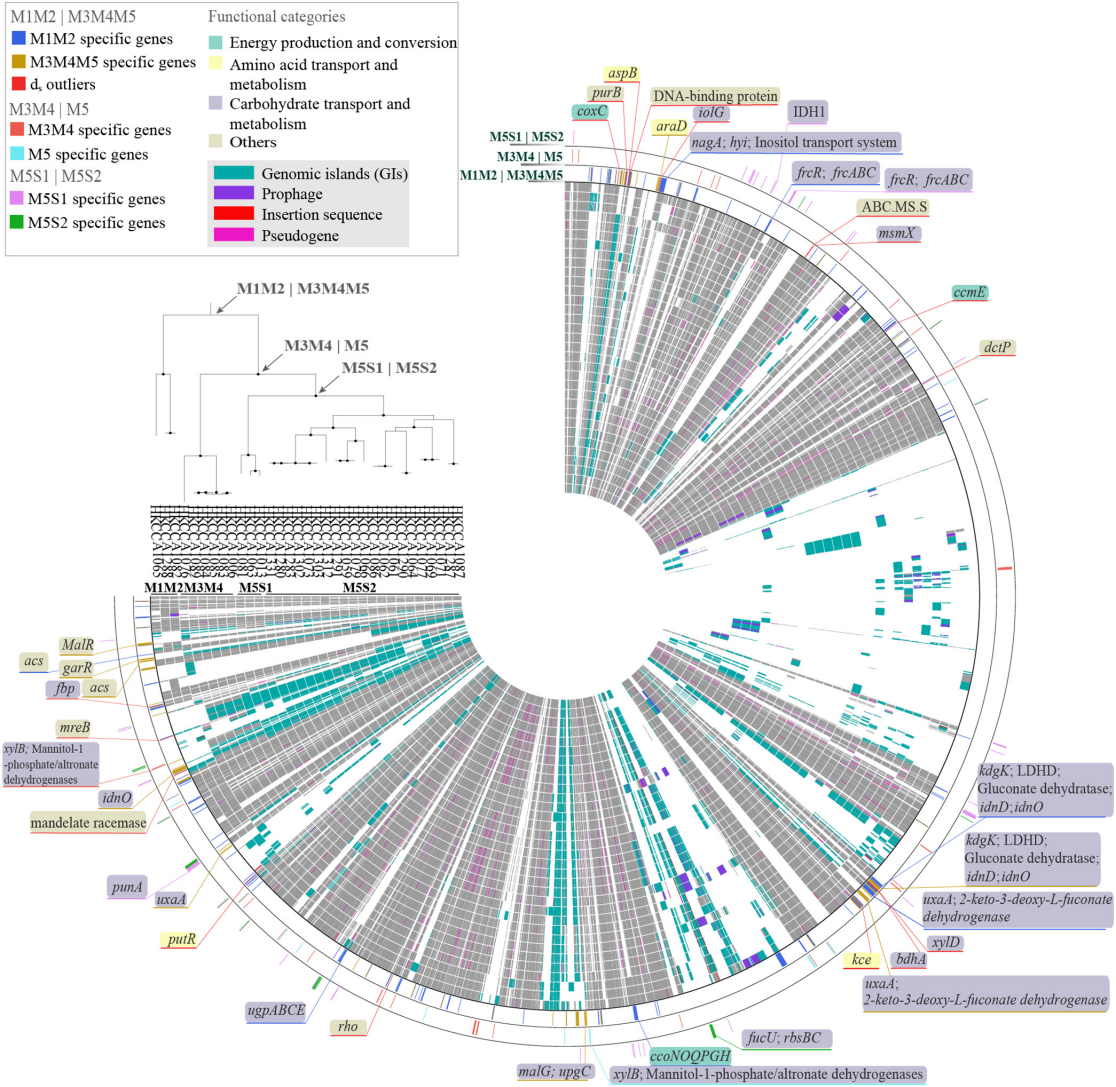
The pangenome of the 33 CHUG isolates. All of the orthologous gene families identified by Roary are positioned according to the closed genome, HKCCA1288. From the inner to the outer circle: (1 to 33) the genomes of the 33 CHUG isolates, arranged in line with their phylogenomic tree, which is shown in the top-left corner. Genomic islands, prophages, insertion sequences, and pseudogenes are marked with different colors and are mapped to each genome; (34) M1M2-specific genes, M3M4M5-specific genes, and core genes showing unusually large *d_S_* values are marked with different colors and are placed according to the coordinates of the HKCCA1288 genome; (35) M3M4-specific genes and M5-specific genes; (36) M5S1-specific genes and M5S2-specific genes. Gene families falling in the functional categories, “energy production and conversion”, “amino acid transport and metabolism”, and “carbohydrate transport and metabolism”, are each attached with a gene name and framed in a box with a background color corresponding to a functional category. Each box is connected with a line that is colored according to whether the gene family is part of the population-specific accessory genes or the *d_S_* outlier core genes.

Further evidence comes from the accessory genome. Consistent with the phylogenomic tree, the topology of the gene content dendrogram, constructed based on gene presence and absence in the 33 CHUG genomes (Fig. 1E), supports the separation of M1M2 from M3M4M5. Within M3M4M5, however, the dendrogram shows that the subpopulation M5S2 clusters with M3M4 instead of the subpopulation M5S1, suggesting that genetic separation within M3M4M5 has not been completed at the accessory genome. Therefore, this analysis supports the claim that the genomic diversity of the 33 CHUG members was most profoundly impacted by the earliest split event. Over 60% (2,108 out of 3,451) of the accessory gene families are mapped to genomic islands (GI), and a dendrogram based on these GI-associated genes still supports both the split between M1M2 and M3M4M5 and a mixed relationship within M3M4M5 (Fig. S3). This suggests that GIs may contribute appreciably to the population differentiation of these CHUG populations. Pseudogenes and mobile genetic elements other than GIs, such as insertion sequences and prophages, are also abundant (Data set S1a; Fig. 2), suggesting that they are also important drivers of the genomic heterogeneity of the sampled CHUG members. An excess of pseudogenes, GIs, and other mobile elements makes CHUG unique compared to most known marine bacterioplankton lineages with reduced genomes, in which these elements are depleted (43–45).

10.1128/mbio.00571-22.5 FIG S3 The dendrogram of the 33 CHUG isolates based on the presence and absence of the genes located at the genomic islands. Download FIG S3, PDF file, 0.9 MB. Copyright © 2022 Chu et al. 2022 Chu et al. https://creativecommons.org/licenses/by/4.0/ This content is distributed under the terms of the Creative Commons Attribution 4.0 International license.

### Differential utilization of carbon sources correlates with the earliest speciation event.

Since population-specific gene families (exclusively and ubiquitously found in one population but absent in another) may provide clues regarding functional differentiation, we focused on the 210 M1M2-specific and 115 M3M4M5-specific gene families defined by Roary (Data Set S1d and e), which include 152 and 91 families, respectively, that can be mapped to Clusters of Orthologous Groups (COG) families with known functions. Of these, 33 M1M2-specific and 23 M3M4M5-specific families defined by Roary are potentially involved in the utilization of a variety of carbon sources (mostly carbohydrates), suggesting that the availability of distinct carbon sources and the differential utilization of these carbon sources may have contributed to the earliest speciation event. It is worth mentioning that the differential utilization of carbohydrates may also contribute to follow-on speciation events, since carbohydrate utilization genes are part of the M3M4-specific (2 out of 23), M5-specific (2 out of 4), M5S1-specific (6 out of 32), and M5S2-specific (7 out of 21) gene families (Fig. 2; Data Set S1f–i).

We predicted 20 carbon sources that show genetic differentiation between M1M2 and M3M4M5 (Fig. 2; Table 1; Text S2.1). To systematically characterize the phenotypic differentiation involved in organic substrate utilization, we employed BiOLOG phenotype microarrays, which consist of 190 distinct carbon sources. There are two benefits of using this high-throughput technique. First, it allows testing the substrates underlying the genetic differentiation, since it includes 16 of the 20 above-mentioned carbon sources (Table 1). Second, it allows testing additional substrates that are not predicted by a bioinformatic analysis. One caveat of using this technique is that BiOLOG plates contain all carbon substrates at the same concentration, but bacterial responses may depend on the concentrations of the carbon compounds. That is, negative BiOLOG results may turn positive in a concentration different from that under BiOLOG and vice versa.

**TABLE 1:** Organic compounds that support genetic or phenotypic differentiation between M1M2 and M3M4M5^*a*^

Compounds	M1M2-specific^*b*^	M3M4M5-specific^*b*^	*d_S_* outliers^*c*^	Different Roary families with same COG annotation^*d*^	BiOLOG assay	Macroalgae-derived
Hydroxypyruvate	Yes				-	
N-acetylglucosamine	Yes				No growth	
D-fructose	Yes				Differential growth^*e*^	Brown, red, and green (51)
Myo-inositol	Yes		Yes		No growth	Brown, red, and green (51)
Glycerol-3-phosphate	Yes			Yes	No growth	Red (89)
D-arabinose		Yes			Equal growth	
Maltose		Yes			No growth	Green (90)
D-glycerate		Yes			-	
Tartronate semialdehyde		Yes			-	
β-hydroxybutyric acid			Yes		Differential growth	Brown (46)
D-xylose			Yes		Equal growth	Brown, red, and green (50)
D-galacturonate				Yes	Equal growth	
D-glucuronate				Yes	No growth except one replicate of the M2 representative	Brown (52)
Methyl D-Lactate				Yes	No growth except one replicate of the M2 representative	
Pyruvate				Yes	Equal growth	
D-gluconate				Yes	No growth	
Ketogluconate				Yes	Equal growth	
2-keto-3-deoxy-L-fuconate				Yes	-	
L-fucose				Yes	Differential growth	Brown ([Bibr B27][Bibr B28] 29)
Acetic acid				Yes	Differential growth	Red (47)
Glycolic acid					Differential growth	Brown (49)
Sodium formate					Differential growth	
Mono-methyl succinate					Differential growth	
D-arabitol					Differential growth	
D-fucose					Differential growth	
γ-hydroxybutyric Acid					Differential growth	

a Compounds potentially made by macroalgae are marked in the last column.

b The “M1M2-specific” and “M3M4M5-specific” gene families are exclusively present in M1M2 and M3M4M5, respectively.

c The “*d_S_* outlier” gene families are shared by all members of M1M2 and M3M4M5 but were subjected to novel allelic replacement.

d Gene families linked to “different Roary gene families with the same COG annotation” each consist of two families identified by Roary but are annotated with the same function. The gene locus, COG id, gene names, and functions of the involved gene families can be found in Data Set S1j.

e The differential growth is between M1 and the remaining populations. In other cases, differential growth was observed between M1M2 and M3M4M5.

10.1128/mbio.00571-22.2 TEXT S2 Supplemental results. Download Text S2, DOCX file, 0.03 MB. Copyright © 2022 Chu et al. 2022 Chu et al. https://creativecommons.org/licenses/by/4.0/ This content is distributed under the terms of the Creative Commons Attribution 4.0 International license.

**FIG 3:**
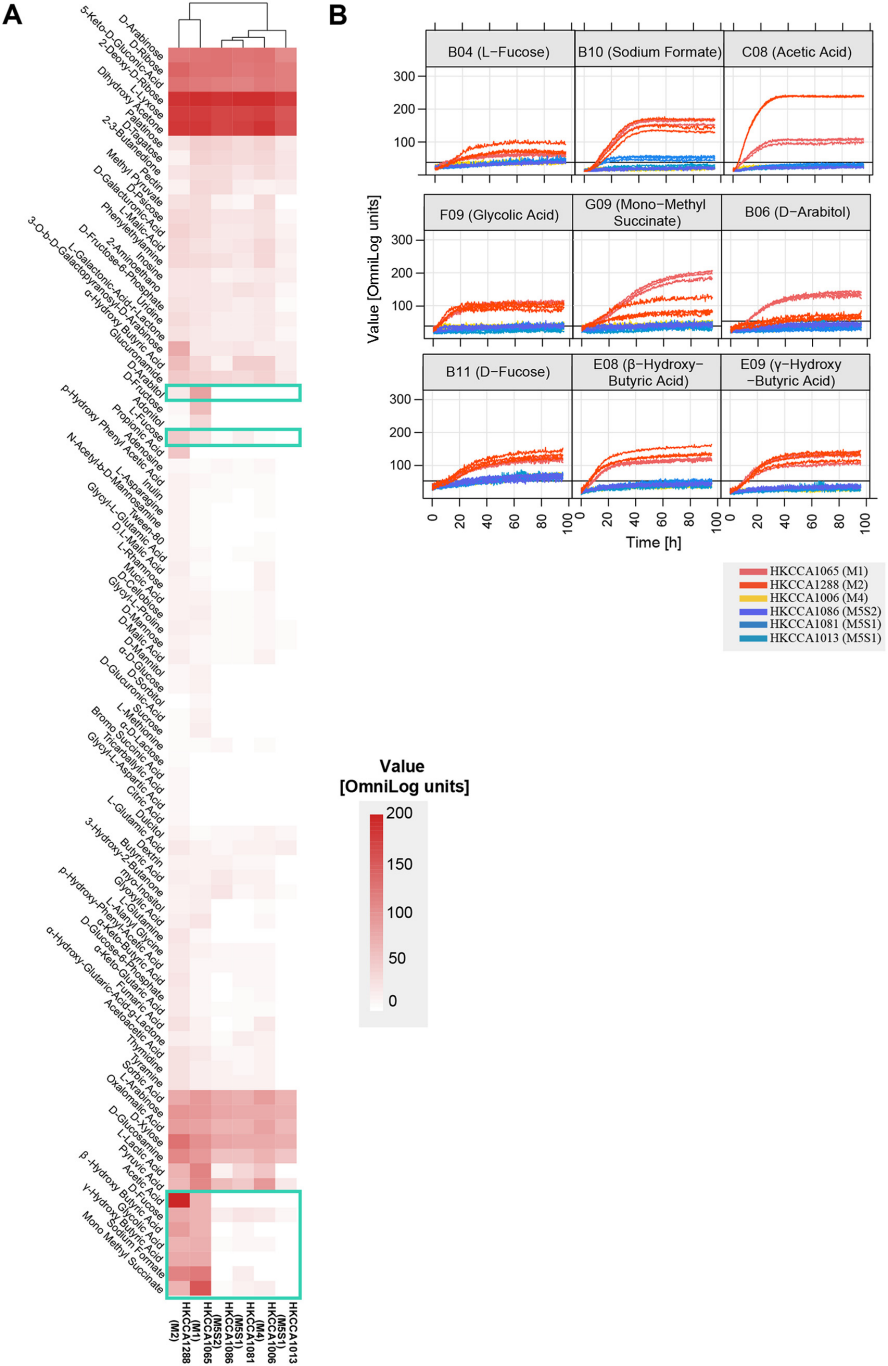
(A) The heat map of the respiration values of a few representative strains of the populations defined by PopCOGenT at 96 h, measured by BiOLOG. The clustering is constructed using all 190 carbon sources provided by the two microplates, PM01 and PM02, but only those 85 substrates that showed a respiration value over 10 OmniLog units in at least one strain are displayed. Substrates differentially utilized by M1M2 and M3M4M5 are framed with green boxes. (B) The respiration curves of the representative strains fed with the framed substrates in (A).

Of the 16 carbon sources mentioned above, 9 are potentially made by macroalgae (Table 1). Three macroalgae-derived compounds (β-hydroxybutyric [46], L-fucose [27–29], and acetic acid [47]) each were differentially utilized by M1M2 and M3M4M5 (Fig. 3; Table 1), which is correlated with the genetic differentiation of the corresponding gene families (Data set S1j; Text S2.1). Of these compounds, L-fucose is a major component of macroalgal fucoidan and constitutes up to 40% of the monosaccharides in some brown algae (27). Acetic acid can be released by some red algae (47), though it is commonly produced by other marine organisms, including bacteria (48). Moreover, M1M2 showed better growth than did M3M4M5 when utilizing another six different carbon sources in the phenotype microarray assay (Fig. 3B), including glycolic acid, which is a major component in brown algae (49). While gene differentiation between M1M2 and M3M4M5 was not detected for these six compounds, genetic divergence at intergenic regions for regulation cannot be ruled out. It is also worthy of note that 11 other carbon sources led to comparable respiratory intensity in M1M2 and M3M4M5. Some of these carbon sources may be derived from macroalgae, such as d-xylose (brown, red, and green macroalgae) (50), d-ribose (brown and green macroalgae) (51), and d-arabinose (brown macroalgae) (51, 52). Our results suggest that while M1M2 and M3M4M5 are each optimized for utilizing a specific group of carbon sources, the sampled CHUG members might be, in general, well-prepared to make use of the resources available in macroalgal ecosystems (Fig. 3; Fig. S4 and S5).

10.1128/mbio.00571-22.6 FIG S4 The utilization of 95 carbon sources provided by BiOLOG microplate PM01 by the six representative isolates from different populations. Three replicates were performed for each strain. Download FIG S4, PDF file, 2.7 MB. Copyright © 2022 Chu et al. 2022 Chu et al. https://creativecommons.org/licenses/by/4.0/ This content is distributed under the terms of the Creative Commons Attribution 4.0 International license.

10.1128/mbio.00571-22.7 FIG S5 The utilization of 95 carbon sources provided by BiOLOG microplate PM02 by the six representative isolates from different populations. Three replicates were performed for each strain. Download FIG S5, PDF file, 2.6 MB. Copyright © 2022 Chu et al. 2022 Chu et al. https://creativecommons.org/licenses/by/4.0/ This content is distributed under the terms of the Creative Commons Attribution 4.0 International license.

The divergences and coherences between M1M2 and M3M4M5 members at the phenotypic level were further analyzed by clustering the tested strains based on respiration levels as a proxy for growth responses to carbon source availability. The resulting dendrogram (Fig. 3A) shows that the M1M2 members are well-separated from the M3M4M5 members, whereas clades within M3M4M5 are mixed. This evidence further supports the idea that the earliest speciation event, that is, the event splitting M1M2 and M3M4M5, has shaped the physiological diversity in the 33 CHUG members, whereas the follow-on speciation events have made a limited contribution.

### Differential adaptation to low and high oxygen niches is another key driver of the earliest speciation event.

Another notable observation is that while all CHUG members possess the cytochrome aa_3_ oxidase (*ctaBCDEG*), which is known to have a low oxygen (O_2_) affinity and thereby enables them to respire under high O_2_ conditions (53), all M1M2 members additionally and exclusively harbor the cytochrome cbb_3_ oxidase (*ccoGHNOPQ*) (Fig. 2), which is known to exhibit a high O_2_ affinity that may function under microaerobic conditions (54–56). Moreover, a regulatory gene, *FnrL*, is located upstream of the *ccoGHNOPQ* operon, which may regulate cbb_3_ oxidase gene expression (57, 58) when M1M2 members switch from aerobic to microaerobic niches.

**FIG 4:**
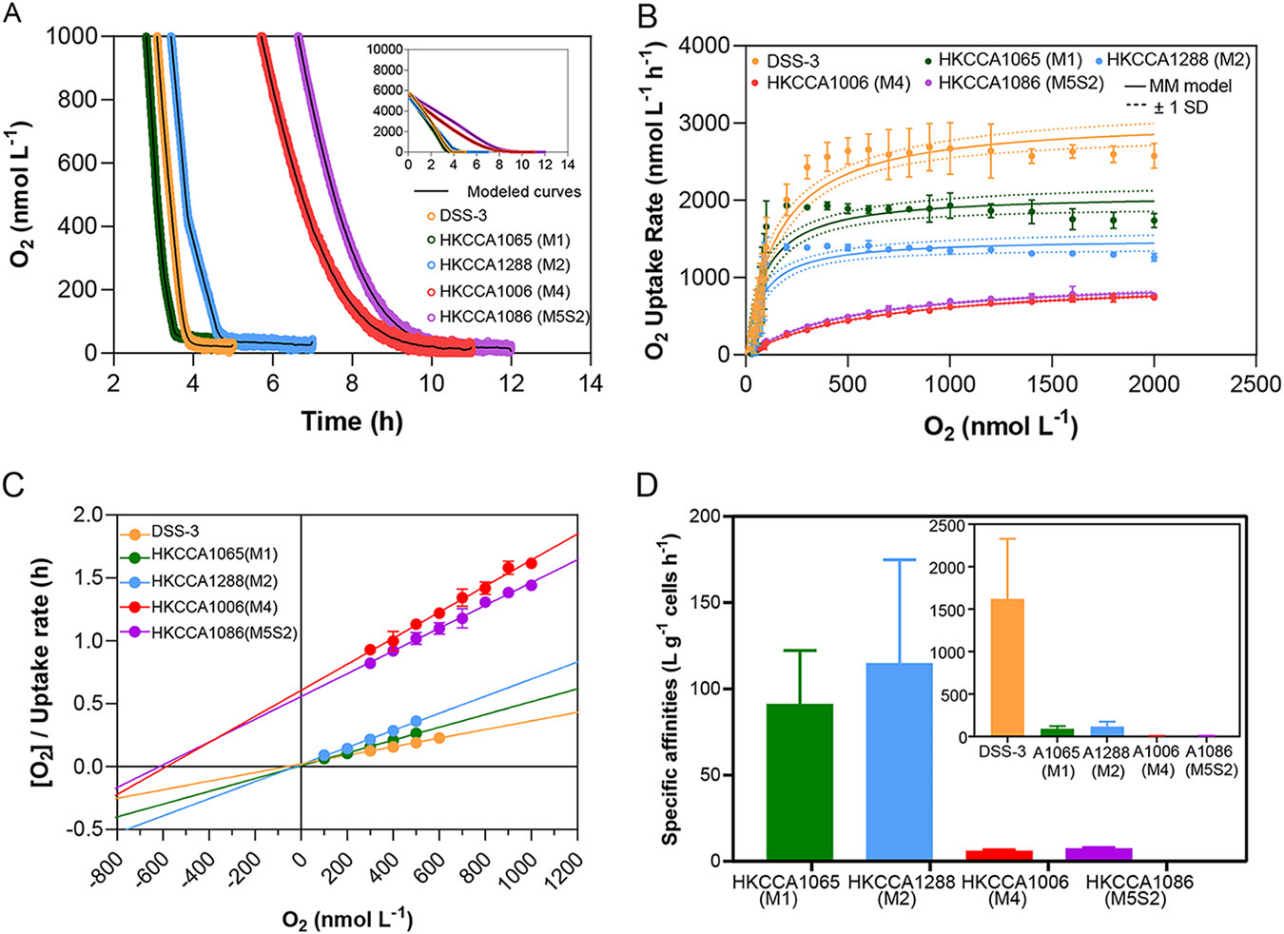
Oxygen uptake measurements. (A) Oxygen consumption of two CHUG isolates (HKCCA1288 and HKCCA1065) from M1M2, two CHUG isolates (HKCCA1006 and HKCCA1086) from M3M4M5, and Ruegeria pomeroyi DSS-3 under microaerobiosis. (B) The relationship between oxygen concentration and oxygen uptake rates at an oxygen level of <2 μmol/L. The solid line is a model fitted based on the Michaelis-Menten equation, and the nearby dotted line shows the standard deviation estimated by this equation. The error bars of the filled circles represent the standard deviations of three replicates. (C) The Hanes-Woolf plot of oxygen uptake as a function of oxygen concentration, ranging from 100 nmol/L to 1 μmol/L. The error bars of the filled circles represent the standard deviations of three replicates. (D) The estimated specific affinity of oxygen for the four CHUG isolates and R. pomeroyi DSS-3.

Our O_2_ uptake experiments revealed that, under microaerobic conditions, O_2_ was consumed slower in the M3M4M5 cultures than in both the M1M2 cultures and in Ruegeria pomeroyi DSS-3 (Fig. 4A), a model Roseobacter with a large genome (4.6 Mbp) that harbors both the aa_3_ oxidase and the cbb_3_ oxidase (59). Kinetic parameters, including the maximum respiration rate (V_max_) and the half-saturation constants (apparent K_m_ values) are used to describe the respiratory rates as a function of O_2_ concentration using a Michaelis-Menten model (Fig. 4B and C). Estimated K_m_ values for M1M2 and R. pomeroyi DSS-3 were found to be lower than those of M3M4M5 (Data Set S1k), which is consistent with the fact that only M1M2 and R. pomeroyi DSS-3 possess the cbb_3_ oxidase with a high O_2_ affinity. Next, the specific affinity for O_2_, which quantifies the ability of bacteria to take up O_2_ at low concentrations (60, 61), shows an order-of-magnitude difference between M1M2 and M3M4M5 (Fig. 4D; Data Set S1k), further indicating their functional divergence through microaerobiosis.

Macroalgae release adequate bioavailable DOCs to enable the proliferation of ambient heterotrophic microorganisms (62–64), which subsequently deoxygenate the ambient waters at a microspatial scale (65). Moreover, the DOC release process is dynamic so that the spatial and temporal depletion of O_2_ may exist in macroalgal ecosystems (64, 66), and this may contribute to the formation of the two main ecotypes of CHUG: M1M2 and M3M4M5. A further observation is that R. pomeroyi DSS-3 shows a much larger specific affinity than does CHUG, which implies its greater potential to acquire O_2_ from exceedingly low O_2_ habitats (Fig. 4D; Data set S1k). This appears to be an adaptive strategy of this Roseobacter; since it is often associated with organic particles in the water column and in the interior of the particles, O_2_ may be depleted owing to aerobic degradation at the outer part of the particles (67, 68).

### Concluding remarks and caveats.

CHUG is a unique Roseobacter lineage that differs from all other pelagic Roseobacter members in that its members have some of the smallest genomes in the Roseobacter group, their global distribution is not correlated with phytoplankton abundance, they lack the ability to de novo synthesize vitamin B_12_, and they cannot grow on many carbon compounds made by phytoplankton (21). The decoupling from the common habitat in the pelagic ocean, namely, phytoplankton, shared by most pelagic Roseobacter members, prompted us to explore potential habitats for CHUG. Here, we provided evidence that the speciation of the CHUG members correlates with the differential utilization of low-molecular-weight labile organic compounds and the differential ability to explore low-oxygen niches. Since these resources and conditions are transient in the water column, our findings suggest that CHUG may explore ephemeral habitats where nutrient concentrations may be occasionally high but oxygen levels may be transiently low. The available data are consistent with the idea that brown algae are likely an important habitat of the CHUG members, since they were sampled from the ambient seawater of the brown algae *Sargassum* and since they can grow on L-fucose, the dominant carbohydrate found in brown algae, as the sole carbon source, whereas all other pelagic Roseobacter lineages (21), such as DC5-80-3 (also called “RCA”), CHAB-I-5, and NAC11-7, lack the key gene (*fucA*) that enables the use of this compound. Nevertheless, other possible habitats cannot be ruled out. For example, fresh organic aggregates may also enrich nutrients and deplete oxygen in their ambient water immediately surrounding the particles, and there is no compelling reason that L-fucose cannot be an important component in such habitats. Given the seasonal occurrence of *Sargassum*, future researchers may perform longitudinal sampling and combine culture-based approaches with metagenomics and metatranscriptomics to validate brown algae as an important habitat for CHUG.

## MATERIALS AND METHODS

The 33 CHUG isolates were obtained from a single 50 mL sample of water surrounding brown alga *Sargassum hemiphyllum* at Lobster Bay (22.309° N, 114.3015° E), Hong Kong SAR, China, on 15 Mar 2017. All CHUG members were isolated using a modified marine basal medium (MBM) (14) and cultivated with 2216 marine agar (BD Difco, USA). The 33 CHUG genomes were sequenced using the BGISEQ-500 platform in PE100 sequencing mode. Genomes were assembled using SPAdes v3.9.1 (69) with high quality reads, and the quality of assemblies was determined with CheckM v1.0.7 (Data set S1a) (70). Genes were predicted with Prokka v1.11 (71) and annotated with the RAST server v2.0 (72), the CDD database v3.16 (73), the KEGG database v82.0 (74, 75), and the COG database (76). Genomic islands, prophages, insertion sequences (IS), and pseudogenes were identified using the online IslandViewer 4 server (77), PHASTER web server (78), ISfinder web server (79), and a modified procedure of Psi-Phi (14, 80), respectively.

To construct the clonal evolutionary history of the CHUG members, a maximum likelihood (ML) phylogenomic tree was constructed using IQ-TREE v1.6.5 (81) with a shared genomic DNA alignment, in which all recombined sites were masked by Gubbins 2.4.1 (82). Note that the shared genomic DNA alignment was derived from a whole-genome alignment and includes both protein-coding genes and noncoding genomic regions. To root the tree, we initially tried the outgroup-based method. However, the most closely related sister lineage among all available genome-sequenced lineages is connected with the CHUG lineage through a long branch (Fig. S6A), suggesting that it is not an appropriate outgroup and that using it to root the CHUG phylogeny may bias the placement of the root. Long branches may result from using fast evolving sites, such as synonymous sites, in protein-coding genes and noncoding DNA. This hypothesis was validated with a phylogenomic tree construction based on the concatenated amino acid alignment of the core genes shared by the sister group and the CHUG lineage (Fig. S6A, S6B). In this amino acid sequence-based tree, however, the branch connecting CHUG and its sister group is still long compared to the branches within the CHUG lineage (Fig. S6B). We therefore employed an outgroup-free method, the minimal ancestor deviation (MAD) approach, to root the phylogenomic tree (Fig. S6C). This method considers each branch as a possible root position and infers the ancestor-descendant relationships of all possible ancestral nodes in the tree. For each relationship, the mean relative deviation from the clock-likeness is evaluated, and the branch with the minimal relative deviation is considered the one containing the root node. In the MAD analysis, the sister group was removed, and only the 33 CHUG genomes were used. We chose the phylogenomic tree based on the shared genomic DNA for MAD rooting because: (i) given the close relationship among the CHUG members, nucleotide sequences have higher resolution, and nucleotide substitution is less likely to reach saturation; and (ii) only nucleotide sequences allow Gubbins to detect and mask the recombined regions, an essential step in constructing a clonal evolutionary relationship for the CHUG lineage (82).

10.1128/mbio.00571-22.8 FIG S6 The phylogenomic trees rooted with outgroup-dependent (A and B) and outgroup-free (C) methods. (A) A Maximum Likelihood (ML) phylogenomic tree constructed using IQ-TREE with a shared genomic DNA alignment (including both protein-coding genes and other genomic regions) in which all recombined sites were masked by Gubbins. (B) A ML phylogenomic tree constructed using IQ-TREE with a concatenation of core gene alignment at the amino acid level. The root positions for the phylogenomic trees in (A and B) were determined with outgroups, which are the sister group of the CHUG lineage. Solid circles in the phylogeny indicate nodes with bootstrap values of 100% in the 1,000 bootstrapped replicates. The root position for the phylogenomic tree (B) was determined via the minimal ancestor deviation (MAD) method. Branch colors in (B) correspond to their ancestor relative deviation value (AD), which is the relative deviation from the molecular clock expectation. The root clock coefficient of variation (CCV) quantifies the distances from the inferred root to each of the OTUs, and the root ambiguity index (AI) is the ratio of the MAD to the second smallest AD. The gray arrow denotes the best root position, which shows the smallest MAD, CCV, and AI values. Download FIG S6, PDF file, 0.2 MB. Copyright © 2022 Chu et al. 2022 Chu et al. https://creativecommons.org/licenses/by/4.0/ This content is distributed under the terms of the Creative Commons Attribution 4.0 International license.

Next, genetically isolated populations were defined with PopCOGenT (83). The rationale of this method was that since bacterial speciation often proceeds rapidly, the delineation of population boundaries should be based on gene flow events that occurred recently (83). One main advantage of PopCOGenT over earlier methods, such as ConSpeciFix (84), is that it allows separating recent transfer events from historical ones (83). Ancestral nodes in the phylogenomic tree that lead to speciation events can be easily identified based on the population membership defined by PopCOGenT. Further evidence for population differentiation was provided at the core genome by computing the fixation index (F_ST_) between delineated populations using Arlequin v3.5 (85) and at the accessory genome by clustering the genomes based on the gene presence and absence using the complete linkage method implemented in the R package, “*pheatmap*” (86). Prior studies demonstrated that population differentiation in members of the marine Roseobacter group is often achieved by replacing alleles at some core genes with novel and potentially adaptive copies derived from external species (8). This mechanism was explored using the synonymous substitution rate (*d_S_*) clustering approach, which makes pairwise comparisons across single-copy orthologous genes and across all genomes under comparison (42). The rationale is that synonymous substitution is largely neutral, and thus, genes with unusually large *d_S_* values likely result from recombination-driven novel allele replacements. This approach helps to infer at which phylogenetic branches the divergent alleles were introduced into the population, so it was implemented here to determine the relative importance of allele replacement in core genes in the successive speciation events.

Since the CHUG populations differ in genes involved in carbon source utilization and oxygen respiration, we conducted physiological assays of substrate utilization through BiOLOG Phenotype Microarrays (PM) (87) and through measurements of oxygen uptake under microaerobic conditions using optical oxygen sensors (88), respectively. For the former, PM01 and PM02, which contain 190 carbon sources, were used. In addition to checking the differential utilization of the carbon sources predicted by the genetic differences, we also constructed a heat map based on the maximal respiration intensity at 96 h by using the complete linkage method implemented in the R package, “*pheatmap*” (86), to provide an overall representation of the substrate utilization among populations. For the latter, the kinetic parameters describing the oxygen uptake rates, including the maximum uptake rate (V_max_) and the apparent half-saturation constant (K_m_), were estimated. Specific affinities to oxygen were calculated following a method described by Button et al. (60). Additional methodological details are provided in Supplemental Methods in Text S1.

### Data availability.

Raw reads and the assembled genomic sequences of the new CHUG genomes are available in the NCBI GenBank database under the accession number PRJNA770146.

### Code availability.

The scripts used for the phylogenetic tree construction, F_ST_ calculation, and Biolog data processing are available from an online repository: https://github.com/luolab-cuhk/CHUG-pop-microdiversity.
