# Supplementary material for: Coastal Transient Niches Shape the Microdiversity Pattern of a Bacterioplankton Population with Reduced Genomes
Source: mBio. 2022 Jul 26;13(4):e00571-22. doi: 10.1128/mbio.00571-22 (PMC9426536; doi:10.1128/mbio.00571-22)
Supplement: FIG S1 [file mbio.00571-22-s0001.pdf]

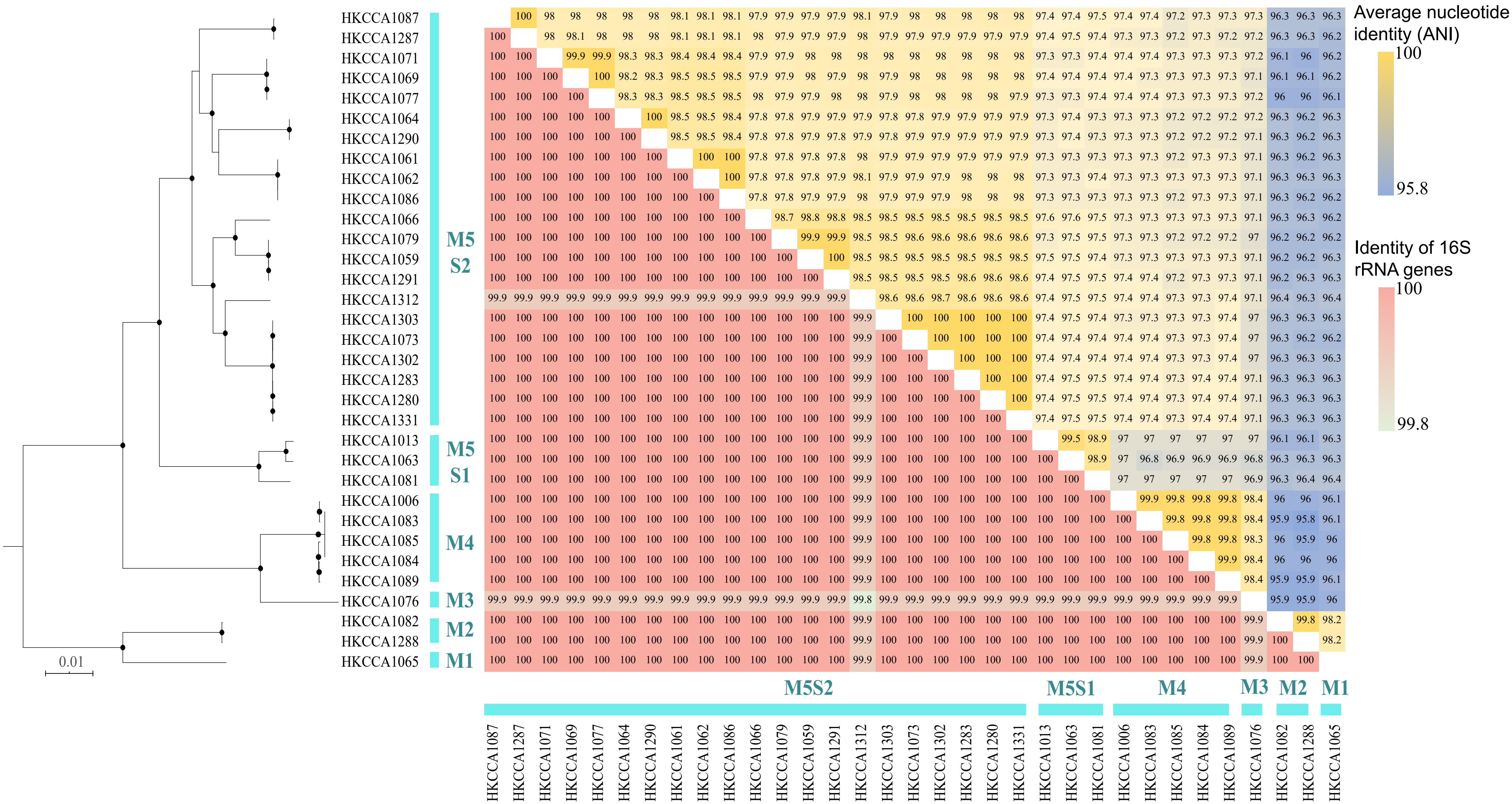

Figure S1. The heatmap of the pairwise identity of 16S rRNA genes and the whole-genome average nucleotide identity (ANI) of the 33 CHUG isolates. Genomes are arranged according to the phylogenomic tree shown on the left.
