# Supplementary material for: Coastal Transient Niches Shape the Microdiversity Pattern of a Bacterioplankton Population with Reduced Genomes
Source: mBio. 2022 Jul 26;13(4):e00571-22. doi: 10.1128/mbio.00571-22 (PMC9426536; doi:10.1128/mbio.00571-22)
Supplement: TEXT S1 [file mbio.00571-22-s0008.docx]

**Text S1. Supplemental Methods**

## 1.1 Sample collection and bacterial isolation

A single seawater sample from the *Sargassum hemiphyllum* ecosystem was collected at Lobster Bay (22.309° N, 114.3015° E), Hong Kong SAR, China on 15 Mar 2017. The sample was stored in the 50 mL Microcentrifuge tube and maintained in darkness at 4 °C until further processing. The CHUG isolation medium is the marine basal medium (MBM) modified with dimethylsulfoniopropionate (DMSP) as the sole carbon source (1). The 100-fold serial dilution of the sample was prepared with sterilized seawater, and 100 μL diluted sample was spread on the MBM-DMSP medium. The isolation plates were incubated for one week at room temperature. Colonies were selected and repeatedly re-streaked on the 2216 marine agar (BD Difco, USA) and stored at glycerol suspensions (25%, v/v) at -80°C. Templates for 16S rRNA gene amplification were prepared using a 10% (w/v) Chelex solution. Polymerase chain reaction (PCR) was conducted using 27F (5′-AGRGTTYGATYMTGGCTCAG-3′) and 1492R (5′-GGYTACCTTGTTACGACTT-3′) as bacterial specific primer. PCR products were evaluated on 0.8% agarose and viewed by the automatic analysis system of electrophoresis gel imaging. Positive PCR products were sent to BGI for sequencing and the taxonomy of 16S rRNA gene sequence was analyzed on the EzTaxon server (2). This procedure led to the isolation of 33 CHUG bacteria.

## 1.2 Genome sequencing, assembly, and annotation

Genomic DNA of each of the 33 CHUG isolates was extracted using EZ.N.A. Bacterial DNA Kit (Omega Bio-tek, Inc, USA). DNA quantity and quality were evaluated by Nanodrop. Genomes were sequenced using the BGISEQ-500 PE100 platform. Adaptors and low-quality bases of raw reads were trimmed by Trimmomatic v0.36 (3) with the parameters of ‘ILLUMINACLIP: 2:30:10:1:true SLIDINGWINDOW:4:15 MAXINFO:40:0.9 MINLEN:40’, and the quality of remaining reads was checked by FastQC v0.11.5 (http://www.bioinformatics.babraham.ac.uk/projects/fastqc). Quality reads were assembled into draft genome using SPAdes v3.9.1 (4) with default parameters, and contigs of the length over 1,000 bp were kept for further analysis. The quality of all draft genomes was assessed by CheckM v1.0.7 (5), in which three marker genes (PF05958, PF06723, and PF07991) were excluded because they are either absent or multi-copied in the closed genome HKCCA1288. Note that the genomes of two isolates (HKCCA1288 and HKCCA1065) were published in a recent study, and that the genome of HKCCA1288 was closed with additional sequencing by Nanopore (6). Then, genes were predicted and annotated for each genome assembly.

## 1.3 Calculating genetic similarity and predicting mobile genetic elements and pseudogenes

To measure the genetic similarity of population members, the 16S rRNA gene identity and the whole-genome average nucleotide identity (ANI) between pairwise strains were calculated using BLASTn (7) and FastANI (8), respectively. The pseudogenes and mobile genetic elements, including genomic islands (GIs), prophages, and insertion sequences (IS), were also predicted (see Methods in the main paper).

## 1.4 Constructing phylogenomic tree and genome content tree based on shared genomic DNA, protein sequences of core genes, and accessory genome, respectively

To construct a phylogenomic tree showing the clonal relationship of these 33 strains, five genomes constituting the sister group of the CHUG lineages (6) were used as outgroups. Briefly, we employed ProgressiveMauve v2.3.1 (9) to generate a whole genome alignment of these 38 isolates, and subsequently used the stripSubsetLCB module provided by Mauve (10) to extract the shared genomic DNA alignment which comprises the locally collinear blocks (LCBs) each with a length of >500 bp. Note that the shared genomic DNA includes both protein-coding genes and other genomic regions. Next, Gubbins 3.1.4 (11) was used to mask and remove sites subjected to recombination from the shared genomic DNA alignment. The Maximum Likelihood (ML) phylogenomic tree was subsequently constructed based on the masked alignment using IQ-TREE v2.1.3 (12) with 1,000 ultrafast bootstrap replications (13) and rooted with the sister group (Fig. S6A).

We also constructed a phylogenomic tree based on amino acid sequences of core genes. Briefly, 1,331 core genes shared by the 33 CHUG and five outgroup genomes were identified using OrthoFinder v2.2.1 (14) and each were aligned using MAFFT v7.215 (15) at amino acid sequence level. Then, the phylogeny (Fig. S6B) was constructed based on the concatenation of these alignments using IQ-TREE v2.1.3 (12) with ModelFinder (16) assigning the best substitution model and 1,000 ultrafast bootstrap replications (13).

Since the placement of the root may be biased by the long branch connecting the CHUG lineage and the sister group, an outgroup-free method was used to determine the root. Specifically, an unrooted phylogenomic tree was constructed based on the shared genomic DNA alignment of the 33 CHUG members with the recombined sites masked by Gubbins 3.1.4 (11). Then, the unrooted tree was rooted using the minimal ancestor deviation (MAD) approach (17) (Fig. S6C). The trees were visualized with iTOL (18).

To assess the genome similarity at the genome content level, orthologous gene families were first identified by Roary v3.13.0 (19) with default parameters. Next, the presence and absence matrix of all accessory gene families was used to construct a dendrogram using the complete-linkage method implemented in R package *pheatmap* (20). To test the hypothesis that genomic islands (GIs), where > 60% of the accessory genes are found, may play a part in diversifying this entire CHUG population, the GIs-associated gene families were clustered using the complete-linkage method implemented in R package *pheatmap* (20) to construct a dendrogram.

## 1.5 Population structure inference and recombination analysis

A recently developed program PopCOGenT (21) was employed to delineate genetically isolated populations included in the 33 CHUG members we sampled. Briefly, PopCOGenT uses enrichment of identical regions between pairwise genomes as a proxy of recent recombination. Although both vertical inheritance and homologous recombination could produce identical genomic regions, the length and frequency distribution of identical regions are different. This is because time is not long enough for recent recombination to accumulate many SNPs differentiating the genomic regions.

The fixation index (F_ST_) was further used to investigate the level of differentiation. For this analysis, 1,788 single-copy orthologous genes shared by the 33 genomes were first identified using Roary v3.13.0 (19), and then they were aligned with MAFFT v7.215 (15) at the amino acid level with DNA sequences subsequently imposed on the alignment. Next, F_ST_ for each single-copy core gene family was calculated using Arlequin v.3.5 (22) with 10,000 permutations performed to test statistical significance of allele fixation.

ClonalFrameML (23) was implemented to estimate the relative rate (ρ/θ) and effect (r/m) of recombination to mutation, with the above-mentioned core genomic alignment and the phylogenomic tree as inputs. ClonalFrameML infers recombination under the ClonalFrame model (24), which detects recombination based on clustered SNPs which are primarily imported from external sources.

## 1.6 Detecting genomic regions underlying the population differentiation

In accessory genome, the population-specific gene families that are exclusively present in one population but absent from another likely contributed to population differentiation. They were identified from the gene families clustered with Roary v3.13.0. One caveat is that when using incomplete genomes, a possibility that “missing genes” are not truly missing but result from incomplete genome assembly cannot be ruled out. Hence, the completeness of genome assembly may affect the accuracy of the identified population-specific gene families. While most genomes (32 out of the 33 CHUG isolates) are not closed, they show very high completeness and their assembly sizes are comparable to the genome size of the closed strain HKCCA1288 (Data Set S1a). Furthermore, the few missing genes due to assembly issue are not necessarily members of the gene families of interest; in theory, they can be any type of gene. We therefore argue that the identified population-specific gene families are overall reliable.

Genes in the core genome can also play an important role in pelagic Roseobacter population differentiation by novel allele replacement through recombination with external lineages (25). Novel allele replacements can be detected by identifying core genes with unusually large synonymous substitution rate (*d_S_*), a method initially developed by (26) and employed in (25). The principle is that changes at synonymous sites are largely neutral and mutation occurs randomly across the whole genome. If a divergent allele was introduced through recombination, this gene may show an unusually large *d_S_* value due to potentially clustered substitutions, compared to that of other genes not affected. It is worth noting that synonymous sites are not strictly neutral, as nutrient limitation (27, 28) would change the G+C content at synonymous (and other) sites and codon usage bias could impose translational selection on synonymous sites (29). However, since the former indiscriminately affects all genes and the latter mainly decreases the *d_S_* values of highly expressed genes, these two mechanisms would not yield a set of gene families with unusually large *d_S_* values.

In practice, the bioinformatics procedure is as follows: first, pairwise *d_S_* values were estimated for each of the 1,788 core gene families using the YN00 module in PAML (30). Five core gene families with abnormal *d_S_* values (with *d_S_* = 99) were ignored. The remaining 1,783 gene families were clustered based on their pairwise *d_S_* values using the k-means clustering algorithm, with the optimal number of clusters was set as two based on a voting mechanism using the R package ‘NbClust’ (31). The Cluster-I consists of gene families subjected to novel allele replacements as these genes show much larger median *d_S_* values than the other cluster. For each gene family of Cluster-I, the median *d_S_* values of pairwise comparisons from M1M2 and M3M4M5 are much greater than those of pairwise comparisons from M1M2 or M3M4M5 (Data Set S1b), suggesting that the replacement likely occurred at, but not limited to, the last common ancestor (LCA) of either M1M2 or M3M4M5. To more precisely map allelic replacements to the evolutionary branches of the species tree, we compared the pattern of *d_S_* values, the gene tree topology, and the genome tree. All gene trees were constructed based on the nucleotide alignments mentioned above using IQ-TREE with nucleotide substitution model determined by ModelFinder. Since the CHUG population is distantly related to its sister group (6) and the appropriate outgroups are not available, these gene trees were rooted at mid-point using R package *ape* (32).

## 1.7 BiOLOG Phenotype Microarray (PM) assay

To determine the utilization of substrates for different populations, the BiOLOG Phenotype Microarry (PM) (33) PM01 and PM02 that contain 190 carbon sources were used to assay the phenotypic differences between different populations defined by PopCOGenT (represented by the strain HKCCA1065 from the population M1, HKCCA1288 from M2, HKCCA1006 from M4, HKCCA1013 and HKCCA1081 from M5S1, and HKCCA1086 from M5S2). Since the biomass was too sticky to be mixed well in the BiOLOG inoculating fluid IF-0a when cultured on the agar, we used the liquid medium to culture these strains. Other procedures for the BiOLOG PM test were the same as the previous work (25). Briefly, the six sampled strains were cultured in Marine 2216 broth (Difco) at 30 °C for two days. Then, a total of 600 mL liquid culture for each strain was centrifuged in the sterilized centrifuge bottle at room temperature at 3,000 g for 10 min to remove the culture medium. Next, cell pellets were resuspended in 300 mL autoclaved artificial seawater and centrifuged at 3,000 g for 5 min, and these steps were repeated to further remove the initial medium. In a preliminary experiment, we found that this protocol resulted in no growth and thus no purple color in the negative control wells for both PM01 and PM02 plates. Therefore, cell pellets were collected and suspended using a modified IF-0a inoculation medium (2% (m/w) final concentration of NaCl). Cell suspension was adjusted to 60% using the BiOLOG turbidity meter and mixed with the redox Dye Mixes D (final concentration to 1.5X). 100 μL aliquot of the cell mixture was homogenized and inoculated in each well of the plates, and three replicates were performed for each strain. All PM plates were incubated, and the growth curve data was collected using the OmniLog instrument. Through recording the color change caused by cells’ respiration over time, a kinetic respiratory activity curve which parallels microbial growth curve was generated for each substrate and can be used to compare carbon source utilization between isolates.

After incubating the PM plates for 96 hours, all raw data were converted by BiOLOG software and analyzed using the ‘opm’ package (34). To obtain an overall picture for the substrate utilization among representative strains, a heatmap was constructed based on the maximal respiration intensity at 96 hours for each isolate, with the background noise (BiOLOG PM A1) removed using the software "OmniLog-OL_PM_FM/Kin 1.30-: File Management/Kinetic Plot Version software of Biolog". Next, abnormal values which may result from the crystal of bacterial metabolites or biomass sedimentation were excluded. For example, the result of one replicate of strain HKCCA1013 at PM01 A10 (D-Trehalose) (Fig. S1) showing an unusual peak was removed. For each substrate, the respiration value of each strain was averaged using results of all replicates, and then the mean respiration value was further used for clustering. The similarity between strains across all substrates’ utilization was measured by Euclidean distance, and then the distance matrix was clustered with complete-linkage method implemented in the R package ‘*pheatmap*’(20).

## 1.8 Oxygen uptake measurements

Oxygen uptake rates were determined in a suite of incubations in which oxygen consumption was determined as a function of time. Four CHUG strain from different clusters (HKCCA1065 from M1, HKCCA1288 from M2, HKCCA1006 from M4, and HKCCA1086 from M5S2) and a *Roseobacter* model strain *Ruegeria pomeroyi* DSS-3 were pregrown in 2216 marine broth (BD Difco, USA) at 30 °C and 200 rpm for 1 to 2 days. The cultures were then diluted to a standardized cell titre with an average absorbance of 0.15 (± 0.01 1SD) using the cell-free medium. The absorbances were measured on a NanoDrop One^C^ Microvolume UV-Vis Spectrophotometer (Thermo Fisher) at 600 nm. Before inoculating the cells, the media were transferred aseptically to autoclaved glass serum bottles (Kimble) which with a trace oxygen sensor spot (TROXSP5, Pyroscience) fixed to the inner wall and a sterilized glass-coated magnetic stir bar (Sigma) placed on the bottle. Serum bottles were closed with sterile, thick, blue butyl rubber stoppers and the media were purged for around 15 min with N_2_ gas to decrease media oxygen concentrations to approximately 5 µmol O_2_ L^-1^. The headspace in the bottle was eliminated with the addition of anoxic media to a final volume of 250 ml. To minimize the influence of temperature variations on the optode oxygen measurements, the serum bottles were placed in a water bath thermostatted at ambient temperature (25°C). A temperature sensor (Pyroscience) was placed into the water bath which allows automatic real-time temperature compensation. A stir plate (IKA Labortechnick) was placed underneath each bottle to ensure the medium was well-mixed. The incubation experiment began by adding 1 ml of culture with adjusted cell titre to the incubation bottle. The fluorescent signals from the optodes were read via optical fibres (Pyroscience) positioned outside the bottles and oxygen concentrations were measured every second and recorded using the software Pyro Workbench V1.2.0.1359.

Oxygen uptake rates were calculated from linear regression of data recorded by the optodes. The raw data were first smoothed with a 6^th^ order polynomial. Rates were calculated as the slopes of linear regressions over oxygen consumption intervals at different oxygen concentrations: from 2 to 1 µmol O_2_ L^-1^, slopes were calculated for every 0.2 µmol O_2_ L^-1^; from 1,000 to 100 nmol O_2_ L^-1^ for every 100 nmol O_2_ L^-1^; and from 100 to 60 nmol O_2_ L^-1^ for every 10 nmol O_2_ L^-1^. Non-linear regression was then used to fit a Michaelis-Menten type enzyme kinetic model (MM) to the resulting rate data as a function of oxygen concentrations, and the kinetic parameters, Vmax (maximum uptake rate) and K_m_ (apparent half-saturation constant) were calculated.

$$R=V_{max}\times\frac{[O_{2}]}{K_{m}+[O_{2}]}$$

Here R represents the oxygen uptake rate, V_max_ is the maximum uptake rate, which is at the highest oxygen concentration ([O_2_]), and K_m_ is the oxygen concentration at half the maximum rate. We also estimated kinetic parameters using the Hanes-Woolf linearization. The linearization was performed with the data collected between 100 and 1000 nmol O_2_ L^-1^ due to deviation from linearity below 100 nmol O_2_ L^-1^.

Specific affinities of oxygen were calculated by diving biomass specific V_max_ by the K_m_ value (35). Biomass-specific V_max_ was determined by dividing the yielded volume-specific V_max_ by total biomass. Total biomass was calculated according to Khachikyan and fellows (36). Briefly, cell volumes were calculated using the following equation by assuming that cell width equals cell height:

$$V=\pi r^{2}\times h +\frac{4}{3}\pi r^{3}$$

where$V$ is the cell volume in cubic micrometres, $r$is the radius of a cell, and $h=L-2r$, where L is the length of the cell. Cell volumes of *R. pomeroyi* DSS-3 were calculated by assuming the size of cells were 1.20 µm long with a radius of 0.35 µm (37). For CHUG isolates, cell volumes were calculated by assuming the size of cells were 2.6 µm long with a radius of 0.3 µm (38). The dry mass in femtograms ($m_{dry}$) was then calculated by the following equation:

$$m_{dry}=322\times V^{0.43}$$

Total biomass of each incubation was then calculated according to the total cell number. The total cell number was determined by converting the absorbance at a wavelength of 600 nm to cell numbers based on a working curve of cell concentrations determined by flow cytometry vs. absorbance. Cell-specific V_max_ was also determined by dividing the volume-specific V_max_ by the total cell number.

# **References**

1. Chu X, Li S, Wang S, Luo D, Luo H. 2021. Gene loss through pseudogenization contributes to the ecological diversification of a generalist Roseobacter lineage. 2. ISME J 15:489–502.

2. Chun J, Lee J-H, Jung Y, Kim M, Kim S, Kim BK, Lim Y-W. 2007. Eztaxon: A web-based tool for the identification of prokaryotes based on 16s ribosomal rna gene sequences. Int J Syst Evol Microbiol 57:2259–2261.

3. Bolger AM, Lohse M, Usadel B. 2014. Trimmomatic: A flexible trimmer for Illumina sequence data. Bioinformatics 30:2114–2120.

4. Bankevich A, Nurk S, Antipov D, Gurevich AA, Dvorkin M, Kulikov AS, Lesin VM, Nikolenko SI, Pham S, Prjibelski AD. 2012. SPAdes: A new genome assembly algorithm and its applications to single-cell sequencing. J Comput Biol 19:455–477.

5. Parks DH, Imelfort M, Skennerton CT, Hugenholtz P, Tyson GW. 2015. CheckM: Assessing the quality of microbial genomes recovered from isolates, single cells, and metagenomes. Genome Res 25:1043–1055.

6. Feng X, Chu X, Qian Y, Henson MW, Lanclos VC, Qin F, Barnes S, Zhao Y, Thrash JC, Luo H. 2021. Mechanisms driving genome reduction of a novel Roseobacter lineage. ISME J https://doi.org/10.1038/s41396-021-01036-3.

7. Boratyn GM, Camacho C, Cooper PS, Coulouris G, Fong A, Ma N, Madden TL, Matten WT, McGinnis SD, Merezhuk Y. 2013. Blast: A more efficient report with usability improvements. Nucleic Acids Res 41:W29–W33.

8. Jain C, Rodriguez-R LM, Phillippy AM, Konstantinidis KT, Aluru S. 2018. High throughput ANI analysis of 90K prokaryotic genomes reveals clear species boundaries. Nat Commun 9:1–8.

9. Darling AE, Mau B, Perna NT. 2010. ProgressiveMauve: Multiple genome alignment with gene gain, loss and rearrangement. PLoS One 5:e11147.

10. Darling ACE, Mau B, Blattner FR, Perna NT. 2004. Mauve: multiple alignment of conserved genomic sequence with rearrangements. Genome Res 14:1394–1403.

11. Croucher NJ, Page AJ, Connor TR, Delaney AJ, Keane JA, Bentley SD, Parkhill J, Harris SR. 2015. Rapid phylogenetic analysis of large samples of recombinant bacterial whole genome sequences using Gubbins. Nucleic Acids Res 43:e15–e15.

12. Nguyen L-T, Schmidt HA, Von Haeseler A, Minh BQ. 2015. IQ-TREE: A fast and effective stochastic algorithm for estimating maximum-likelihood phylogenies. Mol Biol Evol 32:268–274.

13. Hoang DT, Chernomor O, Von Haeseler A, Minh BQ, Vinh LS. 2018. UFBoot2: Improving the ultrafast bootstrap approximation. Mol Biol Evol 35:518–522.

14. Emms DM, Kelly S. 2015. OrthoFinder: solving fundamental biases in whole genome comparisons dramatically improves orthogroup inference accuracy. Genome Biol 16:157.

15. Katoh K, Standley DM. 2013. MAFFT multiple sequence alignment software version 7: Improvements in performance and usability. Mol Biol Evol 30:772–780.

16. Kalyaanamoorthy S, Minh BQ, Wong TKF, von Haeseler A, Jermiin LS. 2017. ModelFinder: fast model selection for accurate phylogenetic estimates. 6. Nat Methods 14:587–589.

17. Tria FDK, Landan G, Dagan T. 2017. Phylogenetic rooting using minimal ancestor deviation. Nat Ecol Evol 1:1–7.

18. Letunic I, Bork P. 2019. Interactive Tree Of Life (iTOL) v4: recent updates and new developments. Nucleic Acids Res 47:W256–W259.

19. Page AJ, Cummins CA, Hunt M, Wong VK, Reuter S, Holden MT, Fookes M, Falush D, Keane JA, Parkhill J. 2015. Roary: Rapid large-scale prokaryote pan genome analysis. Bioinformatics 31:3691–3693.

20. Kolde R, Kolde MR. 2015. Package ‘pheatmap.’ R package 1:790.

21. Arevalo P, VanInsberghe D, Elsherbini J, Gore J, Polz MF. 2019. A reverse ecology approach based on a biological definition of microbial populations. Cell 178:820–834.

22. Excoffier L, Lischer HE. 2010. Arlequin suite ver 3.5: A new series of programs to perform population genetics analyses under linux and windows. Mol Ecol Resour 10:564–567.

23. Didelot X, Wilson DJ. 2015. ClonalFrameML: efficient inference of recombination in whole bacterial genomes. PLoS Comput Biol 11:e1004041.

24. Didelot X, Falush D. 2007. Inference of bacterial microevolution using multilocus sequence data. Genetics 175:1251–1266.

25. Wang X, Zhang Y, Ren M, Xia T, Chu X, Liu C, Lin X, Huang Y, Chen Z, Yan A, Luo H. 2020. Cryptic speciation of a pelagic Roseobacter population varying at a few thousand nucleotide sites. ISME J 14:3106–3119.

26. Sun Y, Luo H. 2018. Homologous recombination in core genomes facilitates marine bacterial adaptation. Appl Environ Microbiol 84:e02545-17.

27. Luo H, Thompson LR, Stingl U, Hughes AL. 2015. Selection maintains low genomic GC content in marine SAR11 lineages. Mol Biol Evol 32:2738–2748.

28. Hellweger FL, Huang Y, Luo H. 2018. Carbon limitation drives GC content evolution of a marine bacterium in an individual-based genome-scale model. ISME J 12:1180–1187.

29. Brandis G, Hughes D. 2016. The selective advantage of synonymous codon usage bias in *Salmonella*. PLoS Genet 12:e1005926.

30. Yang Z. 2007. PAML 4: Phylogenetic analysis by maximum likelihood. Mol Biol Evol 24:1586–1591.

31. Charrad M, Ghazzali N, Boiteau V, Niknafs A. 2014. Nbclust: An R package for determining the relevant number of clusters in a data set. J Stat Softw 61:1–36.

32. Paradis E, Schliep K. 2019. Ape 5.0: An environment for modern phylogenetics and evolutionary analyses in R. Bioinformatics 35:526–528.

33. Bochner BR, Gadzinski P, Panomitros E. 2001. Phenotype microarrays for high-throughput phenotypic testing and assay of gene function. Genome Res 11:1246–1255.

34. Vaas LA, Sikorski J, Hofner B, Fiebig A, Buddruhs N, Klenk H-P, Göker M. 2013. Opm: An R package for analysing omnilog® phenotype microarray data. Bioinformatics 29:1823–1824.

35. Button DK. 1998. Nutrient uptake by microorganisms according to kinetic parameters from theory as related to cytoarchitecture. Microbiol Mol Biol Rev 62:636–645.

36. Khachikyan A, Milucka J, Littmann S, Ahmerkamp S, Meador T, Könneke M, Burg T, Kuypers MM. 2019. Direct cell mass measurements expand the role of small microorganisms in nature. Appl Environ Microbiol 85:e00493-19.

37. Gao J, Pan H, Xiao T, Barbier G, Wang Z, Yue H, Sun S, Nitsche S, Bernadac A, Pradel N. 2006. Isolation and characterization of novel marine Roseobacter clade members producing unique intracellular chromium-rich aggregates. Res Microbiol 157:714–719.

38. Rathgeber C, Yurkova N, Stackebrandt E, Schumann P, Beatty JT, Yurkov V. 2005. *Roseicyclus mahoneyensis*gen. nov., sp. nov., an aerobic phototrophic bacterium isolated from a meromictic lake. Int J Syst Evol Microbiol 55:1597–1603.
