# Supplementary material for: Coastal Transient Niches Shape the Microdiversity Pattern of a Bacterioplankton Population with Reduced Genomes
Source: mBio. 2022 Jul 26;13(4):e00571-22. doi: 10.1128/mbio.00571-22 (PMC9426536; doi:10.1128/mbio.00571-22)

Figure S2. Allelic replacements of 25 outlier gene families that support the divergence between M1M2 and M3M4M5. For each gene family, the left is the phylogenomic tree, where arrows represent novel allelic replacement. Dark arrows show the novel allelic replacements by recombination with external lineages that are phylogenetically separated from the 33 CHUG isolates. Dotted arrows denote the recombination between populations. The right one is a gene tree, where different populations are marked with different colors. These gene families are classified as (A) Carbohydrate metabolism, (B) Membrane transport, (C) Respiration, (D) Nucleosides and nucleotides metabolism, (E) Amino acids and derivatives metabolism, (F) DNA metabolism, (G) RNA metabolism, (H) Metabolism of aromatic compounds, (I) Cell division and cell cycle, (J) Unclassified protein.

A ***iolG*** **(HKCCA1288_00139)**

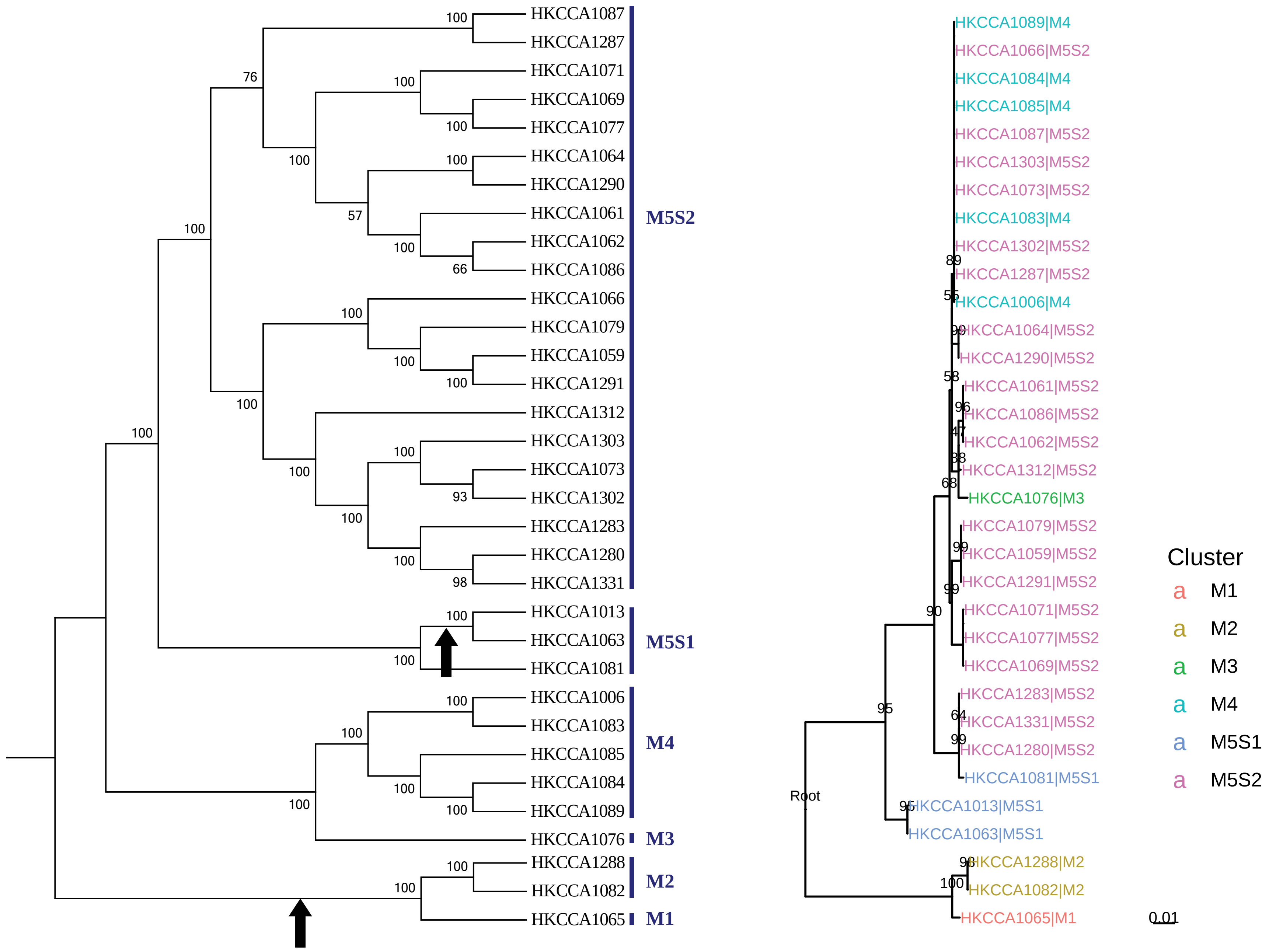

A ***msmX*** (HKCCA1288_00427)

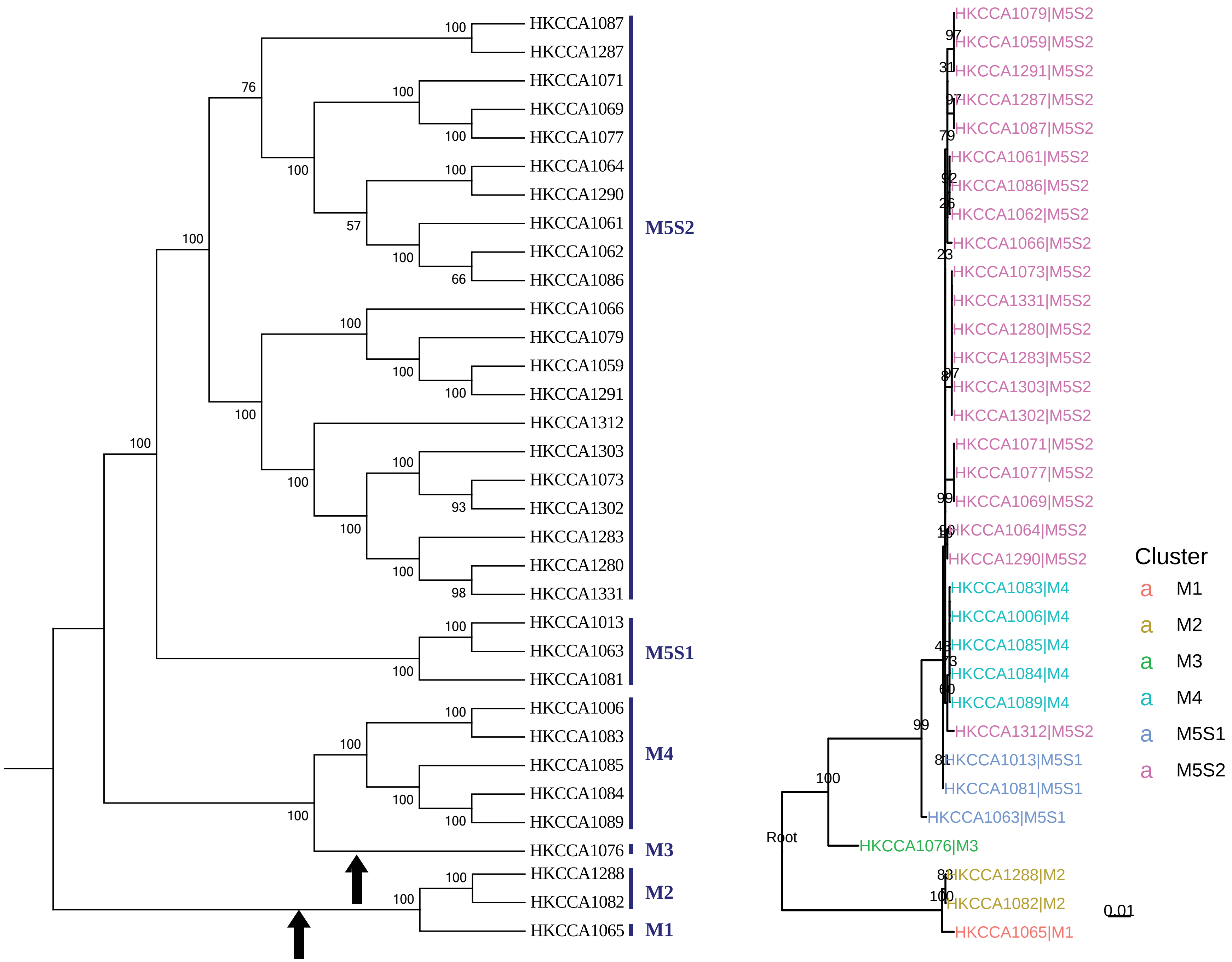

A ***xylD*** (HKCCA1288_01102)

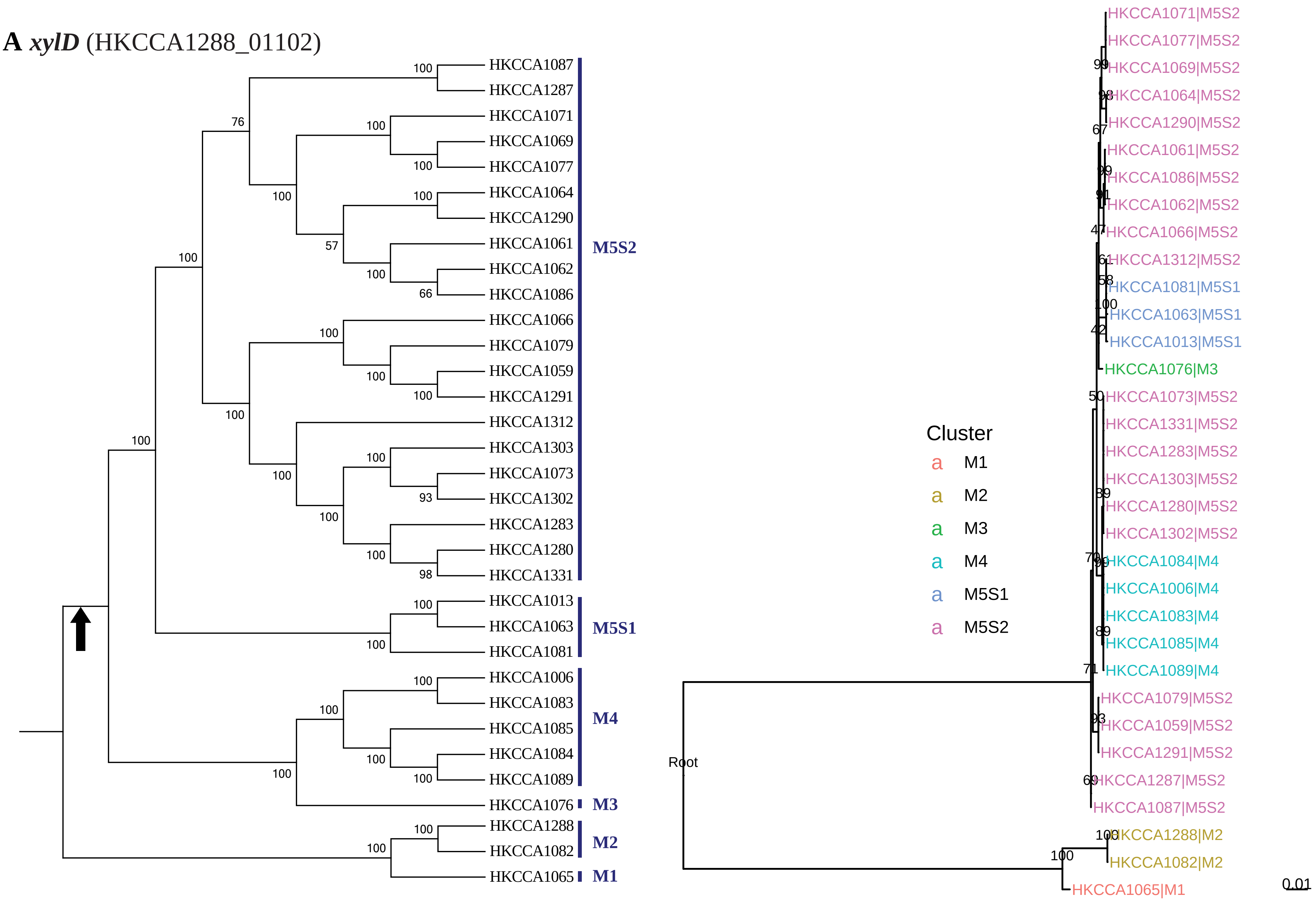

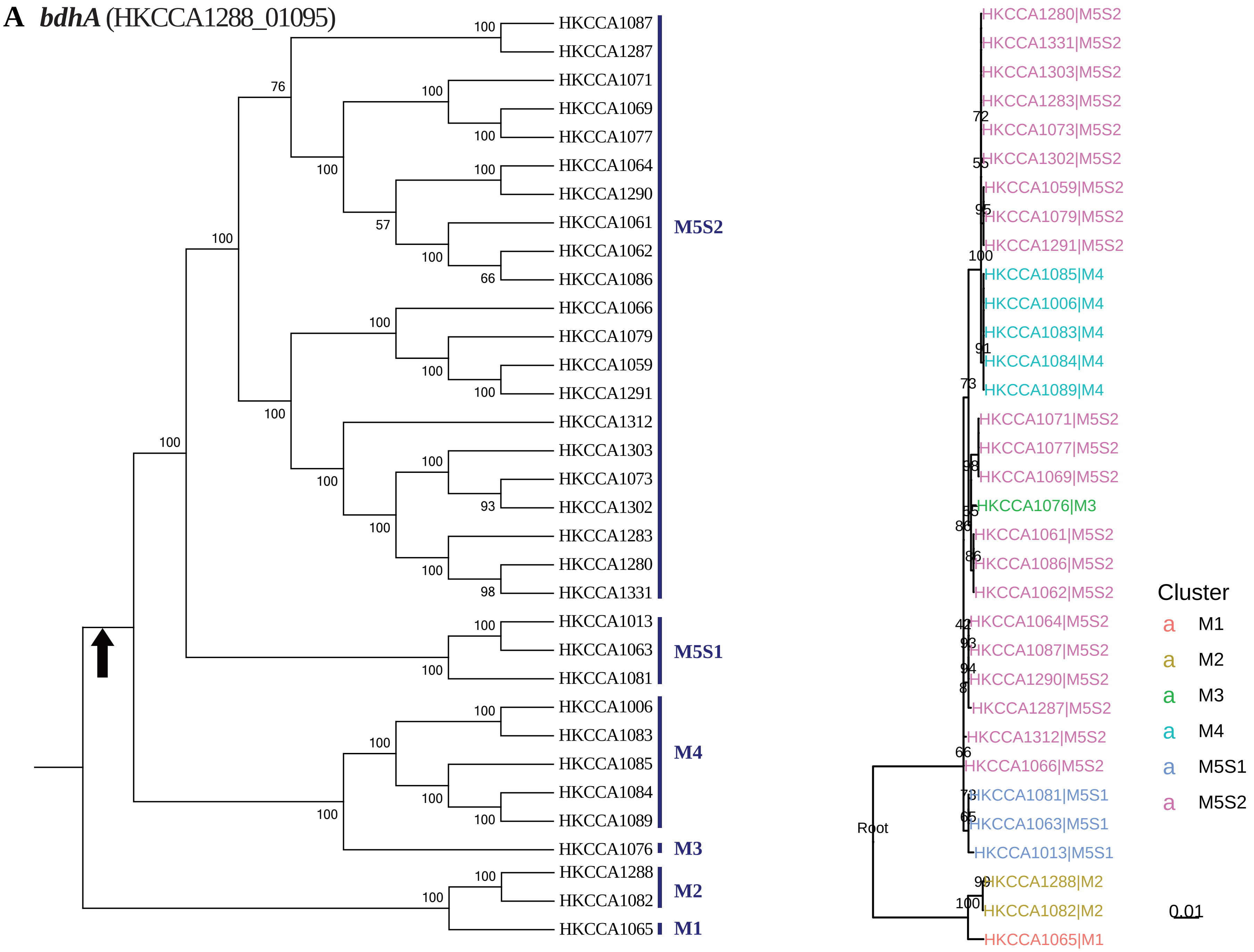
A bdhA (HKCCA1288_01095)
HKCCA1087
HKCCA1287
HKCCA1071
HKCCA1069
HKCCA1077
HKCCA1064
HKCCA1290
HKCCA1061
HKCCA1062
HKCCA1086
HKCCA1066
HKCCA1079
HKCCA1059
HKCCA1291
HKCCA1312
HKCCA1303
HKCCA1073
HKCCA1302
HKCCA1283
HKCCA1280
HKCCA1331
HKCCA1013
HKCCA1063
HKCCA1081
HKCCA1006
HKCCA1083
HKCCA1085
HKCCA1084
HKCCA1089
HKCCA1076
HKCCA1288
HKCCA1082
HKCCA1065
M5S2
M5S1
M4
M3
M2
M1
HKCCA1280|M5S2
HKCCA1331|M5S2
HKCCA1303|M5S2
HKCCA1283|M5S2
HKCCA1073|M5S2
HKCCA1302|M5S2
HKCCA1059|M5S2
HKCCA1079|M5S2
HKCCA1291|M5S2
HKCCA1085|M4
HKCCA1006|M4
HKCCA1083|M4
HKCCA1084|M4
HKCCA1089|M4
HKCCA1071|M5S2
HKCCA1077|M5S2
HKCCA1069|M5S2
HKCCA1076|M3
HKCCA1061|M5S2
HKCCA1086|M5S2
HKCCA1062|M5S2
HKCCA1064|M5S2
HKCCA1087|M5S2
HKCCA1290|M5S2
HKCCA1287|M5S2
HKCCA1312|M5S2
HKCCA1066|M5S2
HKCCA1081|M5S1
HKCCA1063|M5S1
HKCCA1013|M5S1
HKCCA1288|M2
HKCCA1082|M2
HKCCA1065|M1
Root
0.01
Cluster
a M1
a M2
a M3
a M4
a M5S1
a M5S2

A ***fbp*** (HKCCA1288_02487)

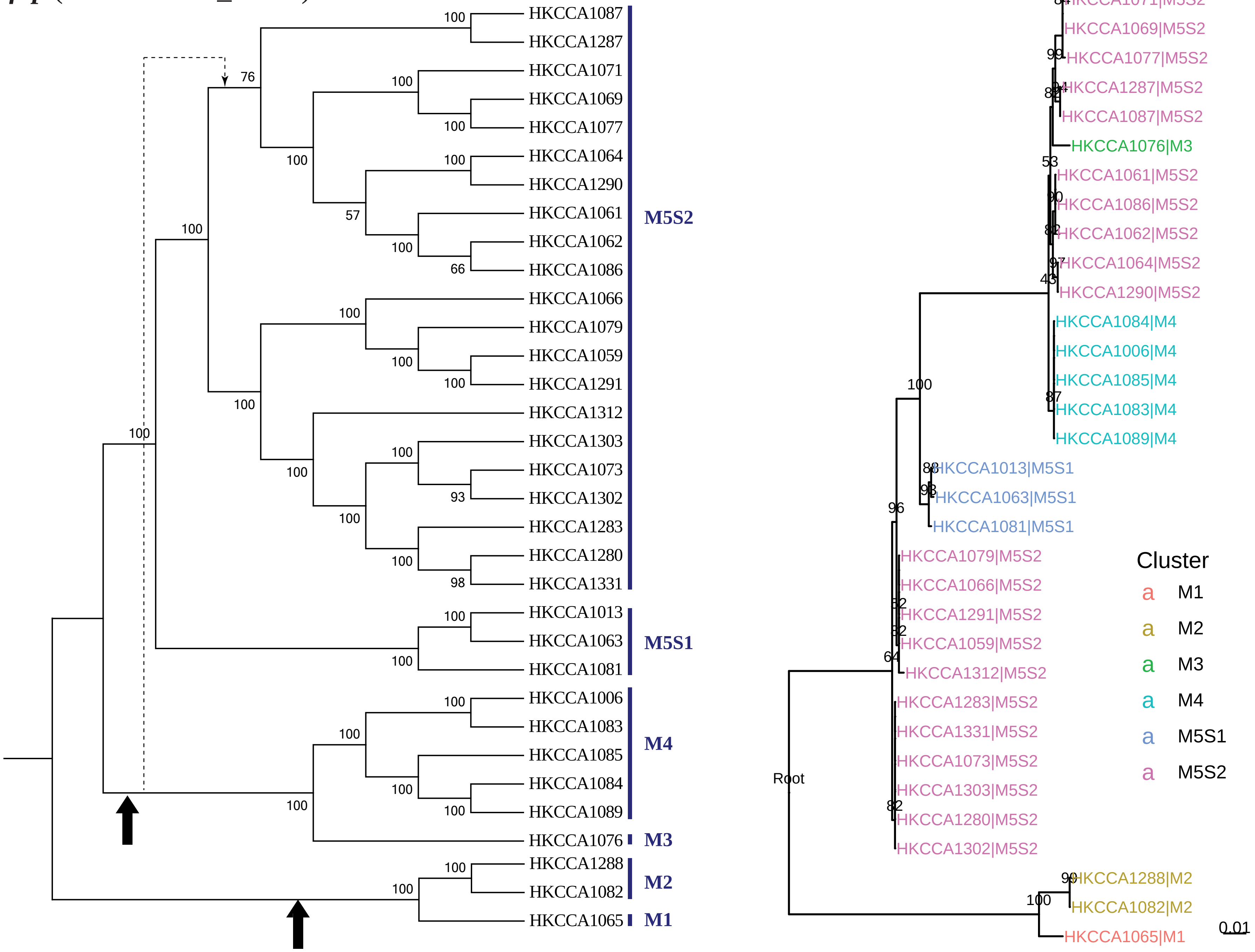

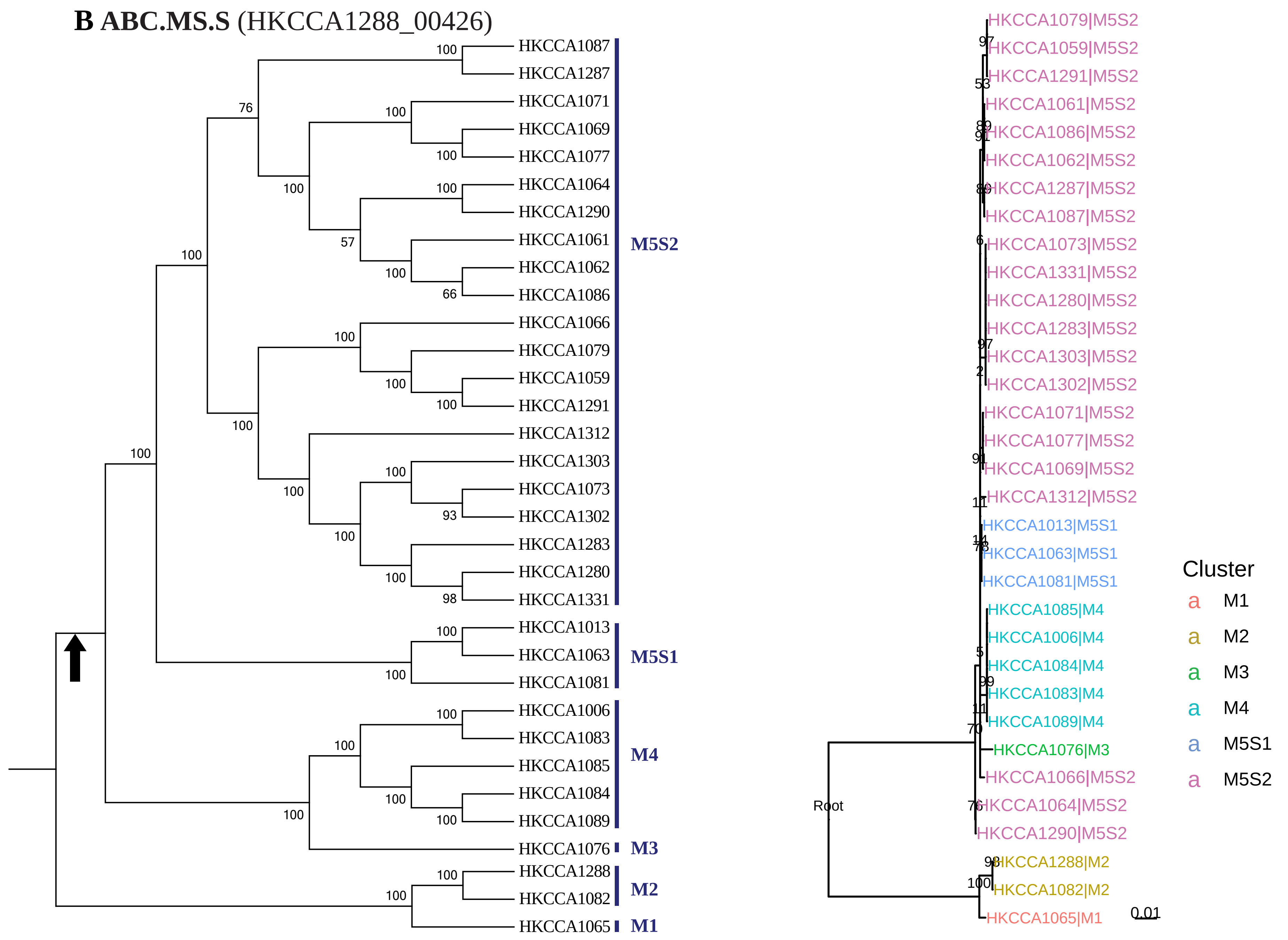

B ABC.MS.S (HKCCA1288_00426)
HKCCA1087
HKCCA1287
HKCCA1071
HKCCA1069
HKCCA1077
HKCCA1064
HKCCA1290
HKCCA1061
HKCCA1062
HKCCA1086
HKCCA1066
HKCCA1079
HKCCA1059
HKCCA1291
HKCCA1312
HKCCA1303
HKCCA1073
HKCCA1302
HKCCA1283
HKCCA1280
HKCCA1331
HKCCA1013
HKCCA1063
HKCCA1081
HKCCA1006
HKCCA1083
HKCCA1085
HKCCA1084
HKCCA1089
HKCCA1076
HKCCA1288
HKCCA1082
HKCCA1065
M5S2
M5S1
M4
M3
M2
M1
HKCCA1079|M5S2
HKCCA1059|M5S2
HKCCA1291|M5S2
HKCCA1061|M5S2
HKCCA1086|M5S2
HKCCA1062|M5S2
HKCCA1287|M5S2
HKCCA1087|M5S2
HKCCA1073|M5S2
HKCCA1331|M5S2
HKCCA1280|M5S2
HKCCA1283|M5S2
HKCCA1303|M5S2
HKCCA1302|M5S2
HKCCA1071|M5S2
HKCCA1077|M5S2
HKCCA1069|M5S2
HKCCA1312|M5S2
HKCCA1013|M5S1
HKCCA1063|M5S1
HKCCA1081|M5S1
HKCCA1085|M4
HKCCA1006|M4
HKCCA1084|M4
HKCCA1083|M4
HKCCA1089|M4
HKCCA1076|M3
HKCCA1066|M5S2
HKCCA1064|M5S2
HKCCA1290|M5S2
HKCCA1288|M2
HKCCA1082|M2
HKCCA1065|M1
Root
0.01
Cluster
a M1
a M2
a M3
a M4
a M5S1
a M5S2

B ***dctP*** (HKCCA1288_00707)

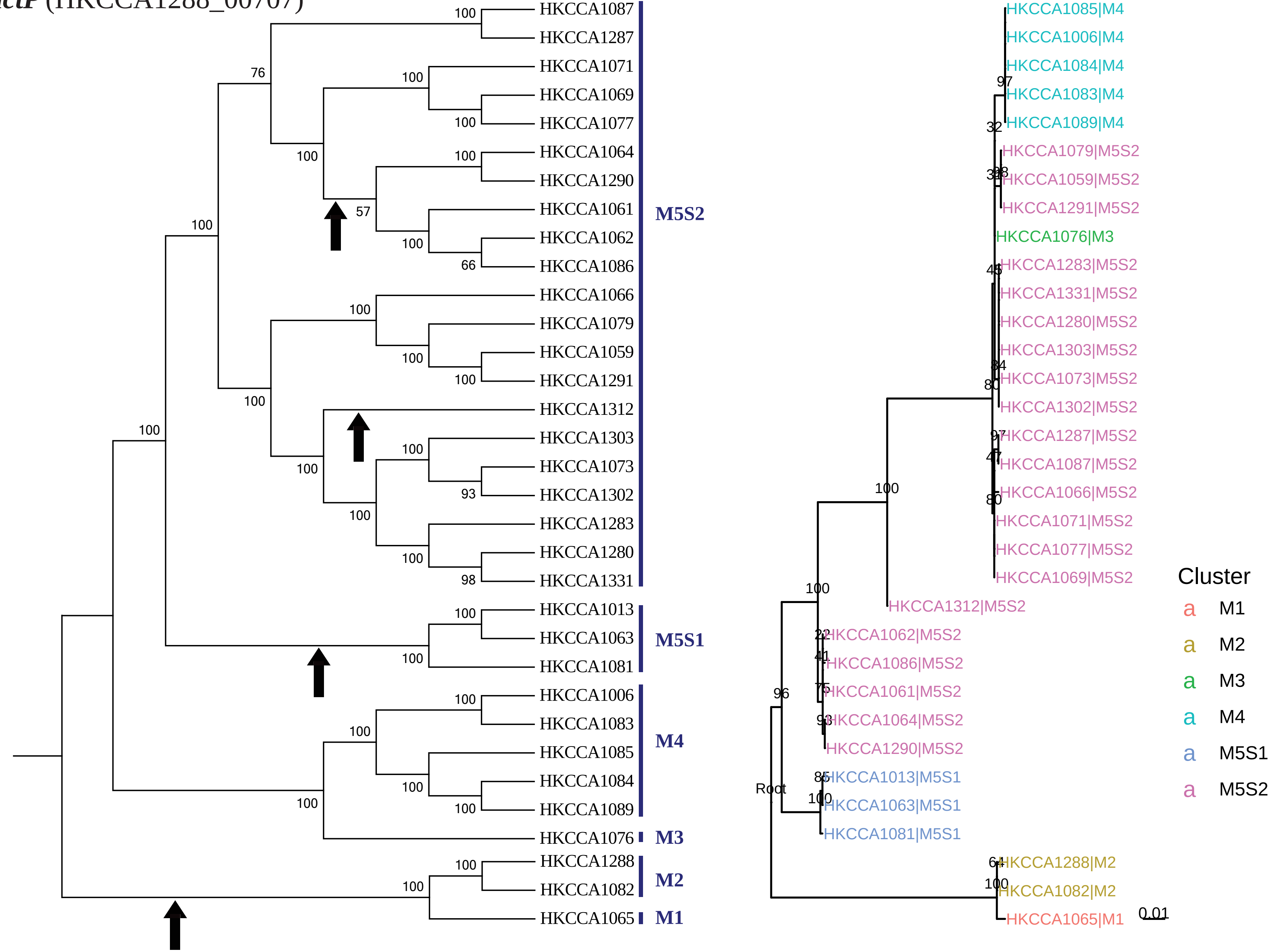

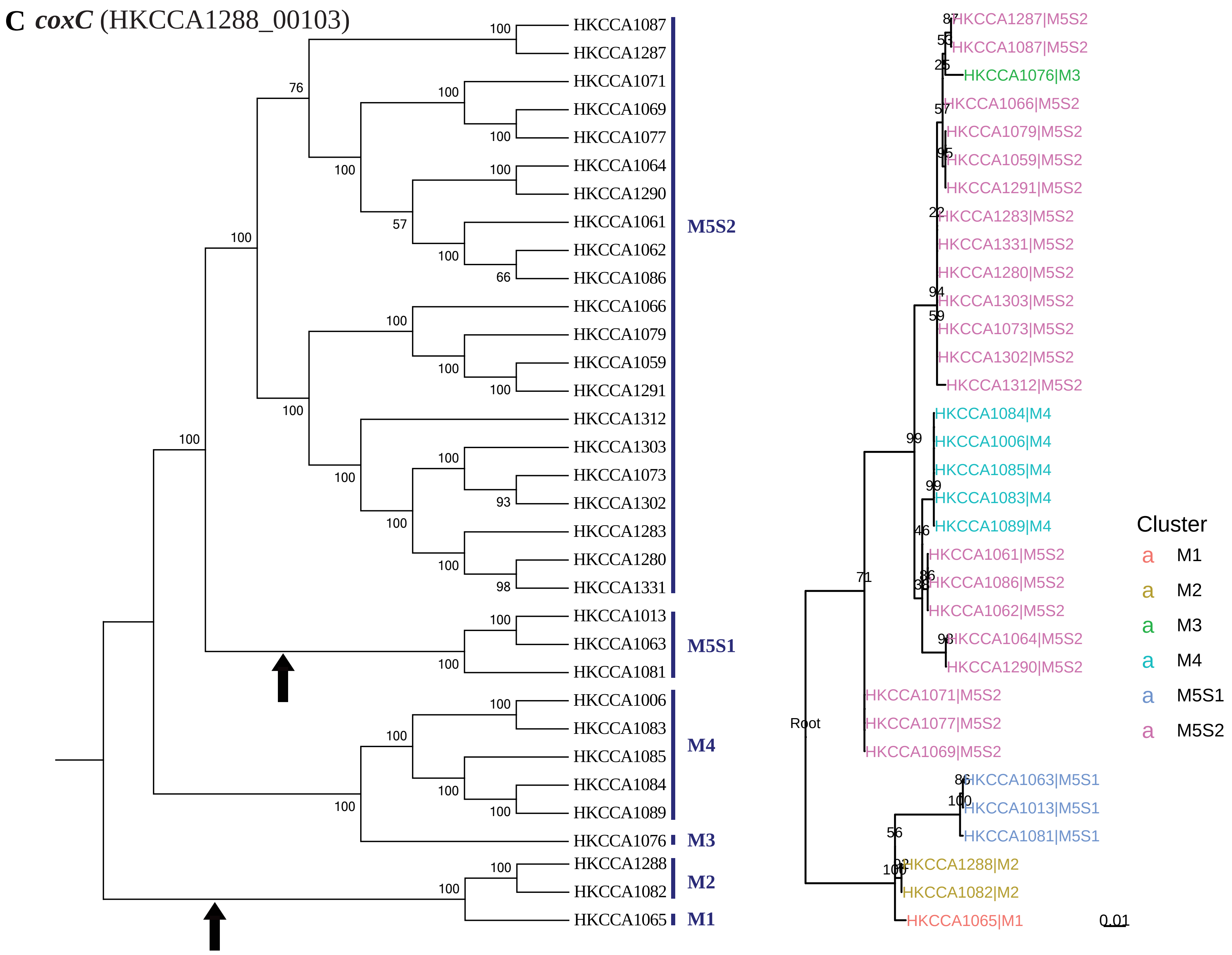
C coxC (HKCCA1288_00103)
HKCCA1087
HKCCA1287
HKCCA1071
HKCCA1069
HKCCA1077
HKCCA1064
HKCCA1290
HKCCA1061
HKCCA1062
HKCCA1086
HKCCA1066
HKCCA1079
HKCCA1059
HKCCA1291
HKCCA1312
HKCCA1303
HKCCA1073
HKCCA1302
HKCCA1283
HKCCA1280
HKCCA1331
HKCCA1013
HKCCA1063
HKCCA1081
HKCCA1006
HKCCA1083
HKCCA1085
HKCCA1084
HKCCA1089
HKCCA1076
HKCCA1288
HKCCA1082
HKCCA1065
M5S2
M5S1
M4
M3
M2
M1
Root
0.01
Cluster
M1
M2
M3
M4
M5S1
M5S2

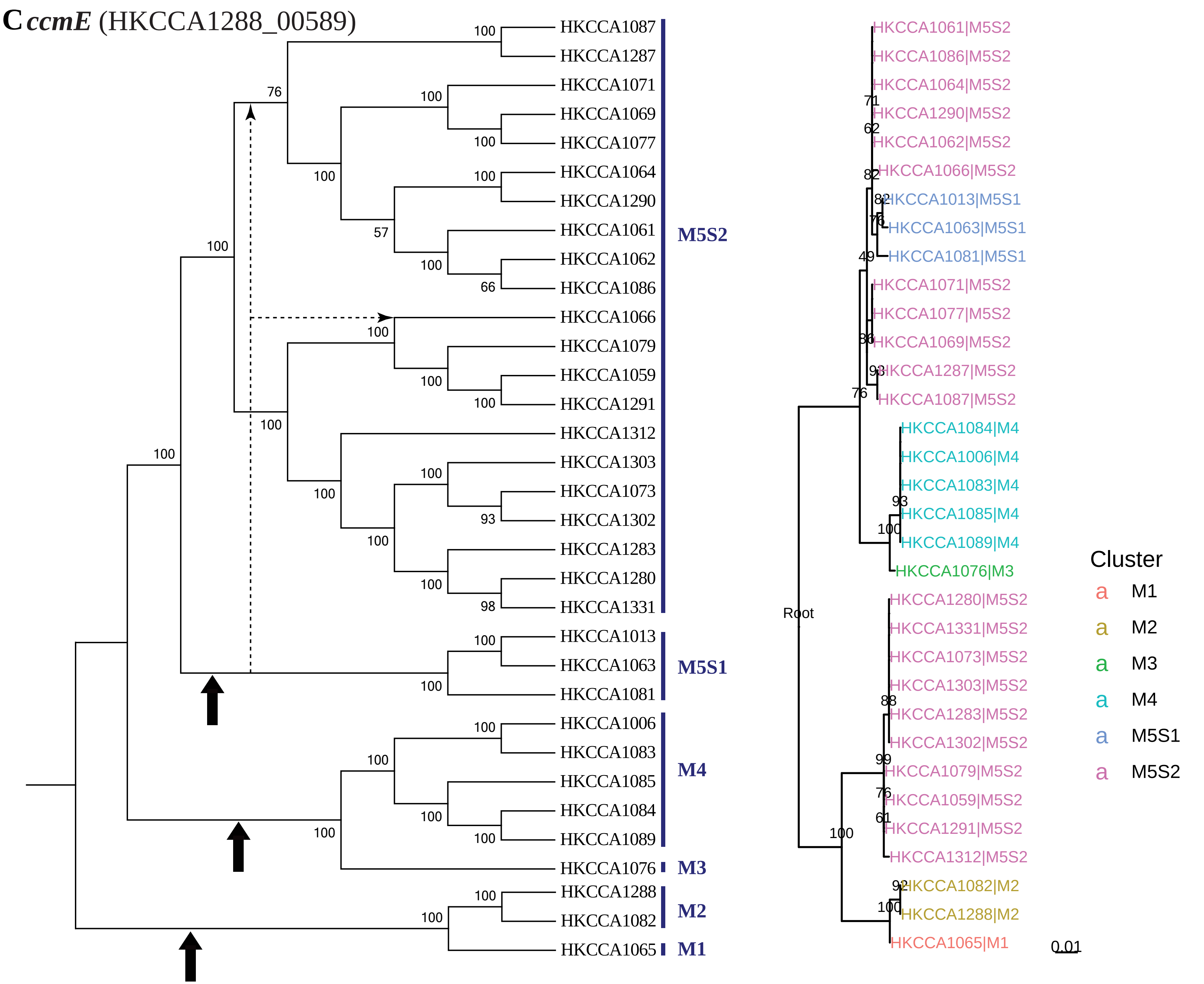
C ccmE (HKCCA1288_00589)
HKCCA1087
HKCCA1287
HKCCA1071
HKCCA1069
HKCCA1077
HKCCA1064
HKCCA1290
HKCCA1061
HKCCA1062
HKCCA1086
HKCCA1066
HKCCA1079
HKCCA1059
HKCCA1291
HKCCA1312
HKCCA1303
HKCCA1073
HKCCA1302
HKCCA1283
HKCCA1280
HKCCA1331
HKCCA1013
HKCCA1063
HKCCA1081
HKCCA1006
HKCCA1083
HKCCA1085
HKCCA1084
HKCCA1089
HKCCA1076
HKCCA1288
HKCCA1082
HKCCA1065
M5S2
M5S1
M4
M3
M2
M1
HKCCA1061|M5S2
HKCCA1086|M5S2
HKCCA1064|M5S2
HKCCA1290|M5S2
HKCCA1062|M5S2
HKCCA1066|M5S2
HKCCA1013|M5S1
HKCCA1063|M5S1
HKCCA1081|M5S1
HKCCA1071|M5S2
HKCCA1077|M5S2
HKCCA1069|M5S2
HKCCA1287|M5S2
HKCCA1087|M5S2
HKCCA1084|M4
HKCCA1006|M4
HKCCA1083|M4
HKCCA1085|M4
HKCCA1089|M4
HKCCA1076|M3
HKCCA1280|M5S2
HKCCA1331|M5S2
HKCCA1073|M5S2
HKCCA1303|M5S2
HKCCA1283|M5S2
HKCCA1302|M5S2
HKCCA1079|M5S2
HKCCA1059|M5S2
HKCCA1291|M5S2
HKCCA1312|M5S2
HKCCA1082|M2
HKCCA1288|M2
HKCCA1065|M1
Root
0.01
Cluster
a M1
a M2
a M3
a M4
a M5S1
a M5S2

D ***purB*** (HKCCA1288_00110)

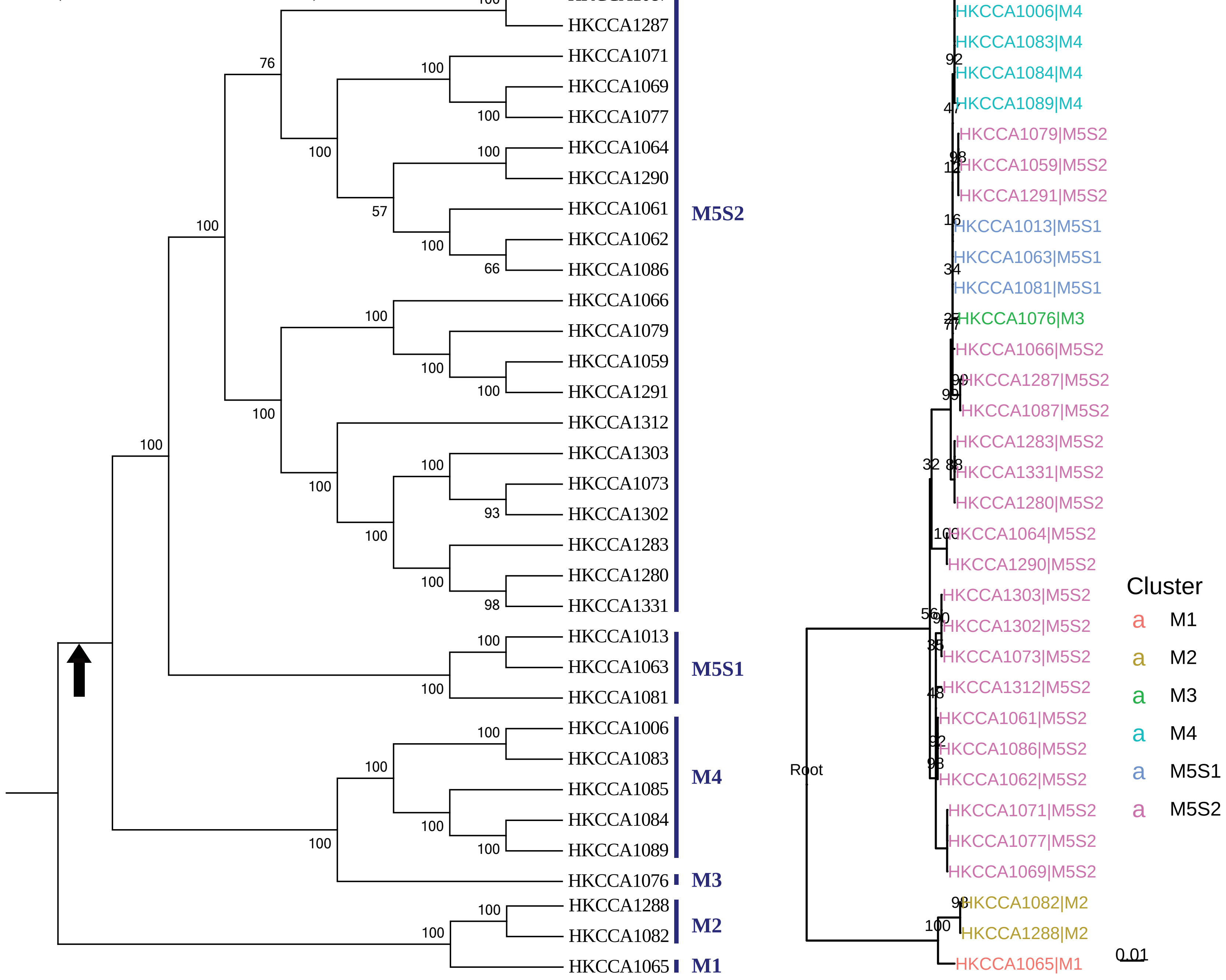

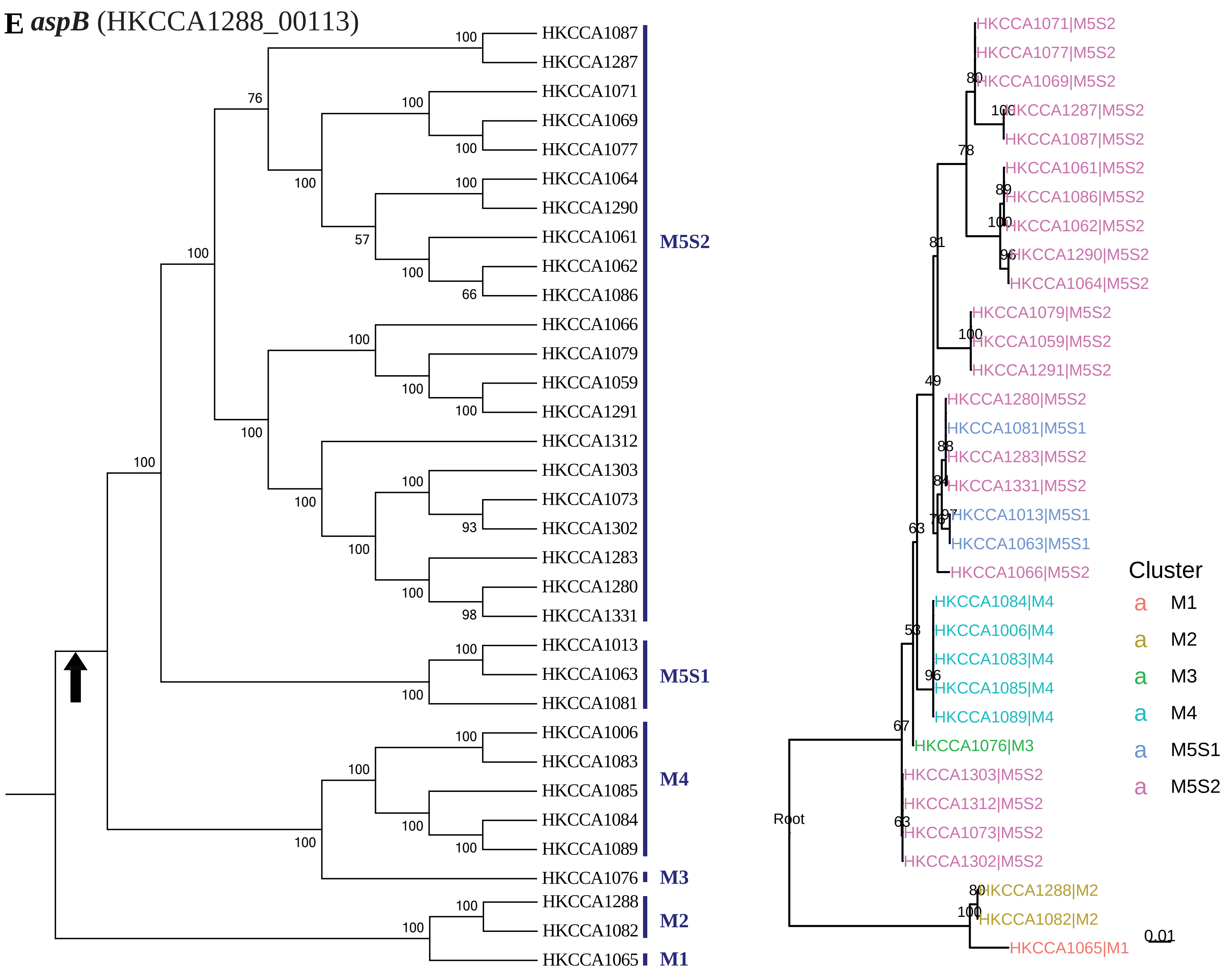
E aspB (HKCCA1288_00113)
HKCCA1087
HKCCA1287
HKCCA1071
HKCCA1069
HKCCA1077
HKCCA1064
HKCCA1290
HKCCA1061
HKCCA1062
HKCCA1086
HKCCA1066
HKCCA1079
HKCCA1059
HKCCA1291
HKCCA1312
HKCCA1303
HKCCA1073
HKCCA1302
HKCCA1283
HKCCA1280
HKCCA1331
HKCCA1013
HKCCA1063
HKCCA1081
HKCCA1006
HKCCA1083
HKCCA1085
HKCCA1084
HKCCA1089
HKCCA1076
HKCCA1288
HKCCA1082
HKCCA1065
M5S2
M5S1
M4
M3
M2
M1
Root
0.01
Cluster
M1
M2
M3
M4
M5S1
M5S2

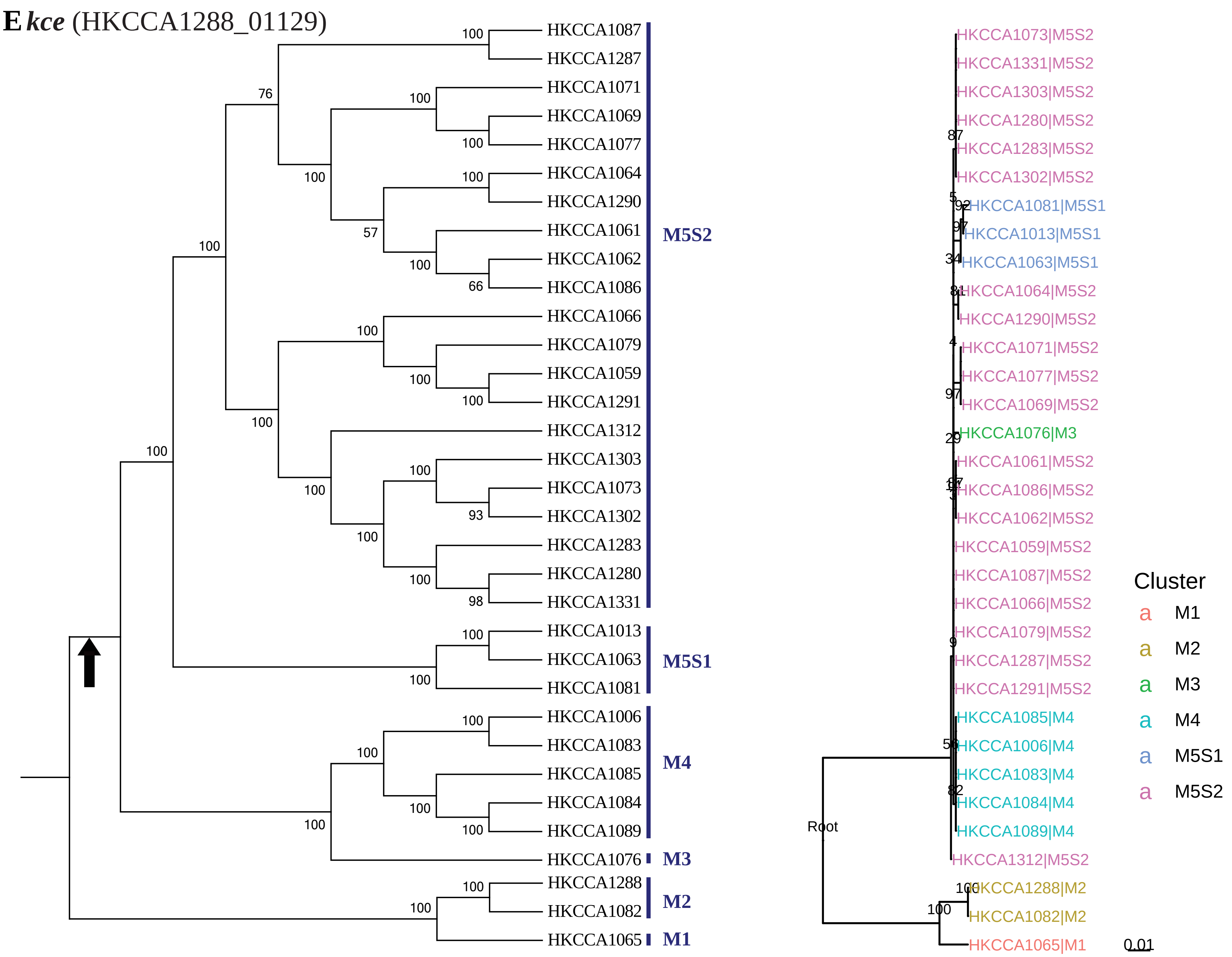
E kce (HKCCA1288_01129)
HKCCA1087
HKCCA1287
HKCCA1071
HKCCA1069
HKCCA1077
HKCCA1064
HKCCA1290
HKCCA1061
HKCCA1062
HKCCA1086
HKCCA1066
HKCCA1079
HKCCA1059
HKCCA1291
HKCCA1312
HKCCA1303
HKCCA1073
HKCCA1302
HKCCA1283
HKCCA1280
HKCCA1331
HKCCA1013
HKCCA1063
HKCCA1081
HKCCA1006
HKCCA1083
HKCCA1085
HKCCA1084
HKCCA1089
HKCCA1076
HKCCA1288
HKCCA1082
HKCCA1065
M5S2
M5S1
M4
M3
M2
M1
HKCCA1073|M5S2
HKCCA1331|M5S2
HKCCA1303|M5S2
HKCCA1280|M5S2
HKCCA1283|M5S2
HKCCA1302|M5S2
HKCCA1081|M5S1
HKCCA1013|M5S1
HKCCA1063|M5S1
HKCCA1064|M5S2
HKCCA1290|M5S2
HKCCA1071|M5S2
HKCCA1077|M5S2
HKCCA1069|M5S2
HKCCA1076|M3
HKCCA1061|M5S2
HKCCA1086|M5S2
HKCCA1062|M5S2
HKCCA1059|M5S2
HKCCA1087|M5S2
HKCCA1066|M5S2
HKCCA1079|M5S2
HKCCA1287|M5S2
HKCCA1291|M5S2
HKCCA1085|M4
HKCCA1006|M4
HKCCA1083|M4
HKCCA1084|M4
HKCCA1089|M4
HKCCA1312|M5S2
HKCCA1288|M2
HKCCA1082|M2
HKCCA1065|M1
Root
0.01
Cluster
M1
M2
M3
M4
M5S1
M5S2

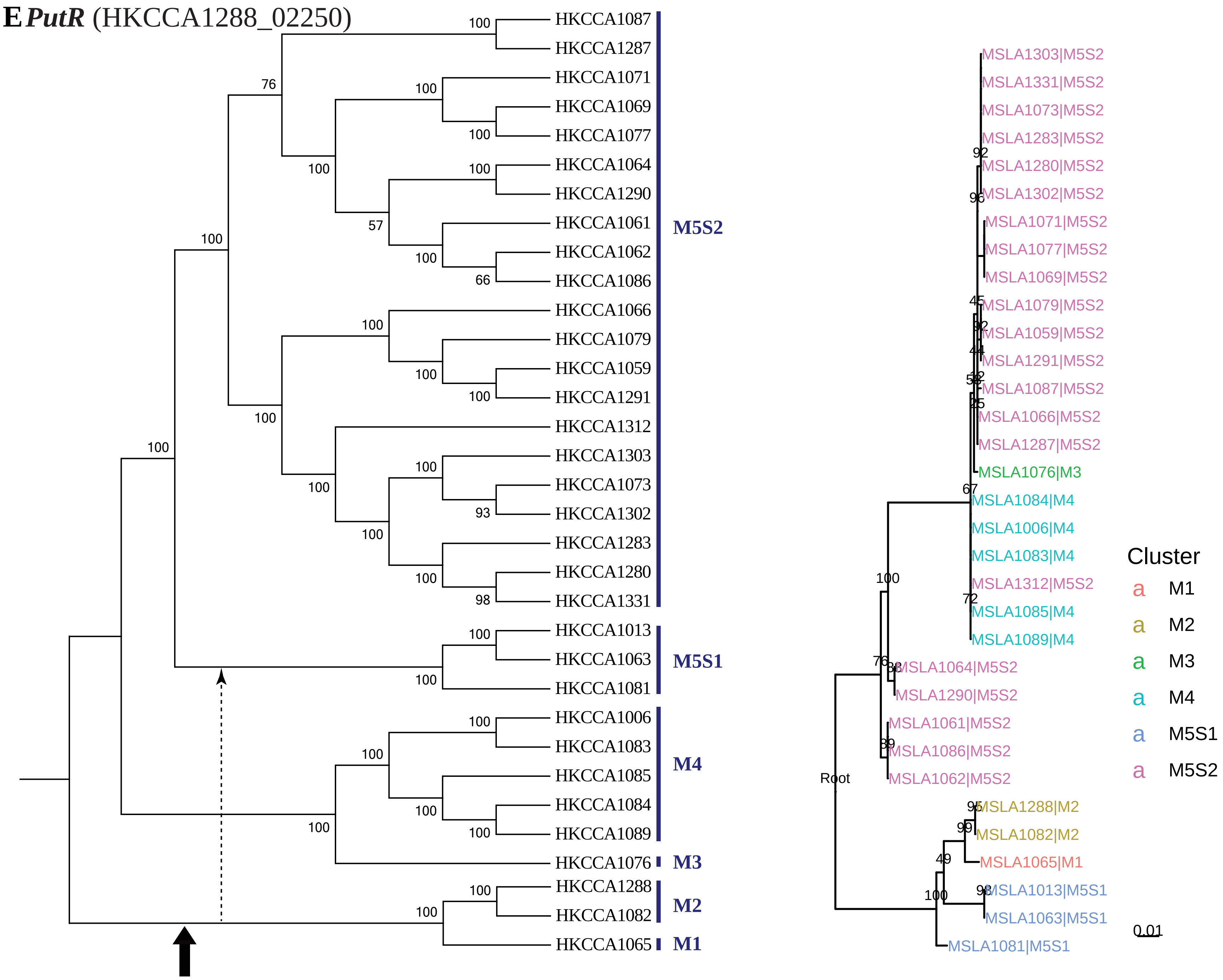
E PutR (HKCCA1288_02250)
HKCCA1087
HKCCA1287
HKCCA1071
HKCCA1069
HKCCA1077
HKCCA1064
HKCCA1290
HKCCA1061
HKCCA1062
HKCCA1086
HKCCA1066
HKCCA1079
HKCCA1059
HKCCA1291
HKCCA1312
HKCCA1303
HKCCA1073
HKCCA1302
HKCCA1283
HKCCA1280
HKCCA1331
M5S2
HKCCA1013
HKCCA1063
HKCCA1081
M5S1
HKCCA1006
HKCCA1083
HKCCA1085
HKCCA1084
HKCCA1089
M4
HKCCA1076
M3
HKCCA1288
HKCCA1082
M2
HKCCA1065
M1
MSLA1303|M5S2
MSLA1331|M5S2
MSLA1073|M5S2
MSLA1283|M5S2
MSLA1280|M5S2
MSLA1302|M5S2
MSLA1071|M5S2
MSLA1077|M5S2
MSLA1069|M5S2
MSLA1079|M5S2
MSLA1059|M5S2
MSLA1291|M5S2
MSLA1087|M5S2
MSLA1066|M5S2
MSLA1287|M5S2
MSLA1076|M3
MSLA1084|M4
MSLA1006|M4
MSLA1083|M4
MSLA1312|M5S2
MSLA1085|M4
MSLA1089|M4
MSLA1064|M5S2
MSLA1290|M5S2
MSLA1061|M5S2
MSLA1086|M5S2
MSLA1062|M5S2
Root
MSLA1288|M2
MSLA1082|M2
MSLA1065|M1
MSLA1013|M5S1
MSLA1063|M5S1
MSLA1081|M5S1
0.01
Cluster
a M1
a M2
a M3
a M4
a M5S1
a M5S2

F DNA-binding protein (HKCCA1288_00114)

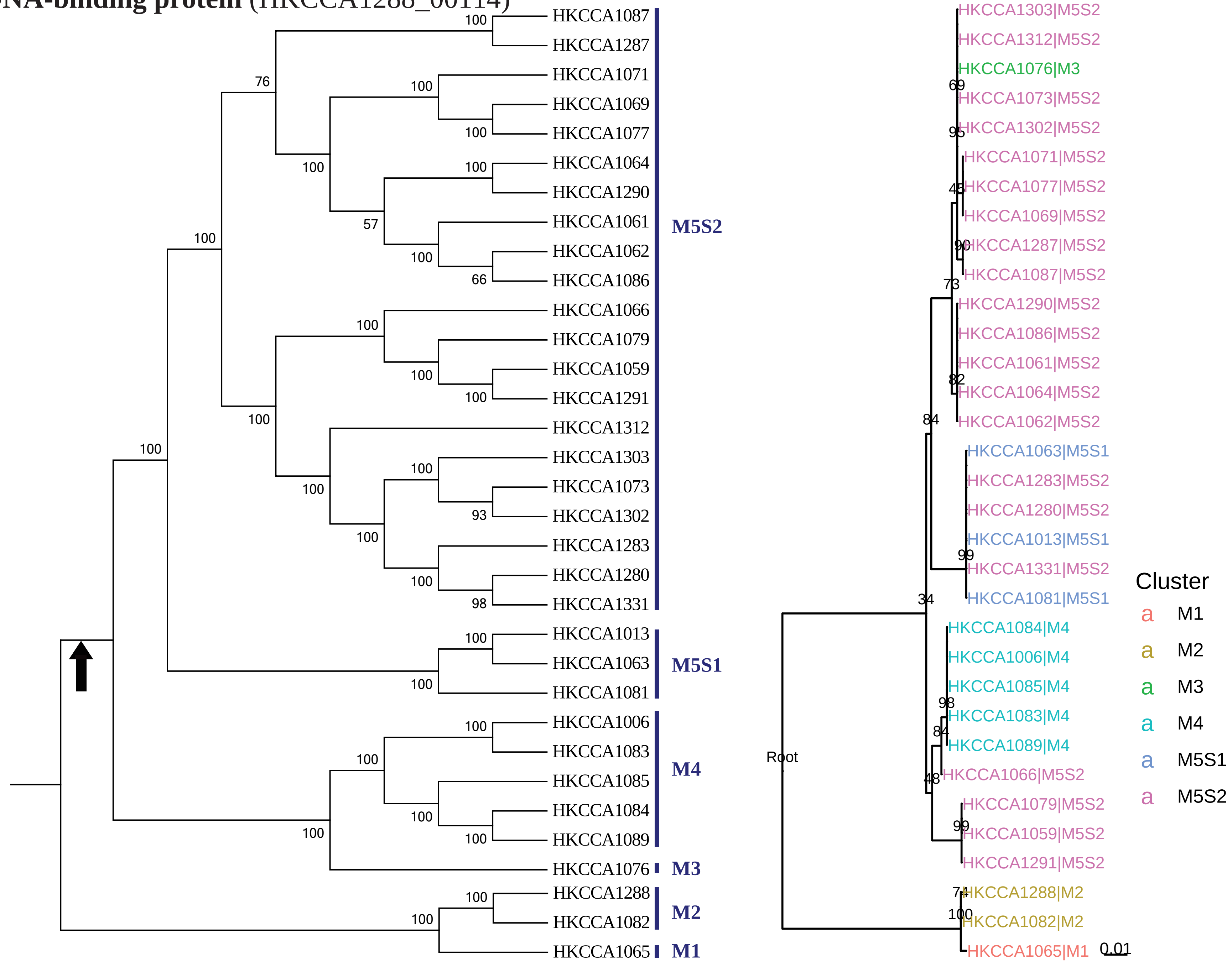

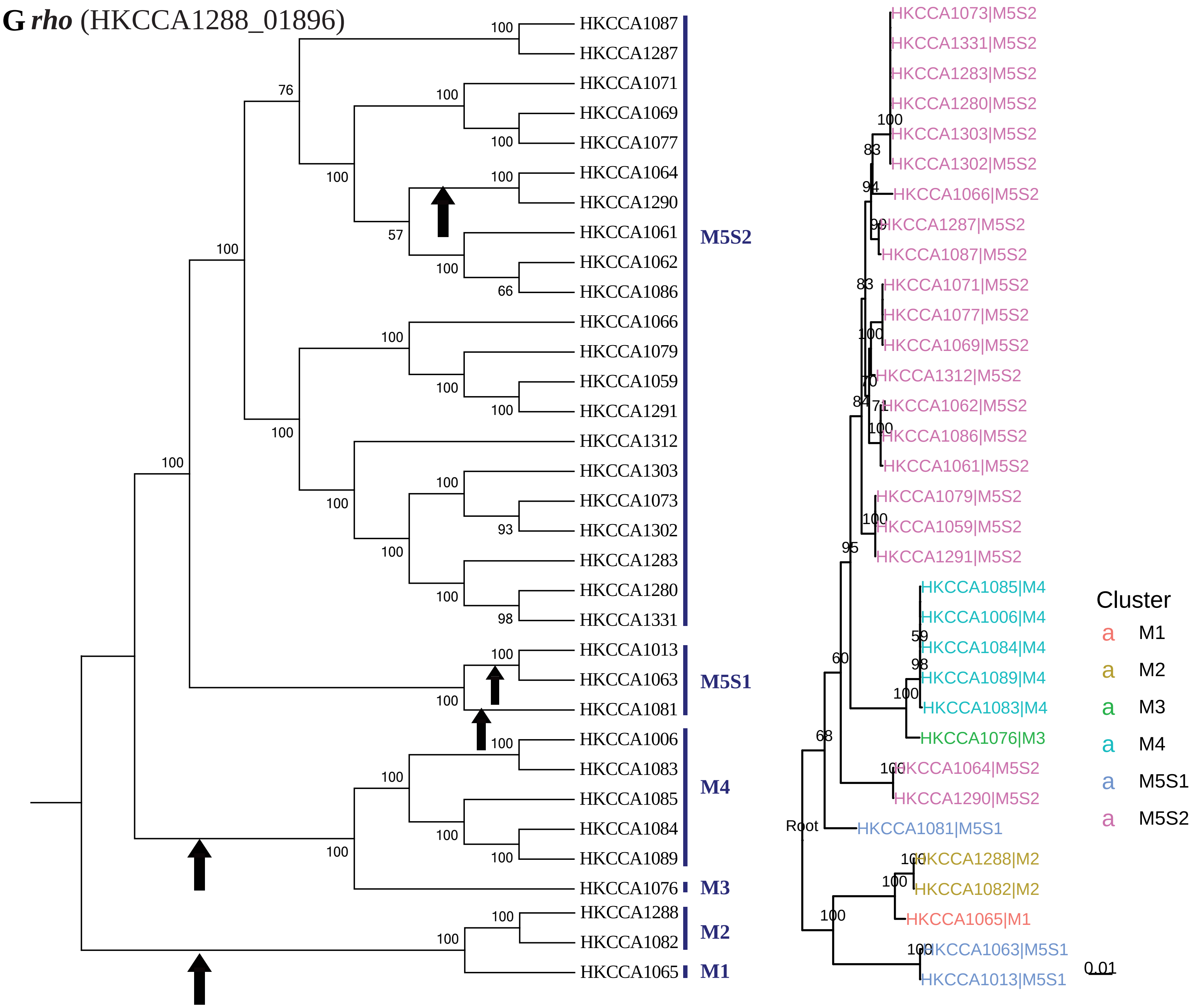

G rho (HKCCA1288_01896)
HKCCA1087
HKCCA1287
HKCCA1071
HKCCA1069
HKCCA1077
HKCCA1064
HKCCA1290
HKCCA1061
HKCCA1062
HKCCA1086
HKCCA1066
HKCCA1079
HKCCA1059
HKCCA1291
HKCCA1312
HKCCA1303
HKCCA1073
HKCCA1302
HKCCA1283
HKCCA1280
HKCCA1331
HKCCA1013
HKCCA1063
HKCCA1081
HKCCA1006
HKCCA1083
HKCCA1085
HKCCA1084
HKCCA1089
HKCCA1076
HKCCA1288
HKCCA1082
HKCCA1065
M5S2
M5S1
M4
M3
M2
M1
HKCCA1073|M5S2
HKCCA1331|M5S2
HKCCA1283|M5S2
HKCCA1280|M5S2
HKCCA1303|M5S2
HKCCA1302|M5S2
HKCCA1066|M5S2
HKCCA1287|M5S2
HKCCA1087|M5S2
HKCCA1071|M5S2
HKCCA1077|M5S2
HKCCA1069|M5S2
HKCCA1312|M5S2
HKCCA1062|M5S2
HKCCA1086|M5S2
HKCCA1061|M5S2
HKCCA1079|M5S2
HKCCA1059|M5S2
HKCCA1291|M5S2
HKCCA1085|M4
HKCCA1006|M4
HKCCA1084|M4
HKCCA1089|M4
HKCCA1083|M4
HKCCA1076|M3
HKCCA1064|M5S2
HKCCA1290|M5S2
HKCCA1081|M5S1
HKCCA1288|M2
HKCCA1082|M2
HKCCA1065|M1
HKCCA1063|M5S1
HKCCA1013|M5S1
Root
0.01
Cluster
a M1
a M2
a M3
a M4
a M5S1
a M5S2

H mandelate racemase (HKCCA1288_02471)

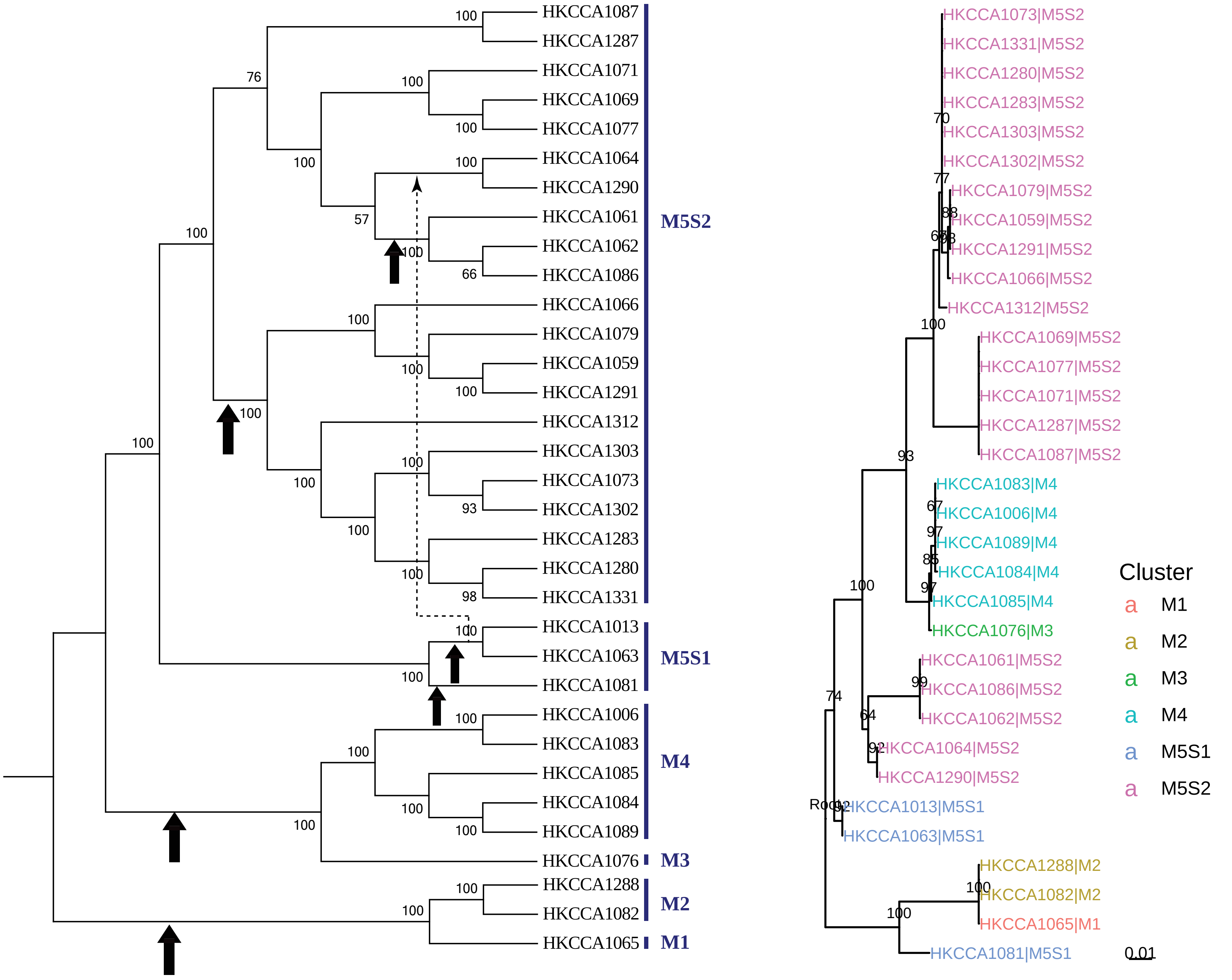

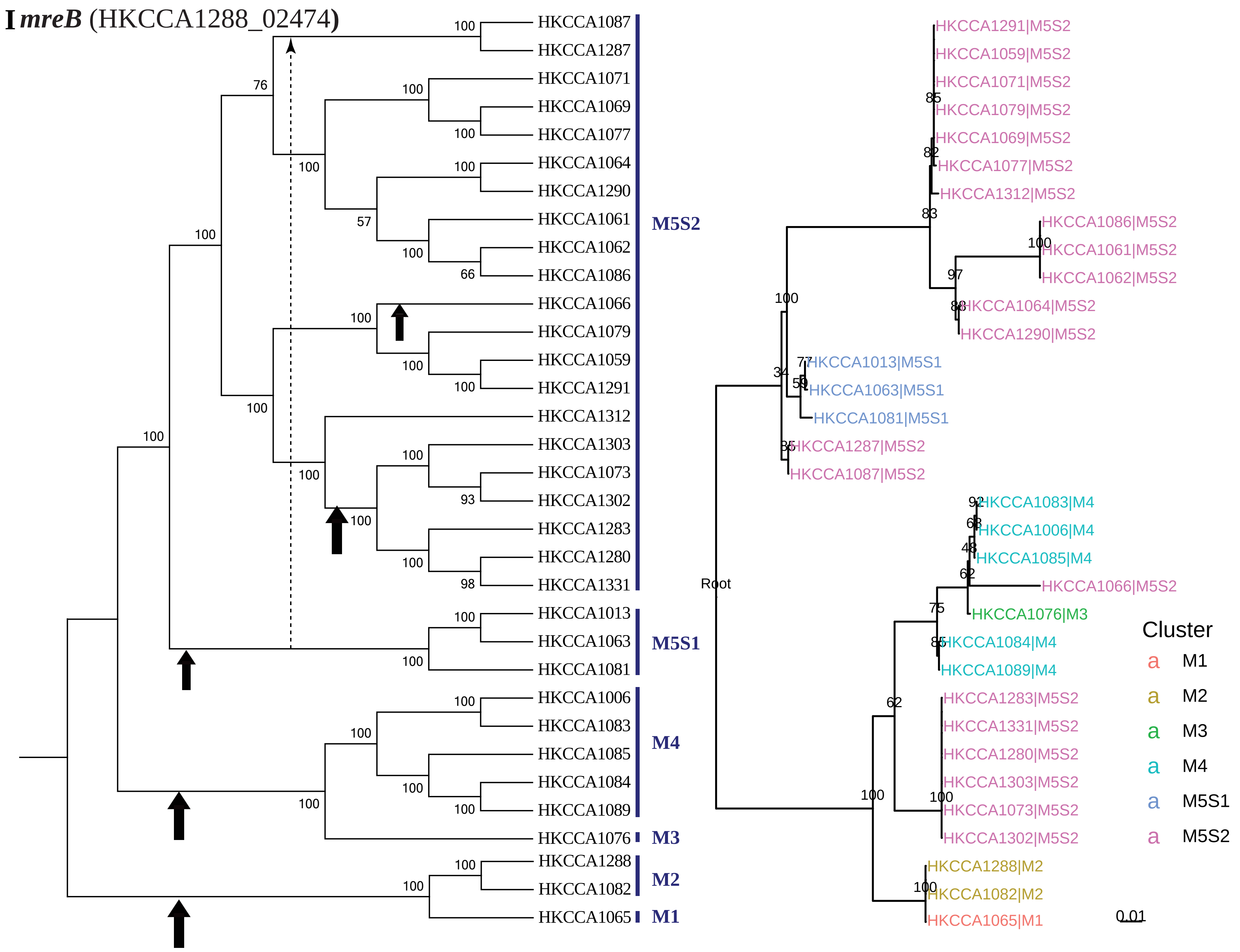
I mreB (HKCCA1288_02474)
HKCCA1087
HKCCA1287
HKCCA1071
HKCCA1069
HKCCA1077
HKCCA1064
HKCCA1290
HKCCA1061
HKCCA1062
HKCCA1086
HKCCA1066
HKCCA1079
HKCCA1059
HKCCA1291
HKCCA1312
HKCCA1303
HKCCA1073
HKCCA1302
HKCCA1283
HKCCA1280
HKCCA1331
HKCCA1013
HKCCA1063
HKCCA1081
HKCCA1006
HKCCA1083
HKCCA1085
HKCCA1084
HKCCA1089
HKCCA1076
HKCCA1288
HKCCA1082
HKCCA1065
M5S2
M5S1
M4
M3
M2
M1
Root
Cluster
M1
M2
M3
M4
M5S1
M5S2
0.01

J Unclassified protein (MSLA1288_01096)

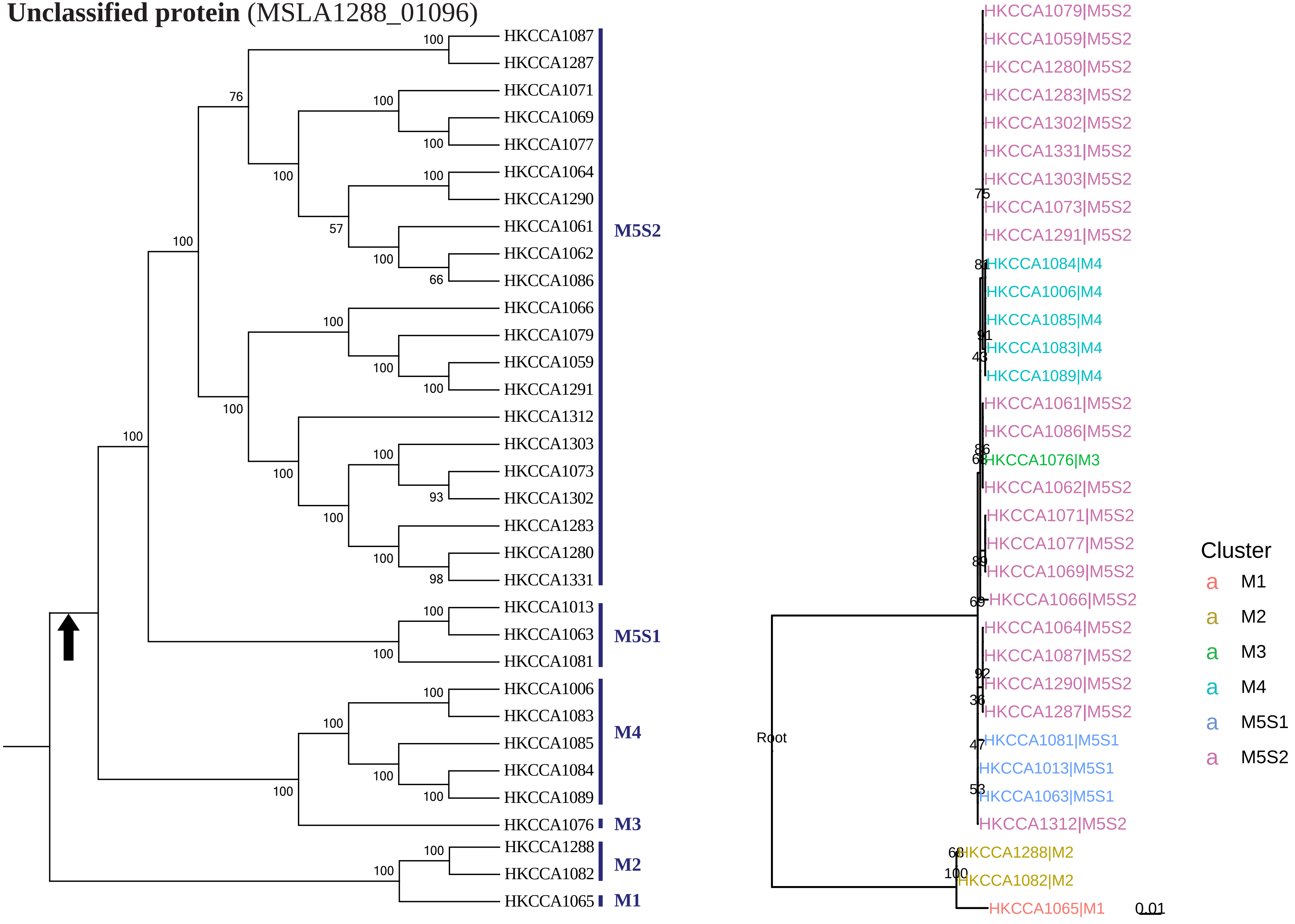

J Unclassified protein (HKCCA1288_00134)

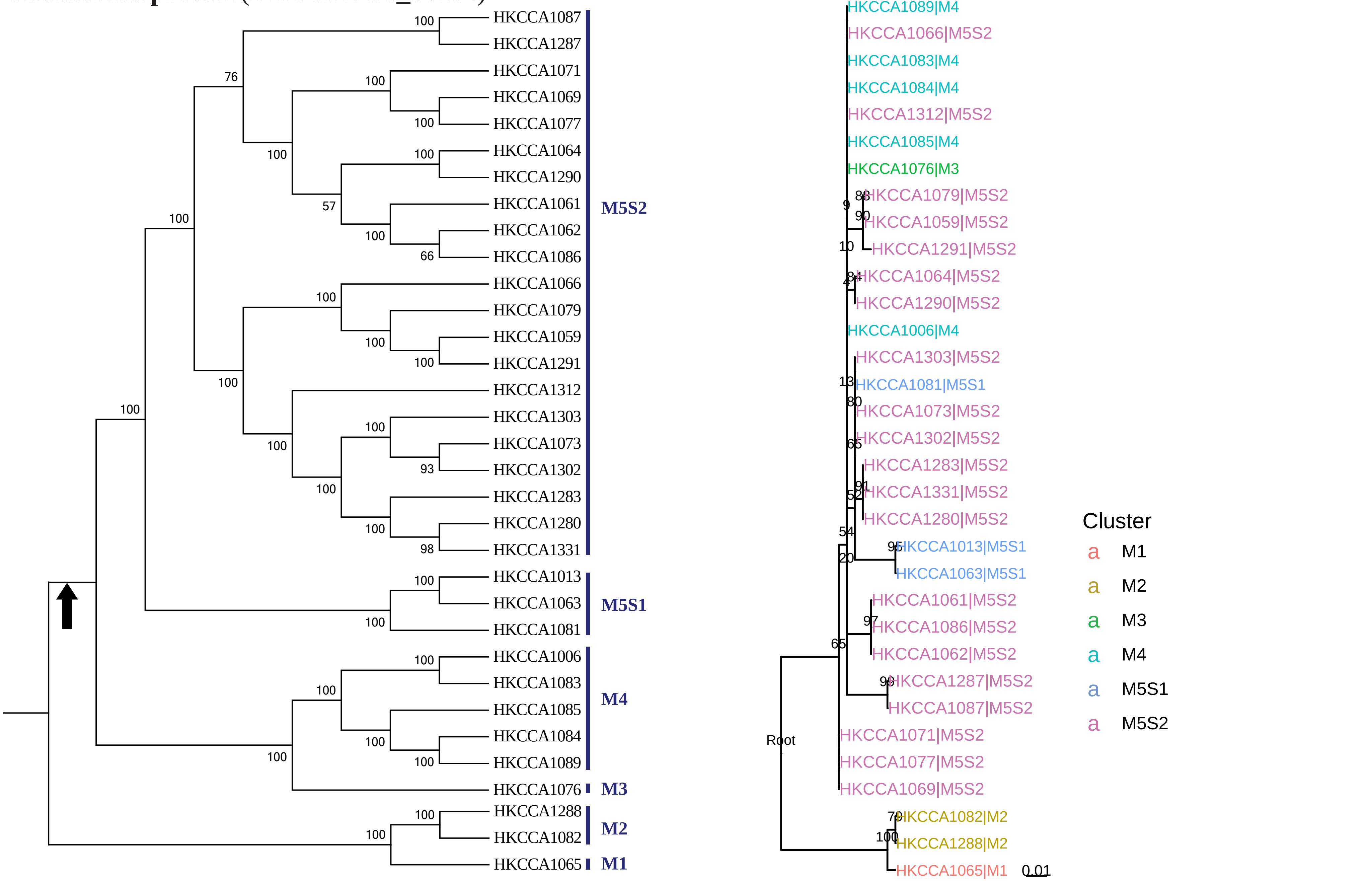

J Unclassified protein (HKCCA1288_01125)

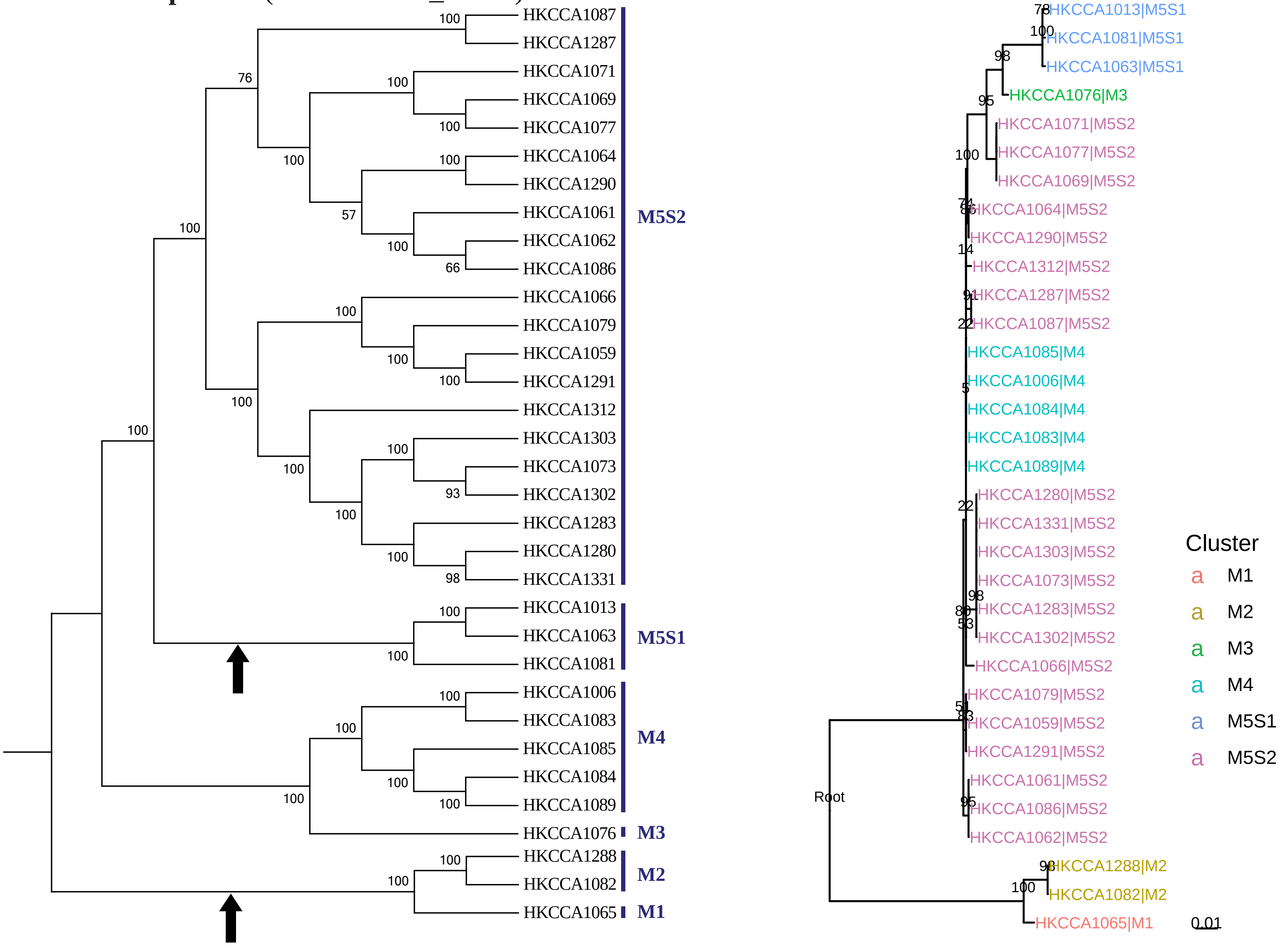

J Unclassified protein (HKCCA1288_01537)

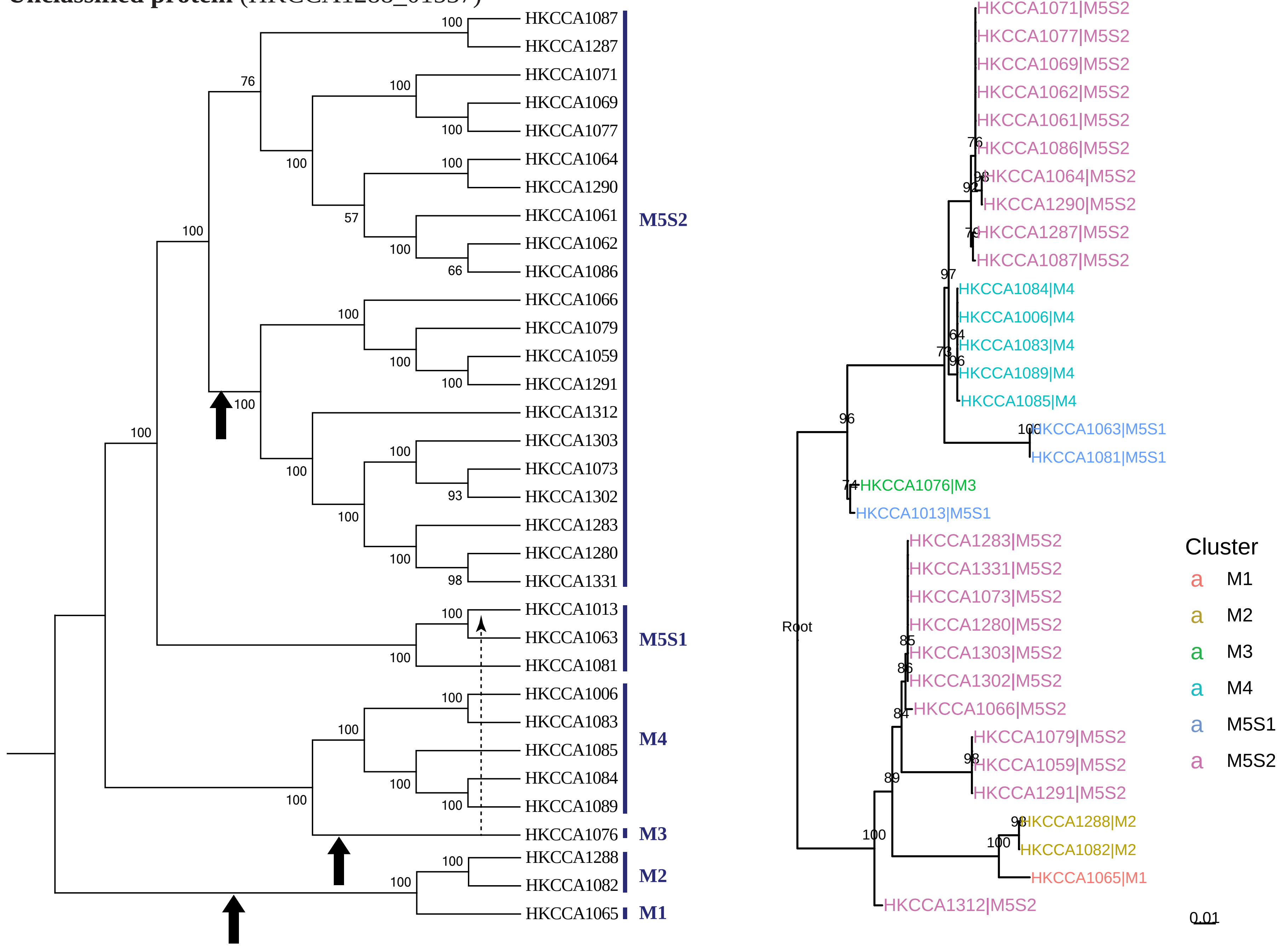

J Unclassified protein (HKCCA1288_00824)

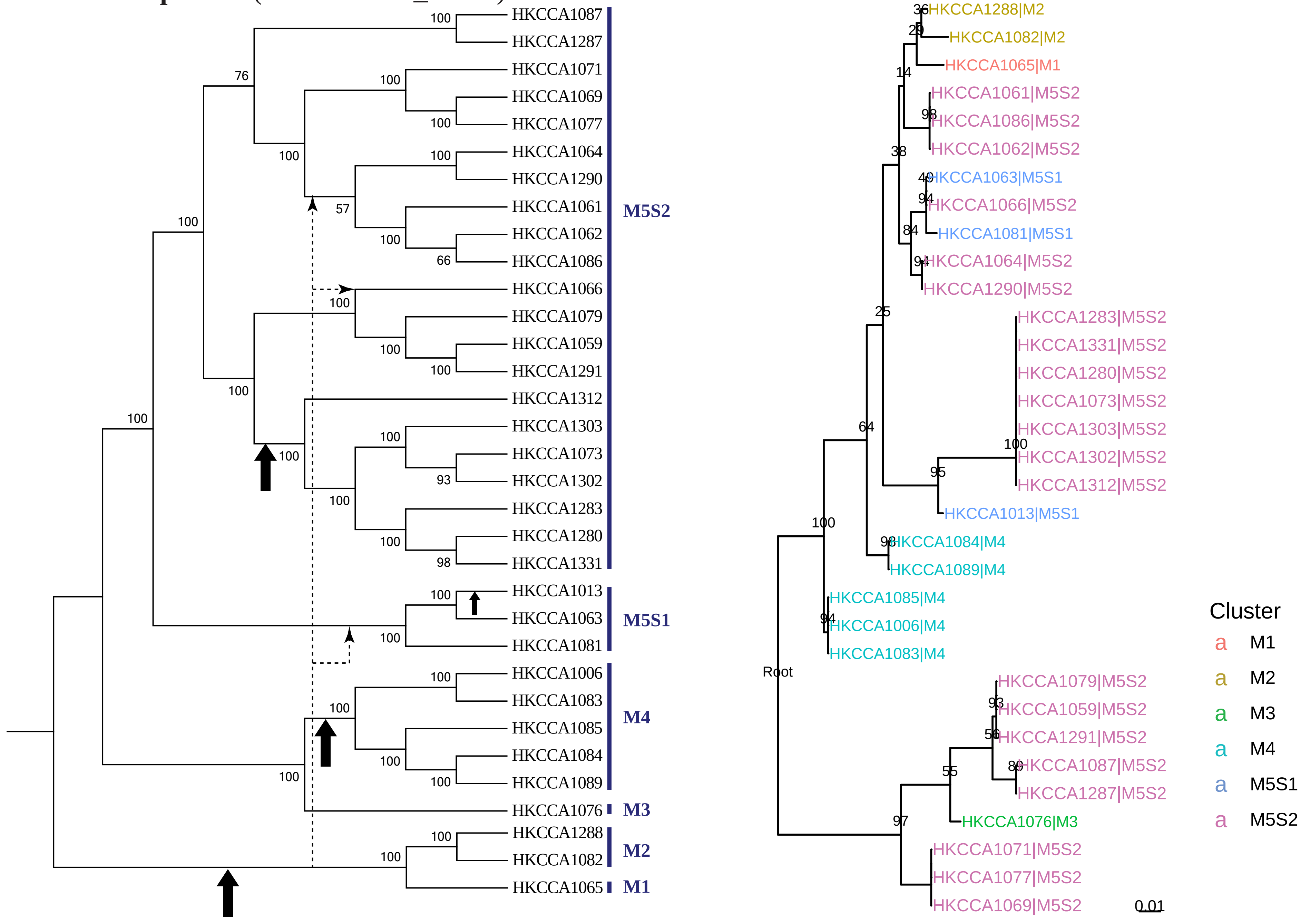

J Unclassified protein (HKCCA1288_02473)

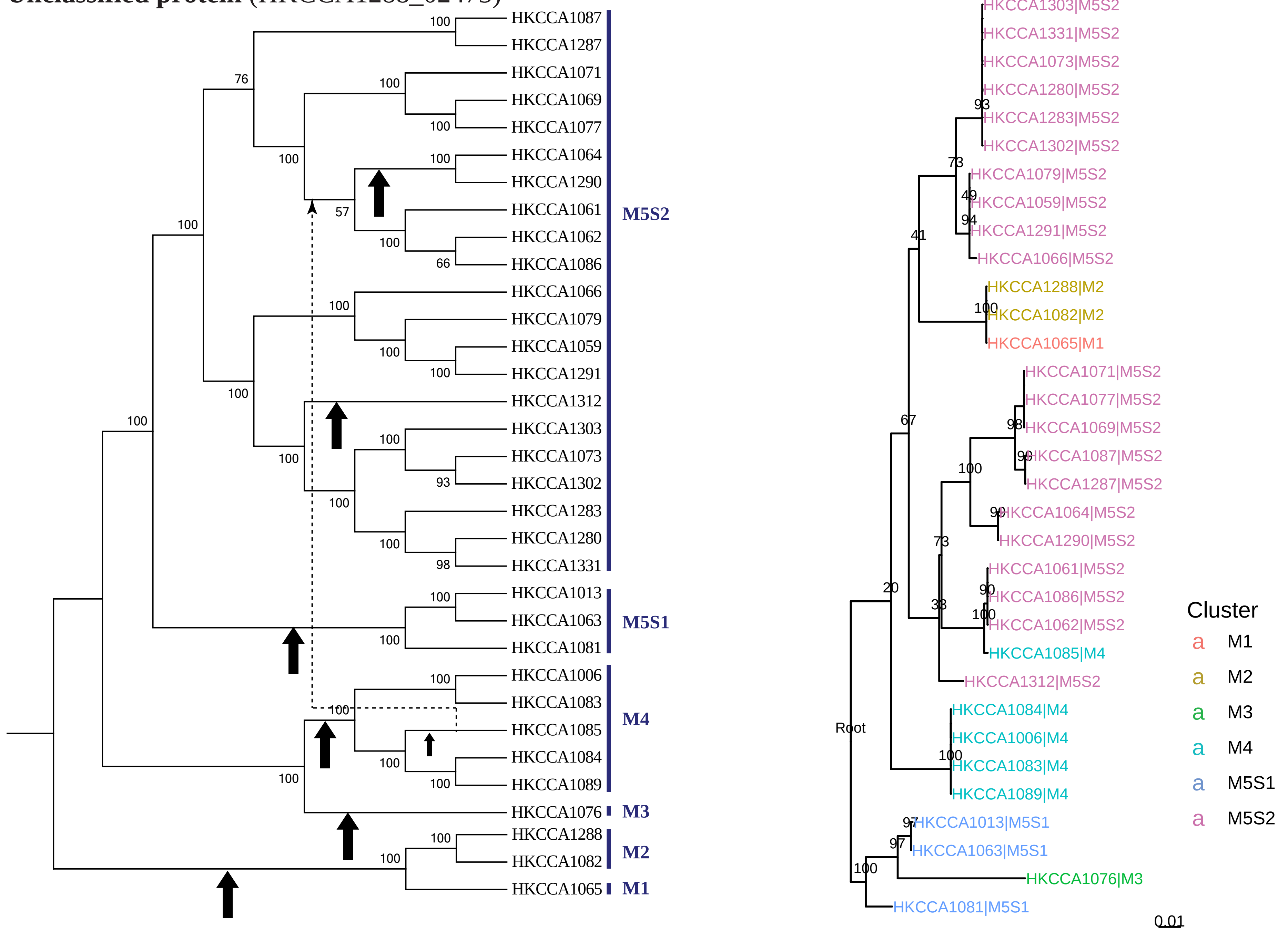

J Unclassified protein (HKCCA1288_01753)

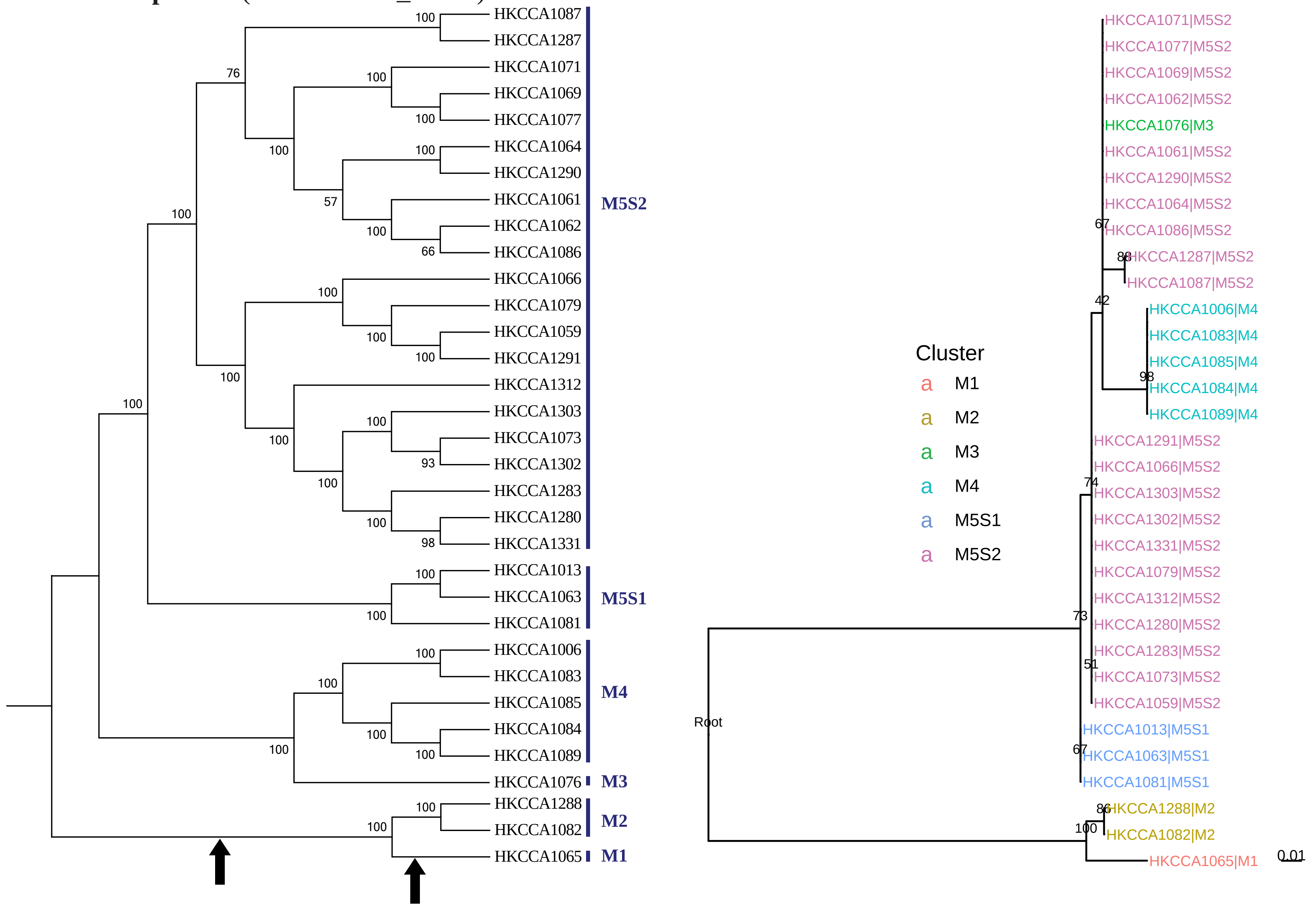

J Unclassified protein (HKCCA1288_01408)

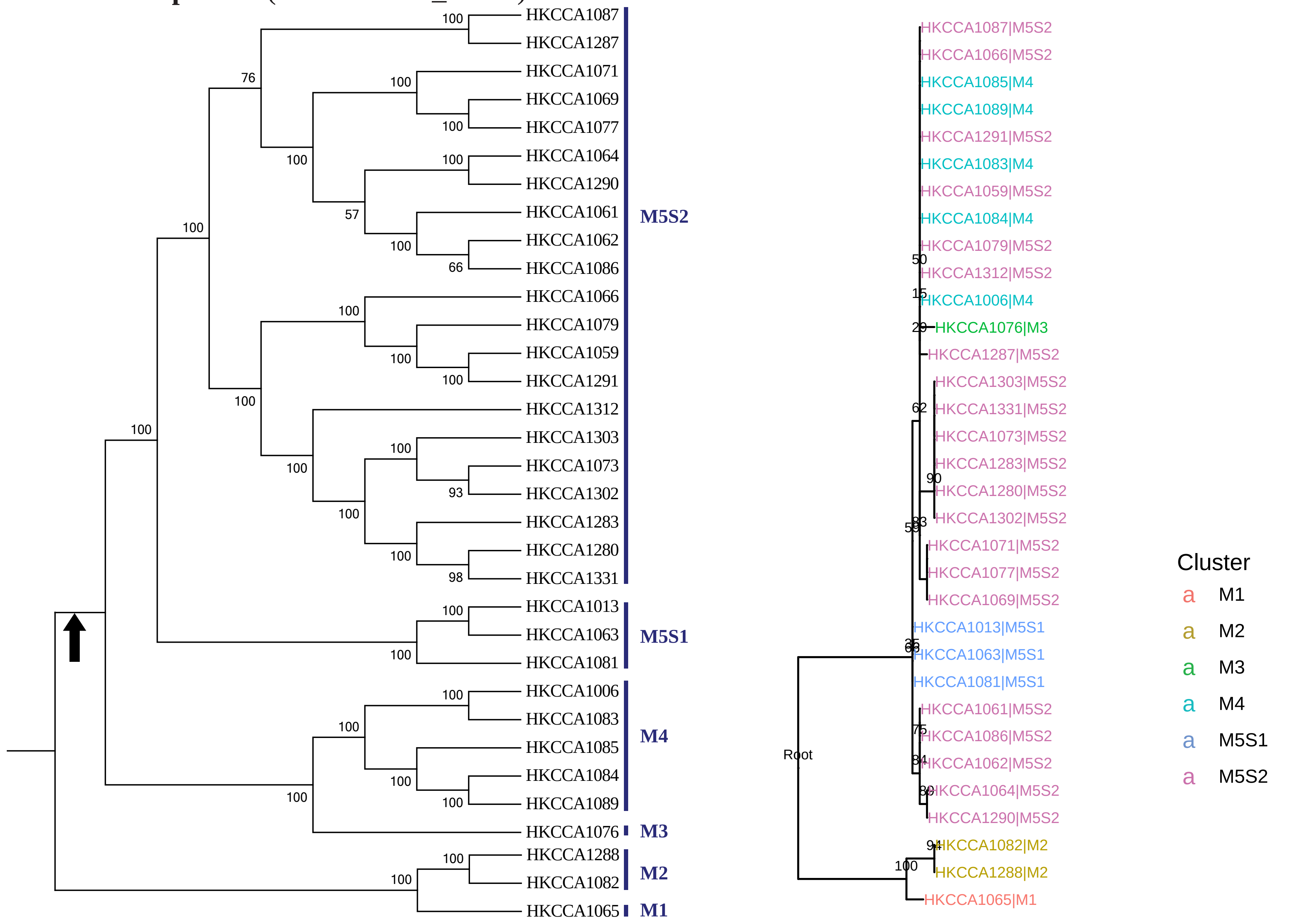

Supplement: FIG S2 [file mbio.00571-22-s0002.pdf]
