# Supplementary material for: Coastal Transient Niches Shape the Microdiversity Pattern of a Bacterioplankton Population with Reduced Genomes
Source: mBio. 2022 Jul 26;13(4):e00571-22. doi: 10.1128/mbio.00571-22 (PMC9426536; doi:10.1128/mbio.00571-22)
Supplement: FIG S3 [file mbio.00571-22-s0003.pdf]

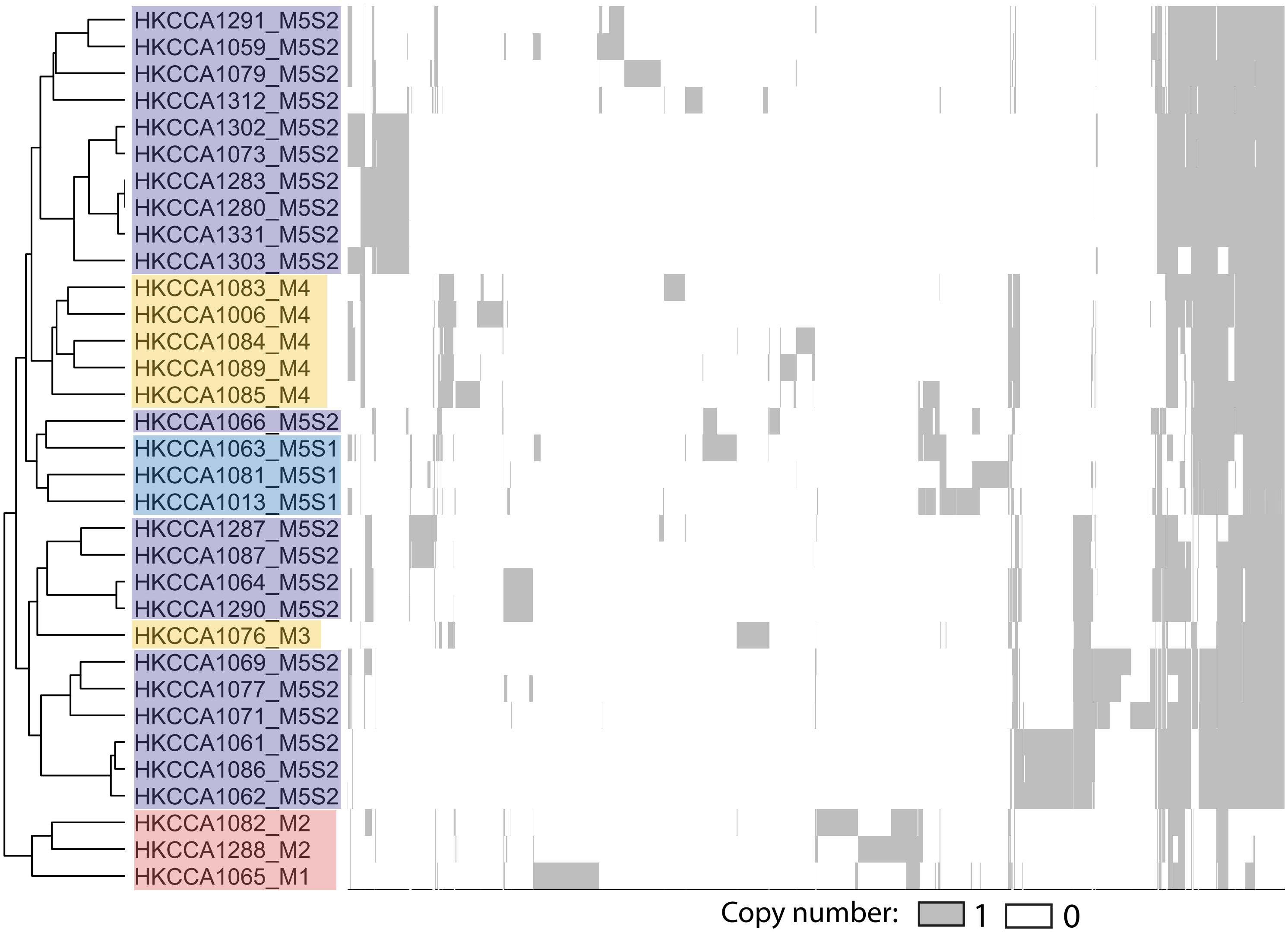

Figure S3. The dendrogram of the 33 CHUG isolates based on the presence and absence of the genes located at the genomic islands.
