# Supplementary material for: Coastal Transient Niches Shape the Microdiversity Pattern of a Bacterioplankton Population with Reduced Genomes
Source: mBio. 2022 Jul 26;13(4):e00571-22. doi: 10.1128/mbio.00571-22 (PMC9426536; doi:10.1128/mbio.00571-22)
Supplement: TEXT S2 [file mbio.00571-22-s0009.docx]

**Text S2. Supplemental Results**

## 2.1 Genetic differentiation of the loci potentially involved in the utilization of organic substrates

The 20 potential carbon sources (Table 1 & Data Set S1j) that may show genotypic differences between M1M2 and M3M4M5 were predicted based on: i) *d_S_* outlier core genes subjected to novel allelic replacements, ii) orthologs specific to either M1M2 or M3M4M5, and iii) different Roary gene families with the same COG annotation. Of these 20 carbon sources, 16 of them are included in BiOLOG phenotype microarrays.

Notably, several *d_S_* outlier genes were related to the metabolism of myo-inositol (*iolG*), β-hydroxybutyric acid (*bdhA*), and D-xylose (*xylD*), all of which can be found in macroalgal ecosystems (Table 1; Data Set S1j). Of these compounds, only β-hydroxybutyric acid was differentially utilized by M1M2 and M3M4M5 (Fig. 3). Gene *bdhA* encodes β-hydroxybutyrate dehydrogenase that catalyzes the reversible conversion between β-hydroxybutyric acid and acetoacetic acid (1). All CHUG members harbor two copies of this gene, but one copy possesses unusually large *d_S_* and thus was subjected to novel allelic replacement (Table 1), which may contribute to the differential utilization of β-hydroxybutyrate (Fig. 3).

For population-specific genes, M1M2-specific genes are involved in the utilization of hydroxypyruvate (*hyi*), myo-inositol (*iolG* and related transporter genes), N-acetylglucosamine (*nagA*), D-fructose (*frcABCR*), and glycerol-3-phosphate (*upgABE*), whereas M3M4M5-specific genes are related to the utilization of D-arabinose (*araD*), maltose (*malRG*), D-glycerate (*garR*), and tartronate semialdehyde (*garR*) (Table 1; Data Set S1j). Of these nine compounds, D-fructose was differentially used by M1 and the remaining populations (Fig. S4), D-arabinose was equally used by M1M2 and M3M4M5 (Fig. S4), myo-inositol, N-acetylglucosamine, glycerol-3-phosphate, and maltose were not used by any CHUG members (Fig. S4 & S5), and hydroxypyruvate, D-glycerate, and tartronate semialdehyde are not included in the phenotype microarrays.

Regarding genes with the same COG annotation, but grouped into different gene families by Roary owing to large genetic divergence (Table 1; Data Set S1j), we identified nine gene pairs of this type that are potentially involved in the utilization of 11 carbon sources. Of these, only L-fucose and acetic acid were differentially utilized by M1M2 and M3M4M5 in the phenotype microarray assay (Fig. 3). L-fucose may be utilized by two alternative pathways (2). The first pathway is enabled by the key gene *fucA* which encodes L-fuculose-phosphate aldolase. It may not be correlated with the differential utilization of L-fucose between M1M2 and M3M4M5, since it is present in all the 33 CHUG members and does not show genetic differentiation between M1M2 and M3M4M5. For the other pathway, while the key gene encoding 2-keto-3-deoxy-L-fuconate dehydrogenase (Data Set S1j) was found in all the 33 CHUG members. it was clustered into two gene families by Roary, each specific to M1M2 and to M3M4M5, respectively, which may be related to the differential utilization of L-fucose. Acetic acid can be degraded by the acetyl-coenzyme A synthetase encoded by the *acs* gene in bacteria (3). While one copy of *acs* was shared by all CHUG members, another copy was found specific to M1M2 and M3M4M5 (Data Set S1j) and its large genetic divergence may explain the differential utilization of acetic acid. While these phenotypic differences may be attributed to the genetic variances, direct evidence supporting the link is not available.

# **References**

1. Wang C, Meek DJ, Panchal P, Boruvka N, Archibald FS, Driscoll BT, Charles TC. 2006. Isolation of poly-3-hydroxybutyrate metabolism genes from complex microbial communities by phenotypic complementation of bacterial mutants. Appl Environ Microbiol 72:384–391.

2. Bunesova V, Lacroix C, Schwab C. 2016. Fucosyllactose and L-fucose utilization of infant *Bifidobacterium longum* and *Bifidobacterium kashiwanohense*. BMC Microbiology 16:248.

3. Brown TD, Jones-Mortimer MC, Kornberg HL. 1977. The enzymic interconversion of acetate and acetyl-coenzyme A in *Escherichia coli*. J Gen Microbiol 102:327–336.
