# Supplementary material for: Coastal Transient Niches Shape the Microdiversity Pattern of a Bacterioplankton Population with Reduced Genomes
Source: mBio. 2022 Jul 26;13(4):e00571-22. doi: 10.1128/mbio.00571-22 (PMC9426536; doi:10.1128/mbio.00571-22)
Supplement: FIG S4 [file mbio.00571-22-s0004.pdf]

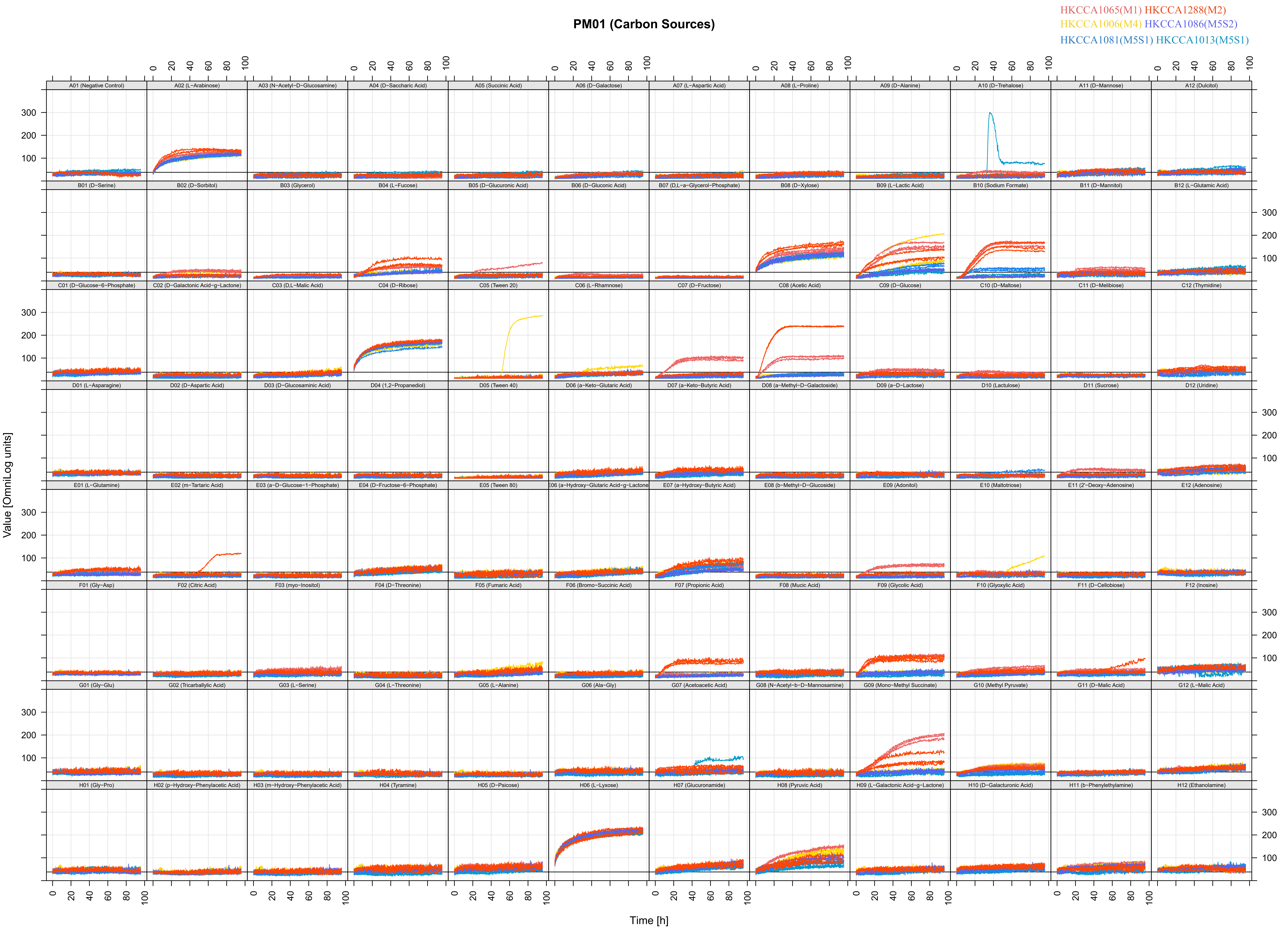

Figure S4. The utilization of 95 carbon sources provided by BiOLOG microplate PM01 by the six representative isolates from different populations. Three replicates were performed for each strain.
