# Supplementary figures and images for: Coastal Transient Niches Shape the Microdiversity Pattern of a Bacterioplankton Population with Reduced Genomes
Source: mBio. 2022 Jul 26;13(4):e00571-22. doi: 10.1128/mbio.00571-22 (PMC9426536; doi:10.1128/mbio.00571-22)

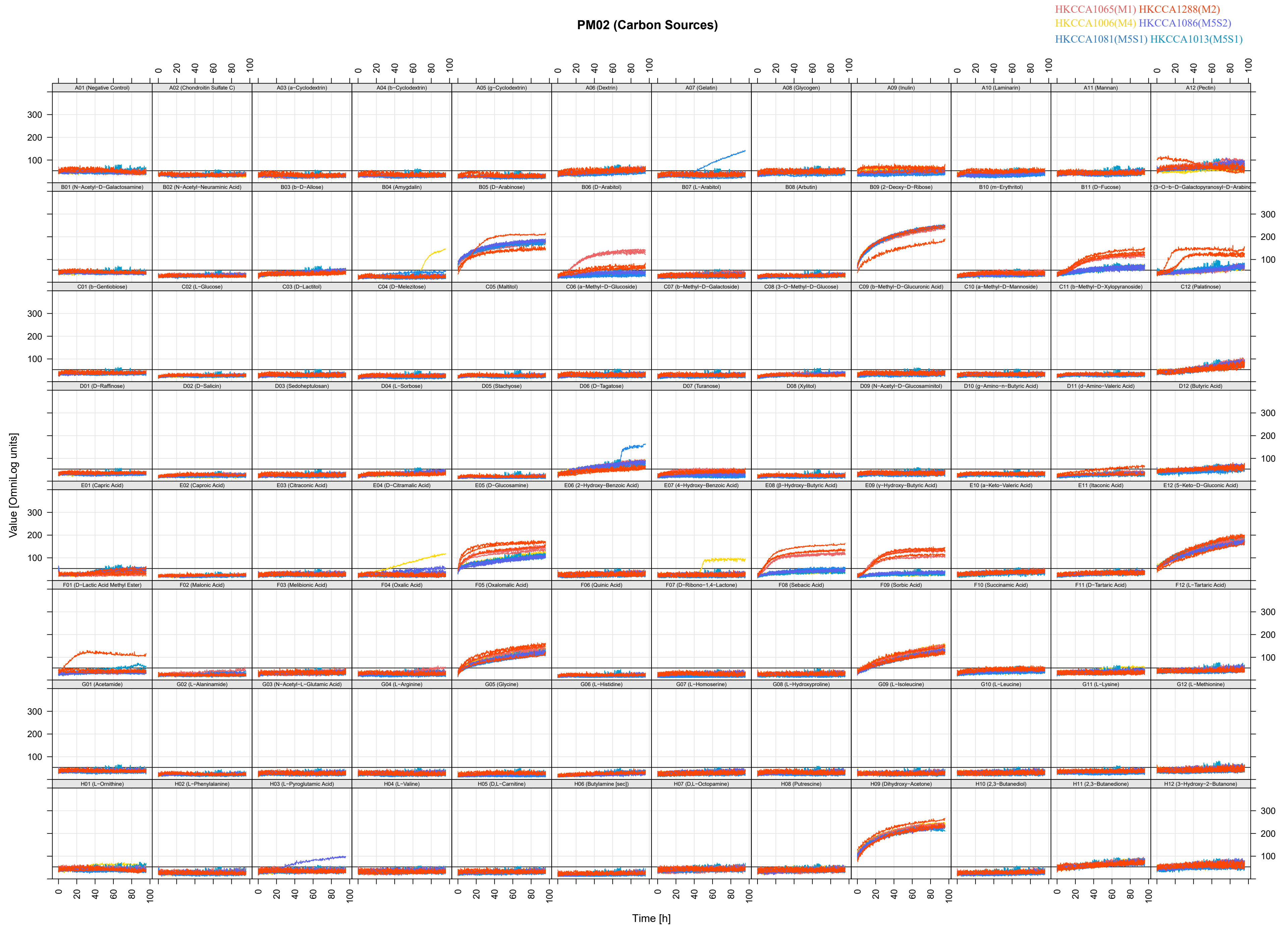

Supplement: FIG S5 [file mbio.00571-22-s0005.pdf]
