# Supplementary material for: Coastal Transient Niches Shape the Microdiversity Pattern of a Bacterioplankton Population with Reduced Genomes
Source: mBio. 2022 Jul 26;13(4):e00571-22. doi: 10.1128/mbio.00571-22 (PMC9426536; doi:10.1128/mbio.00571-22)
Supplement: FIG S6 [file mbio.00571-22-s0006.pdf]

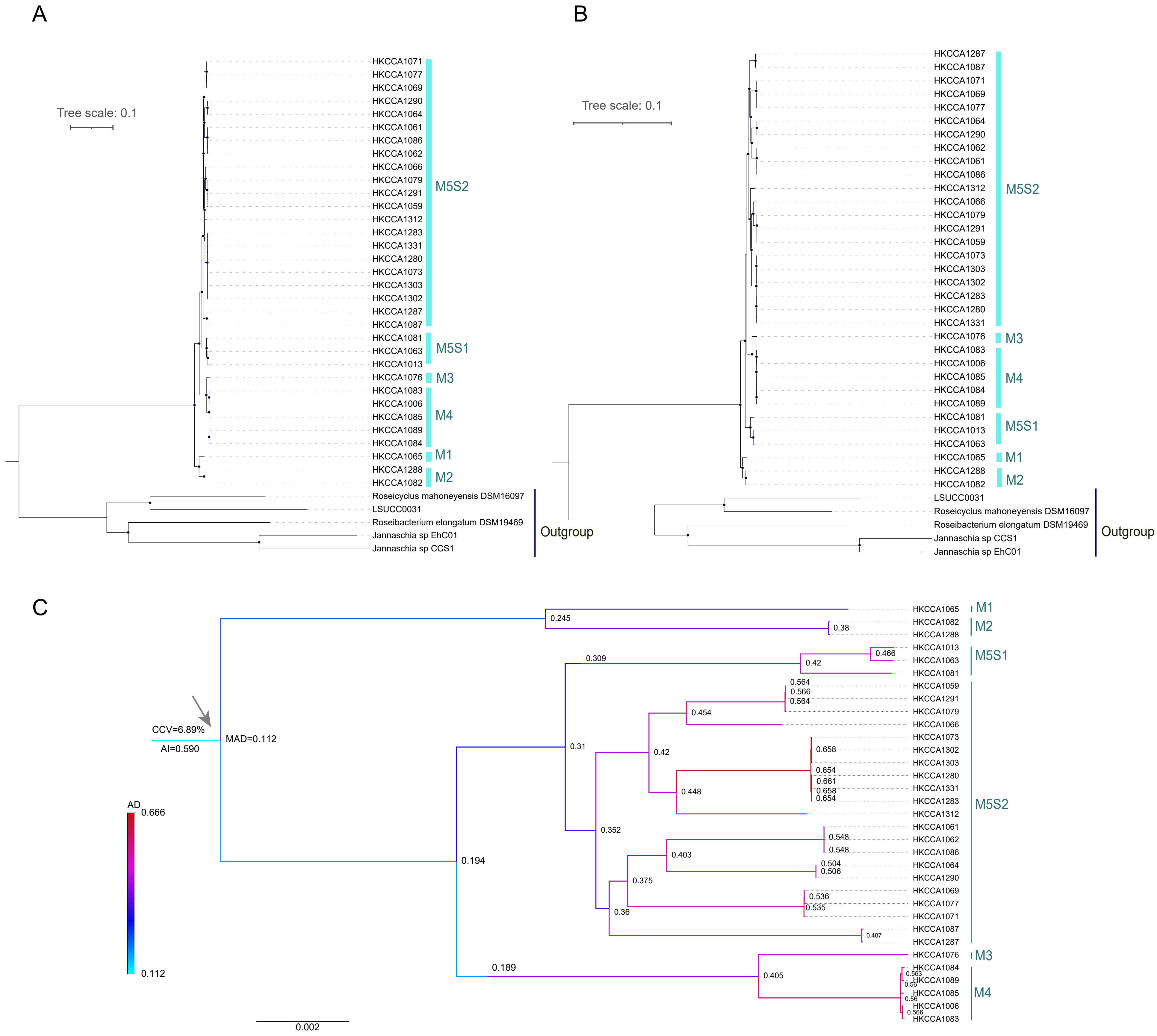

Figure S6. The phylogenomic trees rooted with outgroup-dependent (A and B) and outgroup-free (C) methods. (A) A Maximum Likelihood (ML) phylogenomic tree constructed using IQ-TREE with a shared genomic DNA alignment (including both protein-coding genes and other genomic regions) in which all recombined sites were masked by Gubbins. (B) A ML phylogenomic tree constructed using IQ-TREE with a concatenation of core gene alignment at amino acid level. The root position for the phylogenomic trees in (A and B) was determined with outgroups, which are the sister group of CHUG lineage. Solid circles in the phylogeny indicate nodes with bootstrap values of 100% in the 1,000 bootstrapped replicates. The root position for the phylogenomic tree (B) was determined by minimal ancestor deviation (MAD) method. Branch colors in (B) correspond to their ancestor relative deviation value (AD), which is the relative deviation from the molecular clock expectation. The root clock coefficient of variation (CCV) quantifies the distances from the inferred root to each of the OTUs, and the root ambiguity index (AI) is the ratio of MAD to the second smallest AD. The grey arrow denotes the best root position, which shows the smallest MAD, CCV, and AI values.
